# Smoking as a Risk Factor for Cardiovascular Disease in Females and Males: Observational and Mendelian Randomisation Analyses in the UK Biobank

**DOI:** 10.5334/gh.1485

**Published:** 2025-10-13

**Authors:** Sophie C. de Ruiter, Lena Tschiderer, Diederick E. Grobbee, Patrick Rockenschaub, Ynte M. Ruigrok, Peter Willeit, Hester M. den Ruijter, A. Floriaan Schmidt, Sanne A. E. Peters

**Affiliations:** 1 Julius Center for Health Sciences and Primary Care, University Medical Center Utrecht, Utrecht University, Utrecht, the Netherlands; 2 Institute of Clinical Epidemiology, Public Health, Health Economics, Medical Statistics and Informatics, Medical University of Innsbruck, Innsbruck, Austria; 3 UMC Utrecht Brain Center, Department of Neurology and Neurosurgery, Utrecht University, University Medical Center Utrecht, Utrecht, the Netherlands; 4 Ignaz Semmelweis Institute, Interuniversity Institute for Infection Research, Medical University of Vienna, Vienna, Austria; 5 Department of Public Health and Primary Care, University of Cambridge, Cambridge, United Kingdom; 6 Laboratory of Experimental Cardiology, Department of Cardiology, University Medical Center Utrecht, University Utrecht, Utrecht, the Netherlands; 7 Department of Cardiology, Amsterdam Cardiovascular Sciences, Amsterdam University Medical Centres, University of Amsterdam, Amsterdam, the Netherlands; 8 Institute of Cardiovascular Science, Faculty of Population Health, University College London, London, United Kingdom; 9 Division Heart and Lungs, Department of Cardiology, University Medical Center Utrecht, Utrecht University, Utrecht, the Netherlands; 10 UCL British Heart Foundation Research Accelerator, London, United Kingdom; 11 The George Institute for Global Health, School of Public Health, Imperial College London, London, United Kingdom

**Keywords:** Smoking, cardiovascular disease, Sex differences, Mendelian randomisation

## Abstract

**Introduction:**

Observational studies have shown that smoking is more strongly associated with cardiovascular disease (CVD) in females than in males. It remains unclear whether these observed sex differences reflect differences in the causal effects of smoking between the sexes.

**Methods:**

This study investigated sex-specific associations between ever smoking, smoking continuation, and the number of cigarettes smoked per day and CVD outcomes by conducting sex-stratified observational and Mendelian randomisation (MR) analyses in the UK Biobank.

**Results:**

In observational analyses, we found a greater excess risk of ever smoking, smoking continuation, and number of cigarettes smoked per day for CVD in females than in males with female-to-male ratios of hazard ratios (HRs) of 1.08 (95% confidence interval [CI] 1.04, 1.12), 1.15 (1.07, 1.22), 1.05 (1.02, 1.08), respectively. Results were similar for CHD, and we found no sex differences for stroke. Results from MR analyses were directionally similar; however, we were not able to detect statistically significant sex differences in the effect of smoking exposures on any CVD outcome. For subarachnoid haemorrhage (SAH), we found indications for a stronger causal effect of ever smoking in females as compared to males (female-to-male ratio of ORs 2.61 [95%CI 1.06, 6.42]).

**Conclusion:**

This study shows that both smoking initiation and higher smoking intensity are observationally and causally related to a higher CVD risk in both females and males. Observed sex differences in the association between smoking and CVD were directionally similar to sex differences in the causal effects of smoking on CVD. In general, MR estimates were more uncertain, and the causal effects of smoking on CVD may be similar in females and males.

## What is already known on this topic

Observational studies have shown a stronger association of smoking with cardiovascular disease (CVD) in females than in males; however, it is unclear whether this reflects sex differences in the causal effects of smoking.

## What this study adds

This study found that observed sex differences in the relationship between smoking and CVD are directionally similar to sex differences in causal effects from Mendelian randomisation (MR) analyses. However, MR analyses were more uncertain than observational analyses, and no statistically significant sex differences in causal effects were detected. For subarachnoid haemorrhage, MR analysis indicated a stronger causal effect of ever smoking in females compared to males.

## How this study might affect research, practice or policy

These findings emphasize the importance of smoking reduction in both sexes. Future MR studies using sex-specific genetic data on smoking are needed to assess sex-specific causal effects of smoking.

## Introduction

Smoking is one of the leading modifiable risk factors for cardiovascular disease (CVD), a major cause of death worldwide (1). While smoking is harmful for everyone, various observational studies have shown that the magnitude of that harm is greater in females than in males (2,3,4). For example, a meta-analysis of 75 cohort studies including 2.4 million participants showed that the excess risk of coronary heart disease (CHD) associated with smoking was 25% greater in females as compared to males (3). The excess risk of stroke is at least as great among females who smoke compared with males who smoke, although evidence from Western countries, in which smoking habits in females are more similar to those in males, also suggests a greater excess risk in females (5). Yet, as previous studies on sex differences in the association between smoking and CVD were observational in nature, the causality of the sex differences cannot be established.

Mendelian randomisation (MR) is a method for studying the causal effects of modifiable exposures (e.g., smoking) on health outcomes using genetic variants associated with the specific exposures of interest (6,7). MR has previously shown the causal link between smoking and CVD (8,9,10). However, as the analyses were conducted in a sex-combined manner, it remains unclear whether the magnitude of the causal effect of smoking on CVD is different between the sexes (11). Shedding more light on sex differences and similarities in causal relationships would improve our understanding of the mechanisms behind the development of CVD in both females and males. This, in turn, would enhance the advancement of more targeted prevention strategies for CVD.

Therefore, to better understand the link between smoking behaviour and CVD in females and males, the objective of the current study was to conduct observational and MR analysis on the sex-specific associations and potential causal relationships between smoking and the risk of different types of CVD.

## Methods

The results of this study are presented in accordance with the STROBE = The Strengthening the Reporting of Observational Studies in Epidemiology and STROBE-MR guidelines. The STROBE and STROBE-MR checklists are available in Supplementary Table S1 and Supplementary Table S2.

Data were used from the UK Biobank (UKB), a large-scale prospective study in the UK including over 500,000 participants aged 40 to 69 years, who were recruited between 2006 and 2010 (12,13). Participants attended one of the 22 centres across the UK for a detailed baseline assessment that involved the collection of extensive questionnaire data, physical measurements, and biological samples. The UKB was approved by the North West Multi-Centre Research Ethics Committee (16/NW/0274), and all participants provided written informed consent.

### Study participants

For observational analysis, participants were excluded if they had a history of CVD (defined as CHD or stroke) before baseline, based on data obtained through linkage with routinely collected information from general practitioners, hospital admissions, and self-reported data (from the first occurrence of disease data set released by the UK Biobank (14)). For MR analyses, participants were excluded if they had missing genetic data, a genotyping missing rate of greater than 2%, were related, were outliers based on heterozygosity and missing rates, or had a mismatch between genetic and reported sex. To minimise population stratification, we further excluded participants who did not self-identify as White British and/or did not have a genetically validated White British ancestry based on principal component analysis of the genotypes.

### Definition of smoking exposures

Exposure variables of smoking were ever smoking, smoking continuation, and the number of cigarettes smoked per day. Ever smoking was defined as ever versus never smoking regularly in life. Smoking continuation was defined as current versus former smoking among ever smokers. The number of cigarettes per day was defined as the average number of cigarettes smoked per day among current or former smokers. To allow for comparison between results from MR analysis and observational analysis, the number of cigarettes smoked per day was categorised into the following categories equal to the genome-wide association study (GWAS): 1 = 1–5, 2 = 6–15, 3 = 16–25, 4 = 26–35, 5 = 36+ and was analysed as a continuous variable. Smoking habits in UKB participants were self-reported (Supplemental Methods).

Genetic liability to smoking was defined by selecting genetic variants related to ever smoking, smoking continuation, and the number of cigarettes smoked per day, as determined by the GWAS & Sequencing Consortium of Alcohol and Nicotine use (GSCAN) (Supplemental Methods) (15). Since sex-specific GWAS data on smoking were not available, we used sex-combined GWAS data.

### Genetic data and variant selection

For each exposure, we selected single-nucleotide polymorphism (SNPs) based on a strong association with the exposure as reported by the GWAS (15) using a *p*-value threshold of 5 × 10^–8^, and on a low pairwise linkage disequilibrium (*r*^2^ < 0.001) based on the 1000Genome reference panel, which includes only SNPs with minor allele frequencies higher than 0.01 (17,18). We checked whether genetic variants were reported on the same allele and harmonized the data accordingly. SNPs were excluded if the INFO score was 0.9 or lower and when the SNP was not in Hardy–Weinberg equilibrium using a *p*-value threshold of 1 × 10^–5^ (19). An overview of all selected SNPs per exposure is provided in Supplementary Table S3.

For ever smoking, we selected 248 SNPs based on the *p*-value and linkage disequilibrium, of which we excluded 2 because of an INFO score lower than 0.9, leaving 246 SNPs as instrumental variables. For smoking continuation, we identified 22 SNPs, of which we excluded one because of an INFO score lower than 0.9, leaving 21 SNPs as instrumental variables. For the number of cigarettes per day, we identified 51 SNPs, of which we excluded 3 because of low genotyping quality, leaving 48 SNPs as instrumental variables (Table S4). All SNPs selected as instruments were in Hardy–Weinberg equilibrium.

For genetic associations with outcomes, we obtained individual-level imputed data on genetic variants from the UKB. Participants were genotyped with the Affymetrix UK BiLEVE Axiom array and the Affymetrix UKB Axiom Array (13,16). Genotype imputation was performed using the Haplotype Reference Consortium and the UK10K haplotype reference panel for the UKB (20).

### Definition of endpoints

Cardiovascular outcomes were defined using *International Classification of Diseases, Tenth Revision* (ICD-10) codes obtained from general practitioners’ data, hospital admission data, and death registries (from the first occurrence of disease data set released by the UK Biobank (14)), with the same ascertainment method and outcome definition applied to events occurring both before and after study entry. CVD was defined using ICD-10 codes I20-I25, I60, I61, I63, and I64. We conducted separate analyses on major CVD outcomes: CHD (I20–I25), acute myocardial infarction (MI) (I21), stroke (I60, I61, I63, I64), ischaemic stroke (I63, I64), intracerebral haemorrhage (ICH) (I61), and subarachnoid haemorrhage (SAH) (I60).

For MR analyses, all events before and after study entry were considered, because they can be treated as incident since they occur after conception. For the observational analyses, we only considered incident events after study entry, with follow-up endingat the first occurrence of the CVD event, death, loss to follow-up, or December 31, 2022, whichever occurred first.

### Statistical analysis

#### Observational analyses

Sex-specific and sex-combined hazard ratios (HR) with corresponding 95% confidence intervals (CIs) for the associations between smoking phenotypes and CVD were estimated by performing Cox regression analysis using age as the underlying time scale. We adjusted for the Townsend deprivation index (an area-based measure of socioeconomic status), sex, and an interaction term between the Townsend deprivation index and sex. We did not adjust our primary observational analysis for other cardiovascular risk factors because these were considered potential mediators as suggested previously (2,4). However, we conducted a sensitivity analysis, additionally adjusting for systolic blood pressure, antihypertensive medication, total cholesterol, high-density lipoprotein cholesterol, C-reactive protein (log-transformed), type 2 diabetes mellitus, and body mass index, as well as interaction terms between sex and these factors. We also included a sex interaction term with each of the smoking exposures to obtain the female-to-male ratio of HRs (RHR) and the corresponding 95%CIs. An RHR > 1 indicates a stronger association in females as compared to males, an RHR < 1 suggests a stronger association in males as compared to females, and an RHR = 1 indicates no sex differences in the HRs.

Missing values were imputed separately for females and males using multiple imputation by chained equations. This was carried out with 20 imputed data sets and 30 iterations for each sex (Supplemental Methods).

To ensure that differences between observational and MR results were not affected by potential variations between populations, we conducted a sensitivity analysis for the observational analyses on the same population as was used for the MR analyses.

#### MR analyses

Genetic associations of selected variants with the study outcomes were estimated using logistic regression in the UKB, both in the overall population and for each sex separately. We adjusted for age at baseline, sex (if appropriate), and the first 16 genetic principal components (21).

In our primary MR analysis, we performed inverse-variance weighting. We used the Cochran Q test to assess the heterogeneity between estimates obtained using different variants and MR-Egger to investigate potential horizontal pleiotropy (i.e., when the Egger intercept deviates from zero). In addition, we performed simple median regression, weighted median regression, and MR Pleiotropy Residual Sum and Outlier (MR-PRESSO) to assess the robustness of MR results (22). We used the MR-PRESSO distortion test to assess differences in estimates before and after excluding outlier variants that were detected by MR-PRESSO.

To compare sex-specific ORs, we obtained female-to-male ratios of OR (RORs) by subtracting log ORs for males from log ORs for females. *p*-values for sex differences were obtained from Cochrane’s Q test. An ROR > 1 indicates a stronger association in females than in males, an ROR < 1 suggests a stronger association in males than in females, and an ROR = 1 indicates no sex differences in the ORs.

To assess instrumental variable strength, we report the *F*-statistics (Supplemental Methods). The number of selected SNPs for each exposure and estimate, with corresponding *F*-statistics, are provided in Supplementary Table S4. *F*-statistics ranged between 7.1 and 18.7.

Statistical analyses were conducted using R version 4.0.5 (R Foundation, Vienna, Austria). MR analyses were conducted using the R-package *MendelianRandomization* (23) and *MR-PRESSO* (22). All tests were two-sided, and *p*-values of 0.05 or less were considered statistically significant.

## Results

Of all UKB participants eligible for inclusion, 468,838 participants had no history of CVD and were included in observational analyses. The mean age at recruitment was 56 (SD 8) years and 56% were female (Table 1). At baseline, females were less likely to have ever smoked (40% of females vs. 50% in males) and both sexes smoked at a similar intensity (median of 15 cigarettes a day, 25th–75th percentile 10–20 in current female and male smokers).

**Table 1:** Characteristics of study population in observational analysis.


CHARACTERISTICS	NO. OF NON-MISSING VALUES IN FEMALES	FEMALES (*n* = 261,920)	NO. OF NON-MISSING VALUES IN MALES	MALES (*n* = 206,918)

Age, years	261,920	56.1 (8.0)	206,918	56.2 (8.2)

Ethnicity	260,714		205,570	

White		246,775 (94.2)		194,382 (93.9)

Other*		13,909 (5.3)		111,70 (5.4)

Smoking status	260,514		205,697	

Never smoker		156,428 (59.7)		103,982 (50.3)

Former smoker		81,109 (31.0)		76,082 (36.8)

Current smoker		22,977 (8.8)		25,633 (12.4)

Number of cigarettes smoked daily in current smokers	17,309	15 [10, 20]	15,710	15 [10, 20]

Cigarettes smoked per day in current or former smokers	68,730		65,131	

1–5		6,421 (2.5)		3,420 (1.7)

6–15		32,463 (12.4)		24,593 (11.9)

16–25		24,527 (9.4)		28,310 (13.7)

26–35		3,542 (1.4)		6,605 (3.2)

≥36		1,797 (0.7)		5,633 (2.7)

Socioeconomic status	261,606		206,649	

Townsend deprivation index score		–2.17 [–3.6, 0.4]		–2.17 [–3.7, 0.5]

Townsend deprivation thirds				

Low (≥1.40)		49,097 (18.7)		40,676 (19.7)

Middle (≥–2.08 – <1.40)		78,601 (30.0)		60,374 (29.2)

High (<–2.08)		133,908 (51.1)		105,599 (51.0)

Outcomes (first occurrence after study entry)				

Cardiovascular disease		19,076 (7.3)		28,768 (13.9)

Coronary heart disease		14,450 (5.5)		23,458 (11.3)

Myocardial infarction		3,192 (1.2)		6,820 (3.3)

Stroke		4,626 (1.8)		5,310 (2.6)

Ischaemic stroke		3,392 (1.3)		4,388 (2.1)

Intracerebral haemorrhage		709 (0.3)		664 (0.3)

Subarachnoid haemorrhage		525 (0.2)		258 (0.1)


Numbers are presented as mean (standard deviation), median [25^th^, 75^th^ percentile], or number (percentage). *Includes Asian or Asian British, Indian, Pakistani, Bangladeshi, any other Asian background, Chinese, black or black British, Caribbean, African, any other black background, other ethnic group, white and black Caribbean, white and black African, white and Asian, and any other mixed background.

Over a median follow-up of 13.7 years (25th–75th percentile, 12.8–14.5), 19,076 (7.3%) females and 28,768 (13.9%) males experienced the combined CVD endpoint, including 14,450 (5.5%) females and 23,458 (11.3%) males with CHD (of which 3,192 [1.2%] females and 6,820 [3.3%] males with MI) and 4,626 (1.8%) females and 5,310 (2.6%) males with stroke (of which 3,392 [1.3%] females with ischaemic stroke, 4,388 [2.1%] males with ischaemic stroke, 709 [0.3%] females with ICH, 664 [0.3%] males with ICH, 525 [0.2%] females with SAH, and 258 [0.1%] males with SAH).

Of all UKB participants eligible for inclusion, 337,386 participants were included in MR analyses. Baseline characteristics of this population are presented in Supplementary Table S5.

### Ever smoking

**Figure 1:**
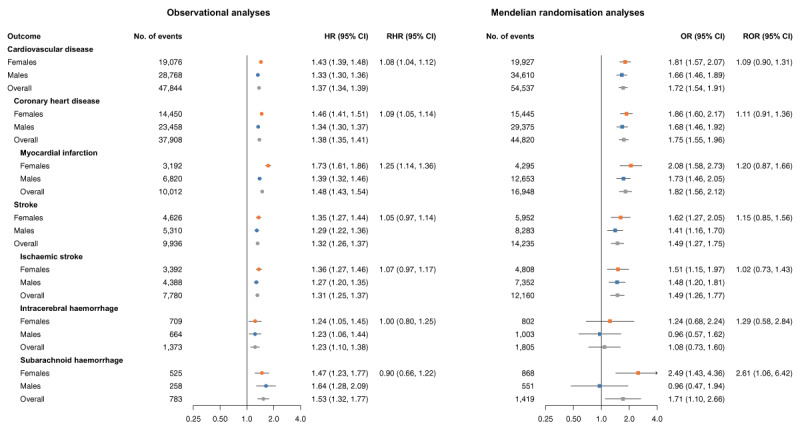
Cox regression and Mendelian randomisation analysis of the association between ever smoking and risk of cardiovascular events in females, males, and the overall population. MR estimates are from inverse-variance weighted MR, and ORs can be interpreted as the effect per unit increase in log odds of genetic liability to ever smoking. MR analyses were performed in 337,386 UK Biobank participants. Cox regressions were performed in 468,838 UK Biobank participants and were adjusted for sex and Townsend deprivation index (an area-based measure of socioeconomic status), including an interaction term between Townsend deprivation index and sex. CI, confidence interval; HR, hazard ratio; MR, Mendelian randomisation; OR, odds ratio; RHR, ratio of hazard ratios; ROR, ratio of odds ratios.

In observational analyses, ever compared to never smoking was associated with a higher risk for CVD and its subtypes in both females and males (Figure 1). For CVD, CHD, and MI, the observed association was stronger in females than in males, with female-to-male RHRs of 1.08 (95%CI 1.04, 1.12), 1.09 (95%CI 1.05, 1.14), and 1.25 (95%CI 1.14, 1.36), respectively. No sex differences were found for stroke.

In MR analyses, females and males who ever smoked had a higher risk of CVD, CHD, MI, stroke, and ischaemic stroke than those who never smoked. The direction and pattern of effect sizes across the sexes were similar to those in observational analyses, but the CIs were wider and the female-to-male ratios included unity. There was one exception; the causal effect of ever smoking on SAH was stronger in females as compared to males (females OR 2.49 [95%CI 1.43, 4.36], males OR 0.96 [95%CI 0.47, 1.94], female-to-male ROR 2.61 [95%CI 1.06, 6.42]).

### Smoking continuation

**Figure 2:**
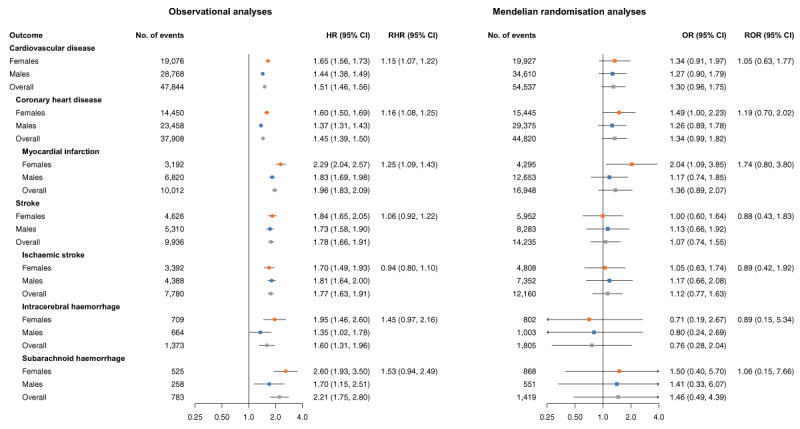
Cox regression and Mendelian randomisation analysis of the association between smoking continuation and risk of cardiovascular events in females, males, and the overall population. MR estimates are from inverse-variance weighted MR, and ORs can be interpreted as the effect per unit increase genetic liability to smoking continuation among ever smokers. MR analyses were performed in 337,386 UK Biobank participants. Cox regressions were performed in 468,838 UK Biobank participants and were adjusted for sex and Townsend deprivation index (an area-based measure of socioeconomic status), including an interaction term between Townsend deprivation index and sex. CI, confidence interval; HR, hazard ratio; MR, Mendelian randomisation; OR, odds ratio; RHR, ratio of hazard ratios; ROR, ratio of odds ratios.

In observational analyses among ever smokers, current smokers were at higher risk for CVD and its subtypes than former smokers (Figure 2). The association of smoking continuation was stronger in females than in males for CVD (RHR 1.15 [95%CI 1.07, 1.22]), CHD (RHR 1.16 [95%CI 1.08, 1.25]), and MI (RHR 1.25 [95%CI 1.09, 1.43]). No sex differences were found in MR analyses.

### Number of cigarettes per day

**Figure 3:**
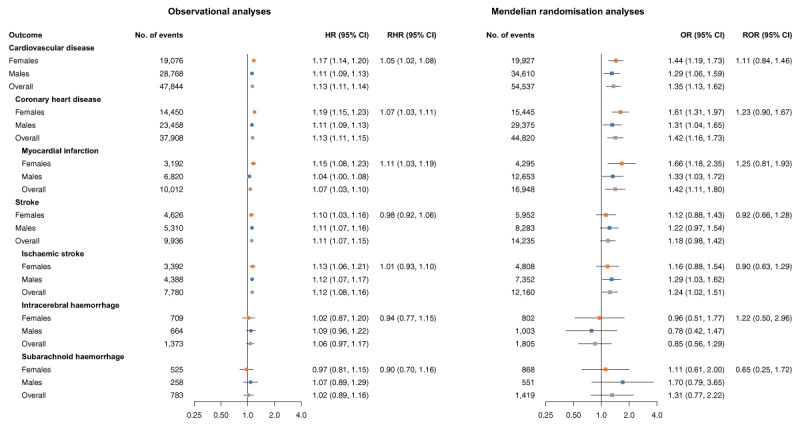
Cox regression and Mendelian randomisation analysis of the association between the number of cigarettes smoked per day and risk of cardiovascular events in females, males, and the overall population. The variable representing the number of cigarettes smoked per day was categorised into the following categories: 1 = 1–5, 2 = 6–15, 3 = 16–25, 4 = 26–35, 5 = 36+ and was analysed as a continuous variable. MR estimates are from inverse-variance weighted MR, and ORs can be interpreted as the effect per category increase of genetically proxied number of cigarettes smoked per day among ever smokers. MR analyses were performed in 337,386 UK Biobank participants. Cox regressions were performed in 468,838 UK Biobank participants and were adjusted for sex and Townsend deprivation index (an area-based measure of socioeconomic status), including an interaction term between Townsend deprivation index and sex. CI, confidence interval; HR, hazard ratio; MR, Mendelian randomisation; OR, odds ratio; RHR, ratio of hazard ratios; ROR, ratio of odds ratios.

In observational analyses among ever smokers, a higher number of cigarettes daily smoked was associated with an increased risk of CVD, CHD, MI, stroke, and ischaemic stroke in females and males (Figure 3). The association of smoking at higher intensities was stronger in females than in males for CVD (RHR 1.05 [95%CI 1.02, 1.08]), CHD (RHR 1.07 [95%CI 1.03, 1.11]), and MI (RHR 1.11 [95%CI 1.03, 1.19]). In MR analyses, a higher number of cigarettes daily smoked was causally associated with CVD (females OR 1.44 [95%CI 1.19, 1.73] and males OR 1.29 [95%CI 1.06, 1.59]) as well as CHD (females OR 1.61 [95%CI 1.31, 1.97] and males OR 1.31 [95%CI 1.04, 1.65]). The pattern of ORs across the sexes in MR was similar to the pattern of HRs in the observational analyses, but no statistically significant sex differences were found in MR analyses.

### Sensitivity analysis

In sensitivity analyses, results were largely robust after adjusting for a set of cardiovascular risk factors (Supplementary Figures S1–S3). Furthermore, the results of observational analyses restricted to the same population as the MR analyses (excluding those with history of CVD at baseline) were directionally similar (Supplementary Figures S4–S6). The results of the MR analyses were broadly similar when applying simple median and weighted median regression, except for the analysis of cigarettes smoked per day where we found larger point estimates using simple median regression and smaller point estimates using weighted median regression (Supplementary Figures S7–S15). The Q test showed evidence of heterogeneity for both females and males and the combined estimates, across smoking phenotypes and CVD outcomes (Supplementary Table S6). We further assessed pleiotropy using MR-Egger regression and found no evidence of directional pleiotropy in the analyses of ever smoking and smoking continuation, except for the analysis of the effect of smoking continuation on SAH in females. However, it is notable that for the relationship between genetic liability to ever smoking and SAH, where inverse-variance weighted regression revealed significant sex differences in causal relationships,, the results were similar when performing simple and weighted median regression, while MR-Egger showed no statistically significant relationship in either females or males (see Figure S7 and Figure S8). For the analysis of the effect of cigarettes smoked per day, we found some evidence for directional pleiotropy. We further investigated this using MR-PRESSO, and outlier-corrected results were similar to the primary analysis (all distortion test *p*-values > 0.05, Supplementary Table S7).

## Discussion

This large-scale study in the UKB assessed the observational and causal associations between smoking and CVD. Ever smoking, smoking continuation, and the number of cigarettes smoked a day were more strongly associated with CVD in females than in males. Sex differences in the causal effects were similar in direction and magnitude, but with a greater degree of uncertainty.

Previous studies have assessed the sex-specific relationship between smoking and CHD. A meta-analysis on 75 cohorts including 2.4 million participants found a pooled adjusted female-to-male ratio of relative risks of CHD of 1.25 (95%CI 1.12, 1.39) of current smoking compared with never smoking (3). These results are in line with results from our observational analyses, where we found a stronger association of smoking and CHD in females than in males across all smoking phenotypes. According to our MR analyses, the sex differences in observed association between smoking and CHD were not likely to be reflective of sex differences in causal effects. However, our MR analyses might have been underpowered to detect statistically significant sex differences.

Sex-specific unmeasured or residual confounding might explain the sex differences in the association between smoking and CVD outcomes in observational studies. If confounding factors are unequally distributed between females and males, or if confounding factors are specific to one sex, a sex-specific bias in the estimated risk factor association could arise. While we adjusted for socioeconomic status (measured as Townsend index in the UKB) and included an interaction term with sex, other unmeasured or residual factors might still be involved. For example, psychosocial stress is a known precursor to smoking and a risk factor for CVD (24,25). Studies have shown that levels of psychosocial stress are higher among females than in males (26). As such, it may contribute to a bias in the association between smoking and CVD, which is stronger in females than in males.

Differential impact of competing risks between females and males could also be involved. Historically, males have smoked at higher intensities and for longer periods than females in most countries (27). As a result, males have a higher risk of dying from other smoking-related diseases before they have had the chance to develop CVD, which could have underestimated the association between smoking and CVD, particularly in males. However, the association between smoking and CVD in females might have been affected by differential misclassification, as females are more likely to underreport their smoking habits (28).

Previous studies have explored sex-specific responses to smoking, examining hormonal, biological, behavioural, and genetic factors (29,30,31,32,33,34). For example, it was found that females may extract a greater quantity of carcinogens and other harmful chemicals from the same number of cigarettes compared to males (35). A recent gene expression study found that the *CRLF1* gene is overexpressed in atherosclerotic plaques of smokers compared to non-smokers, with stronger upregulation in females than in males who smoked (36). Such findings could improve the understanding of the mechanisms underlying the potential sex-specific causal effects of smoking on CVD.

Our observational analyses indicate that smoking confers a similar risk of stroke in both sexes. For all smoking phenotypes, smoking was associated with an increased risk of stroke with a similar magnitude in both sexes. This is consistent with results from a previous meta-analysis of 81 prospective cohort studies, which found a pooled relative risk ratio (females to males) of stroke associated with current smoking of 1.06 (95%CI 0.99, 1.13) (37).

In our MR analyses, no sex differences in the effects of smoking on stroke were found. However, for SAH, which is more common in females than in males, we did find an indication for a sex difference in the effect of ever smoking. A potential explanation for this could be a differential mechanism underlying SAH compared to other CVD outcomes, which are (except for ICH) more related to atherosclerosis. Further research is needed to explore this, potentially including other CVD outcomes such as abdominal aortic aneurysm, to better understand the effects of smoking in non-atherosclerotic CVD outcomes. Although our primary MR analysis based on inverse-variance weighted regression suggested a statistically significant sex difference in the relationship between genetic liability to ever smoking and risk of SAH, the findings need to be interpreted with caution. Only a few individuals (0.2% of females and 0.1% of males) experienced a SAH event within the UKB. Consequently, as demonstrated in Figure 1, CIs for SAH were rather wide for the MR study. Furthermore, the finding is highly driven by a null association in males, and, as we conducted a range of statistical analyses, the sex difference may be significant by chance. Also, MR-Egger reported null associations for both females and males. Moreover, in contrast to our findings, in a previous MR study, the causal effect of smoking initiation on aSAH (aneurysmal SAH) was stronger in males (OR 3.81 [95%CI 1.93, 7.52]) than in females (OR 1.12 [95%CI 0.63, 1.99]) (38). This study used the same GWAS from the GSCAN consortium as we did for selecting instruments. However, it obtained genetic associations with aSAH from a meta-analysis of cohorts with different outcome definitions (39). For example, some cohorts excluded patients with Marfan syndrome, Ehlers–Danlos syndrome, or autosomal dominant polycystic kidney disease. In the current study, we defined SAH in the UKB based on the ICD-10 code I60, which includes any nontraumatic SAH (not limited to aSAH) and did not exclude patients with related conditions. These broader criteria and focus on SAH rather than specifically aSAH may partially explain the contrasting findings. Another potential explanation is a difference in sample overlap. Previous observational evidence on the association between smoking and SAH is inconsistent. A meta-analysis of longitudinal studies on the effect of ever smoking versus never smoking indicated a stronger association between smoking and SAH in females than in males, whereas a meta-analysis of case–control studies on the same association found a stronger association in males (40), and yet another meta-analysis reported a higher risk of persistent smoking in females than in males (41).

### Strengths and limitations

Strengths of this study are the comprehensive approach of sourcing a large sample of UKB participants to study the effect of various smoking phenotypes on several subtypes of CVD through both observational and MR analyses. Our study also has potential limitations. MR relies on three assumptions. First, it is assumed that the selected genetic variants are strongly associated with the risk factor of interest. We used large-scale GWAS data to select our instruments, decreasing the risk of weak instruments. Notably, the GWAS used to obtain genetic associations with the exposures also included data from the UKB, leading to sample overlap. To assess the strength of our instrumental variables, we calculated *F*-statistics. The lowest *F*-statistic was 7.1, indicating a risk of weak instrument bias, which is in the direction of the observed association due to sample overlap (42). The second assumption of MR is the absence of a common cause between genetic variants and the risk factor or outcome. This assumption usually holds in MR because one’s genotype is defined at gametogenesis and is rarely changed by exposure. Population structures, which can affect associations between genetic variants and risk factors or outcomes, were accounted for by restricting our analysis to the European population and adjusting for the first 16 principal components (21). The third assumption is that genetic variants only affect the outcome through the risk factor (i.e., absence of directional pleiotropy), which we investigated by conducting sensitivity analyses. The between-variant heterogeneity indicated by the Q test could be indicative of potential bias due to pleiotropy. However, estimates were broadly similar when using robust methods such as simple and weighted median regression, which make different assumptions with respect to pleiotropy. In addition, the Q test relies on the assumption that all valid instruments identify the same causal parameter, and if this does not hold, it may over-reject the null (43). In addition, the Q test does not give information on the source of the heterogeneity, which could be pleiotropy or other reasons such as confounding by population stratification (44). Therefore, we further assessed pleiotropy using MR-Egger, which indicated potential for directional pleiotropy in the effects of smoking intensity. However, MR-Egger results can be influenced by outlying SNPs (45). To address this, we performed further analysis using MR-PRESSO, which focusses on outliers, and this gave robust results. Another limitation is the unavailability of sex-specific genetic associations with smoking phenotypes, which required us to use sex-combined genetic associations. This will possibly invalidate the MR analysis if there is considerable heterogeneity of the genetic effects between females and males. Bernabeu et al. (46) previously investigated sex differences in the genetic architecture of several traits within the UKB. Among those traits, they also studied current tobacco smoking (regularly, occasionally, no) and smoking status (never, previous, current). They found high genetic correlations between females and males, with 0.9651 (FDR *p*-value for difference from 1: 0.629) for current tobacco smoking and 0.8912 (FDR *p*-value for difference from 1: <0.001) for smoking status. In addition, there were no sex-dimorphic SNPs, i.e., SNPs with a significantly different effect between females and males, for any of these traits. Consequently, it can be hypothesized that using sex-specific genetic instruments may not have had a large impact on our findings. The lower *F*-statistics for females and males compared to the overall UKB population not only increased the risk of weak instrument bias in the sex-specific MR analyses, but also reduced power, which may have contributed to the inability to detect sex differences in risk factor effects, in contrast to the observational analyses, where we were able to detect even minor sex differences. In addition, the smoking phenotypes included in our analyses are expected to be somewhat correlated. However, in individuals of European ancestry, cross-trait genetic correlations between smoking initiation, smoking cessation, and number of cigarettes smoked per day were shown to be moderate, with genetic correlations between 0.30 and 0.44 (15). Furthermore, current GWAS data on smoking intensity categorise the number of cigarettes smoked daily, treating it as a continuous variable, and does not take into account the duration of smoking, limiting the precision of our analysis. GWAS data on pack years, which account for both duration and intensity on a continuous scale, would increase the precision of assessing smoking-intensity-related CVD risk.

## Conclusion

This study shows that both smoking initiation and higher smoking intensity are observationally and causally related to a higher CVD risk in both females and males. Observed sex differences in the association between smoking and CVD were directionally similar to sex differences in the causal effects of smoking on CVD. However, MR estimates were more uncertain and the causal effects of smoking on CVD may be similar in females and males. Future sex-specific GWAS on smoking habits are needed to further assess whether causal effects of smoking on CVD are similar in females and males. This study underscores the importance of smoking reduction for both sexes, emphasising the need to prevent smoking initiation, promote smoking cessation, and decrease smoking intensity in females and males.

## Patient and public involvement

Patients and/or the public were not involved in the design, conduct, reporting, or dissemination plans of this research.

## Data Accessibility Statement

The UK Biobank data used in this study is available upon request via the UKB website (https://www.ukbiobank.ac.uk/enable-your-research/apply-for-access). GWAS summary statistics on smoking phenotypes have been published previously and can be accessed online ((15), https://doi.org/10.13020/przg-dp88).

## Additional File

The additional file for this article can be found as follows:

10.5334/gh.1485.s1 Supplementary Material. Supplemental Methods, Figures and Tables.
