# Supplementary material for: Smoking as a Risk Factor for Cardiovascular Disease in Females and Males: Observational and Mendelian Randomisation Analyses in the UK Biobank
Source: Glob Heart. 2025 Oct 13;20(1):93. doi: 10.5334/gh.1485 (PMC12533459; doi:10.5334/gh.1485)
Supplement: Supplementary Material. — Supplemental Methods, Figures and Tables. [file gh-20-1-1485-s1.pdf]

SUPPLEMENTAL MATERIAL

Supplemental Methods

Genetic data on smoking phenotypes

We used data from a GWAS conducted by the GWAS & Sequencing Consortium of Alcohol and Nicotine use (GSCAN)(15). We included SNPs from the GSCAN GWAS Phase 2(15) and used genetic data from individuals of European ancestry. We used GWAS data that included data from the UK Biobank because analyses were of limited statistical power when relying on the GWAS summary statistics excluding the UK Biobank. This multi-ancestry GWAS meta-analysis included 59 cohorts of individuals of European ancestry, with total sample sizes of 2,669,029 individuals for ever smoking, 1,147,272 for continuation, and 618,489 for the number of cigarettes daily smoked.

Detailed definitions of smoking phenotypes

Ever smoking

Participants were categorized based on responses to current and past tobacco use, using the UKB Data Field 1239 (“Do you smoke tobacco now?”) and Data Field 1249 (“In the past, how often have you smoked tobacco?”). Participants reporting "Yes, on most or all days" in Data Field 1239 or “Smoked on most or all days” in Data Field 1249 were coded as "1" (ever smoker). Participants reporting "Only occasionally" or "No" in Data Field 1239 and "I have never smoked", “Just tried once or twice” or “Smoked occasionally” in Data Field 1249 were coded as "0" (never smoker). Responses of "Prefer not to answer" were considered missing.

Smoking continuation

Participants were categorized based on responses to current and past tobacco use, using Data Field 1239 (“Do you smoke tobacco now?”) and Data Field 1249 (“In the past, how often have you smoked tobacco?”). Current smokers, who reported "Yes, on most or all days" in Data Field 1239, were coded as "1". Former smokers, who reported “Smoked on most or all days” in Data Field 1249 and "Only

occasionally" or "No" in Data Field 1239, were coded as "0". Participants who never smoked or provided "Prefer not to answer" as response in either data field were considered missing.

Cigarettes smoked per day

The number of cigarettes smoked per day was determined using Data Field 3456 (“About how many cigarettes do you smoke on average each day?”) for current smokers, Data Field 6183 (“About how many cigarettes did you smoke on average each day?”) for current cigar/pipe smokers, and Data Field 2887 (“About how many cigarettes did you smoke on average each day?”) for former smokers. Participants without specific smoking data were coded as missing. The cigarette count was categorized into bins as follows: 1=1-5, 2=6-15, 3=16-25, 4=26-35, 5=36+.

Definition of additional variables

Systolic blood pressure (Data Field 4080) was obtained by automated reading and an average over two measures taken a few moments apart was calculated. Intake of antihypertensive medication (Data Fields 6153 and 6177) was self-reported. Total cholesterol (Data Field 30690), high-density lipoprotein cholesterol (Data Field 30760), and C-reactive protein (Data Field 30710) were conducted on the Beckman Coulter AU5800 platform. Body mass index (Data Field 21001) was calculated by weight (in kg) divided by height (in m^2), which were both measured during the initial Assessment Centre visit. Type 2 diabetes mellitus was defined by combining self-reported diagnosis and data derived through linkage with routinely collected data from general practitioners, hospital admissions and death records.

Multiple imputation of missing values

Missing values were imputed using multiple imputation by chained equations with 20 data sets and 30 iterations. The imputation model included the following variables: current tobacco smoking, past tobacco smoking, the number of cigarettes daily smoked in current smokers, age at baseline, total cholesterol, high-density lipoprotein cholesterol, low density lipoprotein cholesterol, triglycerides,

Lipoprotein(a), HbA1c, body mass index, type 2 diabetes status, systolic blood pressure, diastolic blood pressure, Townsend index, C-reactive protein, eGFR, alcohol consumption, the use of lipid lowering medication, the use of antihypertensives, and the Nelson-Aalen estimator for CVD. Predictive mean matching (pmm) was used for continuous variables, logistic regression (logreg) was used for binary variables and polytomous regression (polyreg) was used for categorical variables with multiple levels. If two predictors correlated strongly with each other (Pearson correlation coefficient higher than 0.7 or lower than -0.7), we selected the predictor with the greater correlation to the imputed variable for predicting that variable. We implemented passive imputation for the smoking phenotypes as they were based on other UKB fields of smoking exposure, such as past and current smoking status. For instance, the number of cigarettes smoked per day was only imputed in current and ever smokers. Smoking continuation was only imputed for ever smokers. Imputed results were pooled using Rubin's rule.

Instrumental variable strength

To assess the strength of our instruments, we calculated the F-statistics for each exposure. We used a different approach for binary and continuous or categorical exposures.

For ever smoking and smoking continuation, we conducted logistic regression with smoking status as dependent variable and all instruments as independent variables in the first imputed UKB data set. F-statistics were obtained from a comparison of the full model with a null model containing only an intercept.

In the case of cigarettes per day, we conducted linear regression with the binned number of cigarettes per day as dependent variable and all instruments as independent variables in the first imputed UKB data set to obtain the F-statistic.

Supplementary Tables

Table S1. STROBE checklist

	Item No.	Recommendation	Page No.
Title and abstract	1	(a) Indicate the study's design with a commonly used term in the title or the abstract	1, 2
		(b) Provide in the abstract an informative and balanced summary of what was done and what was found	2
**Introduction**			
Background/rationale	2	Explain the scientific background and rationale for the investigation being reported	3
Objectives	3	State specific objectives, including any prespecified hypotheses	3
**Methods**			
Study design	4	Present key elements of study design early in the paper	4
Setting	5	Describe the setting, locations, and relevant dates, including periods of recruitment, exposure, follow-up, and data collection	4
Participants	6	(a) Give the eligibility criteria, and the sources and methods of selection of participants. Describe methods of follow-up	4
		(b) For matched studies, give matching criteria and number of exposed and unexposed	NA
Variables	7	Clearly define all outcomes, exposures, predictors, potential confounders, and effect modifiers. Give diagnostic criteria, if applicable	4-6
Data sources/ measurement	8*	For each variable of interest, give sources of data and details of methods of assessment (measurement). Describe comparability of assessment methods if there is more than one group	4-6
Bias	9	Describe any efforts to address potential sources of bias	6, Supplemental Methods
Study size	10	Explain how the study size was arrived at	4, 7
Quantitative variables	11	Explain how quantitative variables were handled in the analyses. If applicable, describe which groupings were chosen and why	4
Statistical methods	12	(a) Describe all statistical methods, including those used to control for confounding	6, 7, Supplemental Methods
		(*b*) Describe any methods used to examine subgroups and interactions	
		(*c*) Explain how missing data were addressed	
		(*d*) If applicable, explain how loss to follow-up was addressed	
		(*e*) Describe any sensitivity analyses	
**Results**			

Participants	13*	(a) Report numbers of individuals at each stage of study—eg numbers potentially eligible, examined for eligibility, confirmed eligible, included in the study, completing follow-up, and analysed	7
		(b) Give reasons for non-participation at each stage	
		(c) Consider use of a flow diagram	
Descriptive data	14*	(a) Give characteristics of study participants (eg demographic, clinical, social) and information on exposures and potential confounders	7, 8, Table 1
		(b) Indicate number of participants with missing data for each variable of interest	
		(c) Summarise follow-up time (eg, average and total amount)	
Outcome data	15*	Report numbers of outcome events or summary measures over time	Table 1
Main results	16	(*a*) Give unadjusted estimates and, if applicable, confounder-adjusted estimates and their precision (eg, 95% confidence interval). Make clear which confounders were adjusted for and why they were included	Figure 1 - 3
		(*b*) Report category boundaries when continuous variables were categorized	
		(*c*) If relevant, consider translating estimates of relative risk into absolute risk for a meaningful time period	
Other analyses	17	Report other analyses done—eg analyses of subgroups and interactions, and sensitivity analyses	Figure S1, S2
**Discussion**			
Key results	18	Summarise key results with reference to study objectives	9-11
Limitations	19	Discuss limitations of the study, taking into account sources of potential bias or imprecision. Discuss both direction and magnitude of any potential bias	12
Interpretation	20	Give a cautious overall interpretation of results considering objectives, limitations, multiplicity of analyses, results from similar studies, and other relevant evidence	13, 14
Generalisability	21	Discuss the generalisability (external validity) of the study results	13
**Other information**			
Funding	22	Give the source of funding and the role of the funders for the present study and, if applicable, for the original study on which the present article is based	15

Table S2. STROBE-MR checklist

Item No.	Section		Checklist item	Page No.
1	Title and abstract		Indicate Mendelian randomization (MR) as the study's design in the title and/or the abstract if that is a main purpose of the study	1-2
	**Introduction**			
2	Background		Explain the scientific background and rationale for the reported study. What is the exposure? Is a potential causal relationship between exposure and outcome plausible? Justify why MR is a helpful method to address the study question	3
3	Objectives		State specific objectives clearly, including pre-specified causal hypotheses (if any). State that MR is a method that, under specific assumptions, intends to estimate causal effects	3
	**Methods**			
4	Study design and data sources		Present key elements of the study design early in the article. Consider including a table listing sources of data for all phases of the study. For each data source contributing to the analysis, describe the following:	
		a)	Setting: Describe the study design and the underlying population, if possible. Describe the setting, locations, and relevant dates, including periods of recruitment, exposure, follow-up, and data collection, when available.	4-6, Table S5, Supplementary Methods
		b)	Participants: Give the eligibility criteria, and the sources and methods of selection of participants. Report the sample size, and whether any power or sample size calculations were carried out prior to the main analysis	4
		c)	Describe measurement, quality control and selection of genetic variants	5-7
		d)	For each exposure, outcome, and other relevant variables, describe methods of assessment and diagnostic criteria for diseases	5-7
		e)	Provide details of ethics committee approval and participant informed consent, if relevant	4
5	Assumptions		Explicitly state the three core IV assumptions for the main analysis (relevance, independence and exclusion restriction) as well assumptions for any additional or sensitivity analysis	12
6	Statistical methods: main analysis		Describe statistical methods and statistics used	
		a)	Describe how quantitative variables were handled in the analyses (i.e., scale, units, model)	Table 1, 7
		b)	Describe how genetic variants were handled in the analyses and, if applicable, how their weights were selected	5
		c)	Describe the MR estimator (e.g. two-stage least squares, Wald ratio) and related statistics. Detail the included covariates and, in case of two-sample MR, whether the same covariate set was used for adjustment in the two samples	7
		d)	Explain how missing data were addressed	NA
		**e)**	If applicable, indicate how multiple testing was addressed	NA

7	Assessment of assumptions		Describe any methods or prior knowledge used to assess the assumptions or justify their validity	7
8	Sensitivity analyses and additional analyses		Describe any sensitivity analyses or additional analyses performed (e.g. comparison of effect estimates from different approaches, independent replication, bias analytic techniques, validation of instruments, simulations)	7
9	Software and pre-registration			
		a)	Name statistical software and package(s), including version and settings used	7
		b)	State whether the study protocol and details were pre-registered (as well as when and where)	NA
	**Results**			
10	Descriptive data			
		a)	Report the numbers of individuals at each stage of included studies and reasons for exclusion. Consider use of a flow diagram	8
		b)	Report summary statistics for phenotypic exposure(s), outcome(s), and other relevant variables (e.g. means, SDs, proportions)	Table S5
		c)	If the data sources include meta-analyses of previous studies, provide the assessments of heterogeneity across these studies	Reference of GSCAN at page 5
		d)	For two-sample MR:<br>i. Provide justification of the similarity of the genetic variant-exposure associations between the exposure and outcome samples<br>ii. Provide information on the number of individuals who overlap between the exposure and outcome studies	Supplemental Methods, page 4, and for cohorts used in GSCAN, see reference page 5
11	Main results			
		a)	Report the associations between genetic variant and exposure, and between genetic variant and outcome, preferably on an interpretable scale	Table S3 and data availability for GWAS summary statistics
		b)	Report MR estimates of the relationship between exposure and outcome, and the measures of uncertainty from the MR analysis, on an interpretable scale, such as odds ratio or relative risk per SD difference	Figure 1-3
		c)	If relevant, consider translating estimates of relative risk into absolute risk for a meaningful time period	NA
		d)	Consider plots to visualize results (e.g. forest plot, scatterplot of associations between genetic variants and outcome versus between genetic variants and exposure)	Figure 1-3
12	Assessment of assumptions			
		a)	Report the assessment of the validity of the assumptions	12, Table S6, S7, Supplementary Figures S4-12
		b)	Report any additional statistics (e.g., assessments of heterogeneity across genetic variants, such as I^2, Q statistic or E-value)	NA
13	Sensitivity analyses and additional analyses			

		a)	Report any sensitivity analyses to assess the robustness of the main results to violations of the assumptions	Supplementary Figures S4-12, Table S6, S7
		b)	Report results from other sensitivity analyses or additional analyses	Figures 2-4
		c)	Report any assessment of direction of causal relationship (e.g., bidirectional MR)	NA
		d)	When relevant, report and compare with estimates from non-MR analyses	8-9, Figure 1-3
		e)	Consider additional plots to visualize results (e.g., leave-one-out analyses)	Supplementary Figures S4-12
	**Discussion**			
14	Key results		Summarize key results with reference to study objectives	10
15	Limitations		Discuss limitations of the study, taking into account the validity of the IV assumptions, other sources of potential bias, and imprecision. Discuss both direction and magnitude of any potential bias and any efforts to address them	12
16	Interpretation			
		a)	Meaning: Give a cautious overall interpretation of results in the context of their limitations and in comparison with other studies	10-13
		b)	Mechanism: Discuss underlying biological mechanisms that could drive a potential causal relationship between the investigated exposure and the outcome, and whether the gene-environment equivalence assumption is reasonable. Use causal language carefully, clarifying that IV estimates may provide causal effects only under certain assumptions	11, 12
		c)	Clinical relevance: Discuss whether the results have clinical or public policy relevance, and to what extent they inform effect sizes of possible interventions	13
17	Generalizability		Discuss the generalizability of the study results (a) to other populations, (b) across other exposure periods/timings, and (c) across other levels of exposure	12
	**Other information**			
18	Funding		Describe sources of funding and the role of funders in the present study and, if applicable, sources of funding for the databases and original study or studies on which the present study is based	14
19	Data and data sharing		Provide the data used to perform all analyses or report where and how the data can be accessed, and reference these sources in the article. Provide the statistical code needed to reproduce the results in the article, or report whether the code is publicly accessible and if so, where	14
20	Conflicts of Interest		All authors should declare all potential conflicts of interest	14

Table S3. Overview of selected genetic variants and the associations between genetic variant and outcome.

Supplementary Table S3

rsid	hromosom	Position	Other allel	Effect allel	allele freq	otyping qu	HWEchi2	HWEp
rs1000136	04	1.48E+08	G	A	0.37873	0.9983	3.80e-01	5.38e-01
rs1004787	02	45159091	G	A	0.53578	0.9893	1.39e+00	2.39e-01
rs1006260	05	79290634	C	A	0.63519	0.99231	1.31e+00	2.52e-01
rs1009181	06	26158993	T	C	0.33499	1	3.07e-04	9.86e-01
rs1011911	09	29740028	C	T	0.49702	0.99704	3.17e-01	5.73e-01
rs1012193	09	11175974	T	A	0.50895	0.98893	1.29e+00	2.55e-01
rs1018224	02	22964436	G	A	0.64612	0.99225	1.37e-05	9.97e-01
rs1023301	07	1.18E+08	A	G	0.51292	0.99691	6.95e-06	9.98e-01
rs1025910	18	49871340	G	C	0.29424	0.99626	2.29e-02	8.80e-01
rs1027926	07	1.34E+08	G	A	0.62923	0.99386	4.09e+00	4.31e-02
rs1040227	19	45329214	T	G	0.34493	0.99429	6.26e-01	4.29e-01
rs1044431	11	7951242	T	G	0.42843	0.99965	4.81e-01	4.88e-01
rs1045856	01	50612250	A	G	0.19086	0.99286	6.67e-01	4.14e-01
rs1050847	16	87443734	C	T	0.5507	0.99099	2.57e-01	6.12e-01
rs1069871	06	1.59E+08	G	A	0.05268	0.99429	1.43e-01	7.05e-01
rs1074532	01	1.13E+08	A	G	0.67296	0.99495	2.68e+00	1.02e-01
rs1075363	01	1.64E+08	C	T	0.34891	1	2.25e-01	6.35e-01
rs1077190	15	79071384	T	G	0.19086	0.98258	1.69e+00	1.93e-01
rs1078672	10	1.05E+08	C	A	0.4165	0.99996	2.19e-02	8.82e-01
rs1084445	01	72996521	T	C	0.22565	0.99907	1.07e-01	7.43e-01
rs1092703	01	2.44E+08	T	C	0.19682	0.99691	2.61e+00	1.06e-01
rs1095041	07	1909086	C	G	0.60934	0.99249	8.54e-02	7.70e-01
rs1101272	10	21797272	T	C	0.33002	0.98103	6.56e-01	4.18e-01
rs1107632	16	52097552	C	A	0.63221	0.99151	1.59e+00	2.08e-01
rs1107871	17	7795972	A	G	0.40855	0.99596	7.71e-01	3.80e-01
rs1110366	09	1.38E+08	C	T	0.17893	0.98155	1.71e+00	1.92e-01
rs1111578	12	1.18E+08	G	T	0.15507	1	1.39e-01	7.09e-01
rs1113038	03	53850005	C	T	0.52584	0.99374	4.94e-02	8.24e-01
rs1116201	01	87913176	C	T	0.37972	0.99563	1.03e+00	3.10e-01
rs1116297	01	80822282	A	C	0.30616	0.99708	1.38e+00	2.40e-01
rs1118617	06	50889019	G	A	0.12425	0.99844	1.36e-03	9.71e-01
rs1119216	10	1.06E+08	G	C	0.18588	0.94637	1.92e+00	1.66e-01
rs1121022	01	73860028	A	G	0.56461	1	3.57e-01	5.50e-01
rs1121780	17	27564013	C	T	0.18191	0.97646	1.25e+01	4.10e-04
rs1125841	10	13533053	C	T	0.33598	0.99945	3.13e-01	5.76e-01
rs1126757	19	55879872	C	T	0.47217	0.99245	1.08e-01	7.42e-01
rs1133824	09	1.36E+08	C	A	0.09046	0.99367	4.07e-02	8.40e-01
rs1140501	03	1.51E+08	T	A	0.06163	1	1.81e+00	1.79e-01
rs1149001	16	72629056	C	G	0.05865	0.99796	6.45e-01	4.22e-01
rs1155641	15	97502995	G	A	0.334	0.99415	8.52e-03	9.26e-01
rs1162659	14	1.05E+08	C	T	0.15606	0.98469	1.74e+00	1.87e-01
rs1163243	15	80987012	A	G	0.44831	0.99638	6.69e-02	7.96e-01
rs1165615	17	44068492	A	G	0.2008	0.97961	3.06e-02	8.61e-01
rs1166334	18	62152458	A	T	0.59244	0.99529	1.18e+00	2.77e-01
rs1167345	19	33928001	T	C	0.11332	0.98842	1.22e-01	7.27e-01
rs1168689	02	1.48E+08	T	C	0.28628	0.99557	1.00e+01	1.55e-03

rs1169370	02	1.63E+08	T	A	0.4165	0.99594	2.35e-01	6.28e-01
rs1169519	02	97711421	G	A	0.11531	0.94691	6.50e-01	4.20e-01
rs1169766	20	61992005	C	T	0.80417	0.97728	5.88e+00	1.53e-02
rs1171670	03	34728753	A	G	0.2674	0.99248	1.69e+00	1.93e-01
rs1172070	03	71060640	C	T	0.50696	1	1.94e+00	1.64e-01
rs1172561	04	67053769	T	C	0.31312	0.94868	2.54e+00	1.11e-01
rs1174262	05	91396113	T	C	0.11531	0.99319	1.19e-04	9.91e-01
rs1175649	06	1E+08	T	A	0.12922	0.99034	1.16e-02	9.14e-01
rs1186512	16	5840465	T	A	0.27336	0.97438	2.72e-01	6.02e-01
rs1187316	18	42659922	C	T	0.13121	0.99753	4.93e-01	4.83e-01
rs1192623	03	55936417	A	G	0.04672	0.98296	1.50e+00	2.21e-01
rs1192855	03	48739883	T	C	0.65109	0.98072	3.44e+00	6.38e-02
rs1194043	04	99509453	T	A	0.65408	0.9736	4.17e+00	4.10e-02
rs1199133	08	9288464	G	A	0.17097	0.99798	1.33e+00	2.49e-01
rs1202799	01	1.54E+08	T	C	0.15109	0.999	1.72e-02	8.96e-01
rs1203605	01	96917491	T	C	0.28429	0.99965	4.73e+00	2.96e-02
rs1207906	01	1.74E+08	A	G	0.44732	0.99745	2.70e-01	6.03e-01
rs1211263	07	69735251	A	G	0.23459	0.99601	1.17e-02	9.14e-01
rs1213306	01	91214714	C	A	0.37177	0.99288	2.29e-01	6.33e-01
rs1220977	06	37372370	C	G	0.53082	0.99209	2.48e-01	6.18e-01
rs1244190	15	83922387	C	A	0.167	0.99909	8.58e-03	9.26e-01
rs1246265	09	86761745	T	C	0.70179	0.99274	3.41e-02	8.53e-01
rs1248539	03	1.81E+08	C	A	0.12326	0.9775	4.11e-02	8.39e-01
rs1260046	17	50306926	A	T	0.30119	0.99232	3.22e-01	5.71e-01
rs1263211	03	50224225	A	G	0.66302	0.99285	1.38e+00	2.40e-01
rs1264274	04	28027176	G	T	0.75746	0.99971	6.82e-01	4.09e-01
rs1266060	06	97970749	T	C	0.04374	0.99084	4.04e-02	8.41e-01
rs1268581	09	8290929	G	A	0.1501	0.99342	1.61e-04	9.90e-01
rs1271401	02	80999398	T	C	0.5328	0.9917	6.95e-02	7.92e-01
rs1274244	01	33877057	G	T	0.63022	0.99285	2.39e-01	6.25e-01
rs1276090	01	1.91E+08	C	T	0.29722	0.99611	1.28e-01	7.21e-01
rs1291865	10	11082192	G	T	0.53181	0.98787	2.23e+00	1.35e-01
rs1292342	16	17575065	C	T	0.21968	0.97962	7.46e+00	6.32e-03
rs1294540	17	80004094	T	C	0.6163	0.98658	2.67e+00	1.02e-01
rs1300900	02	1.74E+08	A	G	0.65109	0.99881	3.42e+00	6.45e-02
rs1303099	02	1.46E+08	G	A	0.50895	0.99602	4.95e-01	4.82e-01
rs1314572	04	1.41E+08	G	C	0.36084	0.99714	4.16e-01	5.19e-01
rs1316230	05	12122698	A	T	0.33499	0.98651	3.39e+00	6.55e-02
rs1317286	15	78896129	A	G	0.37177	1	2.16e-02	8.83e-01
rs1324656	07	3438243	C	G	0.5507	0.99495	3.14e-02	8.59e-01
rs1325457	08	42545846	G	C	0.77734	0.99984	5.64e-01	4.53e-01
rs1325562	08	1.44E+08	A	G	0.69583	0.98066	1.60e-02	8.99e-01
rs1326172	08	91866297	G	C	0.26143	0.99438	1.16e-02	9.14e-01
rs1330107	09	1.28E+08	G	A	0.34891	0.99934	9.72e-02	7.55e-01
rs1334557	10	8790819	C	T	0.24553	0.98649	1.97e-01	6.57e-01
rs1337716	10	63675271	T	A	0.49105	0.99211	2.43e+00	1.19e-01
rs1339841	02	2.04E+08	T	A	0.25944	0.99691	2.60e-01	6.10e-01

rs134529	22	28781758	T	C	0.37972	0.99736	5.43e-01	4.61e-01
rs1381287	14	98597552	C	T	0.50497	0.98644	1.27e-04	9.91e-01
rs1392446	05	30831387	C	T	0.5328	0.99521	2.70e+00	1.00e-01
rs1435679	15	36399245	G	A	0.58151	0.99971	7.30e+00	6.89e-03
rs1439098	02	59294197	T	C	0.10736	0.9908	9.01e-01	3.42e-01
rs1444026	08	1.38E+08	T	G	0.44632	0.9964	1.67e+00	1.96e-01
rs1451045	21	40644110	C	T	0.12525	0.9902	1.46e-02	9.04e-01
rs1454838	03	89344540	T	G	0.43936	0.99615	3.45e-01	5.57e-01
rs1466281	06	67551428	T	A	0.37972	0.97201	2.03e+00	1.54e-01
rs1470521	01	1.8E+08	G	T	0.01491	1	1.31e-03	9.71e-01
rs1485272	03	3727589	T	C	0.37674	0.99265	4.26e-01	5.14e-01
rs1490683	04	1.55E+08	T	C	0.334	0.98185	1.10e-01	7.40e-01
rs1499982	03	1.18E+08	C	T	0.83598	0.98271	4.54e-02	8.31e-01
rs1503211	04	94079508	G	A	0.45427	0.99572	3.57e+00	5.87e-02
rs1565735	08	27426077	T	A	0.1998	0.97996	3.42e-01	5.59e-01
rs160631	06	52895230	T	G	0.72863	0.99636	1.47e+00	2.25e-01
rs1657936	15	57113013	C	T	0.78131	0.99859	2.51e-01	6.16e-01
rs1689619	06	65787895	A	T	0.167	0.98202	9.49e-02	7.58e-01
rs1697517	18	39269650	C	A	0.10835	0.98905	5.07e-01	4.77e-01
rs1719711	11	46520302	T	C	0.08449	0.99912	2.31e-05	9.96e-01
rs172032	19	18633755	T	C	0.42545	0.99396	4.12e-01	5.21e-01
rs1733756	10	56700221	A	G	0.49503	0.99657	3.50e+00	6.14e-02
rs1737894	20	31054702	C	G	0.37773	0.98832	2.33e+00	1.27e-01
rs1743277	02	63466463	C	T	0.22465	0.99734	8.98e-01	3.43e-01
rs1759456	14	79618750	A	G	0.4165	0.98093	5.41e-01	4.62e-01
rs1776631	10	31422577	T	C	0.77435	0.99388	2.45e-01	6.20e-01
rs1899689	07	1.22E+08	C	T	0.37972	0.99873	2.61e-01	6.09e-01
rs1924775	20	19659362	C	T	0.50099	0.98491	4.79e+00	2.87e-02
rs1937443	01	66469643	C	G	0.56859	0.9874	5.77e+00	1.63e-02
rs1945737	18	53744545	T	C	0.51491	0.97359	7.77e+00	5.30e-03
rs1971318	12	1.21E+08	C	T	0.12922	0.99216	3.48e-02	8.52e-01
rs1994247	02	1.56E+08	G	T	0.51491	0.99878	1.83e-06	9.99e-01
rs2006281	14	1.04E+08	C	T	0.53479	0.98481	6.32e-02	8.02e-01
rs2016968	16	89772906	C	G	0.57157	0.98517	2.99e-01	5.85e-01
rs2022815	14	28356733	G	A	0.33598	0.99668	3.51e-01	5.54e-01
rs2060220	05	1.67E+08	A	T	0.1332	0.99094	5.46e-01	4.60e-01
rs2072155	07	77762457	T	C	0.6998	0.99592	4.59e-02	8.30e-01
rs2118362	11	16373083	T	C	0.28628	0.99178	1.54e+00	2.15e-01
rs2133203	01	77974530	C	T	0.4503	0.99562	3.54e-01	5.52e-01
rs2135160	02	2.13E+08	T	C	0.09145	0.98619	9.26e-03	9.23e-01
rs215600	07	32333642	G	A	0.66799	0.99779	6.03e+00	1.41e-02
rs2163413	02	2.26E+08	A	G	0.17594	0.99425	2.25e-01	6.35e-01
rs2173019	05	1.68E+08	T	A	0.17097	0.98438	3.54e+00	5.99e-02
rs2237303	07	21483605	G	A	0.65606	0.98831	4.11e+00	4.27e-02
rs2273500	20	61986949	T	C	0.14612	0.99683	2.03e-01	6.52e-01
rs2289791	15	67476952	G	T	0.24056	0.98215	7.19e-02	7.89e-01
rs2292239	12	56482180	T	G	0.667	1	6.18e-03	9.37e-01

rs2313500	05	1.55E+08	C	T	0.24453	0.99751	3.71e-01	5.43e-01
rs2402821	07	1.26E+08	G	A	0.62326	0.99639	7.48e-02	7.84e-01
rs2655008	03	1.36E+08	A	T	0.73062	0.99774	5.06e-03	9.43e-01
rs2678903	02	58137930	A	G	0.59145	0.99952	2.32e-02	8.79e-01
rs2708630	01	8447404	C	T	0.69185	0.99865	4.56e-02	8.31e-01
rs2711607	15	54117442	G	T	0.15706	0.99178	4.05e-01	5.24e-01
rs2783130	13	80170160	A	G	0.50298	1	8.57e-01	3.55e-01
rs2876586	06	1.45E+08	G	A	0.40756	0.99631	3.16e-01	5.74e-01
rs288181	05	1.07E+08	C	T	0.3171	0.99313	8.18e-01	3.66e-01
rs2939756	11	41436297	G	A	0.48907	0.9875	1.55e-01	6.94e-01
rs3025383	09	1.37E+08	T	C	0.18092	0.98491	7.85e-01	3.76e-01
rs3110590	18	27808203	C	A	0.24851	0.99564	8.34e-01	3.61e-01
rs3213876	18	73183978	T	C	0.31511	0.99514	4.73e-02	8.28e-01
rs326341	03	1.08E+08	G	A	0.46223	0.99054	1.04e+00	3.07e-01
rs332827	01	61743160	G	A	0.45229	0.99131	2.97e-03	9.57e-01
rs3436705[illegible]	02	1.38E+08	C	T	0.17097	0.98935	1.04e+00	3.08e-01
rs3437069[illegible]	01	1.67E+08	C	T	0.17594	0.99225	2.60e+00	1.07e-01
rs3440623[illegible]	19	41305530	C	A	0.02584	1	2.41e+00	1.20e-01
rs3448867[illegible]	15	47684936	T	C	0.20676	0.9923	9.91e-03	9.21e-01
rs3463847[illegible]	03	1.46E+08	G	A	0.36084	0.99387	1.64e-01	6.86e-01
rs3503310[illegible]	20	61726373	T	C	0.04175	0.96171	1.38e+00	2.41e-01
rs3589196[illegible]	11	20129311	G	A	0.07157	1	3.83e-01	5.36e-01
rs3781295	10	1.04E+08	G	A	0.38767	0.99633	4.40e-01	5.07e-01
rs3796462	04	96108829	C	T	0.70179	0.9951	3.49e-01	5.54e-01
rs3801289	07	96638267	A	C	0.36382	0.99267	6.92e-02	7.93e-01
rs3814994	07	88427385	G	T	0.37376	0.996	1.35e+00	2.46e-01
rs3895907	01	75006027	A	G	0.54871	0.99778	4.07e-01	5.24e-01
rs3905125	01	2.37E+08	C	T	0.54274	0.99002	2.22e+00	1.36e-01
rs3934797	04	1.12E+08	G	A	0.17197	0.9935	1.01e+00	3.16e-01
rs39784	05	1.71E+08	C	A	0.72266	0.98793	1.35e-01	7.14e-01
rs404263	12	1.26E+08	C	T	0.65507	0.99526	1.31e-01	7.17e-01
rs4044321	05	1.67E+08	A	G	0.66899	0.99158	2.94e-01	5.88e-01
rs4374330	02	1.82E+08	C	T	0.76441	0.99568	2.62e+00	1.05e-01
rs4401691	06	1.58E+08	C	G	0.53181	0.99865	6.99e-02	7.91e-01
rs4479577	03	5723818	C	T	0.49801	0.98946	6.76e+00	9.35e-03
rs4543592	09	3014254	T	C	0.49006	0.99174	5.73e+00	1.66e-02
rs4571506	05	87756918	C	T	0.48111	0.99448	1.42e-01	7.07e-01
rs4579569	08	64913762	G	A	0.49901	0.99689	1.17e+00	2.79e-01
rs465646	06	1.12E+08	G	A	0.8499	0.99349	2.41e-01	6.24e-01
rs4659805	01	2.38E+08	G	T	0.60537	0.98792	1.30e+00	2.55e-01
rs4705014	05	1.56E+08	G	A	0.66103	1	2.70e-01	6.03e-01
rs4751614	10	1.19E+08	A	T	0.23161	0.98607	2.54e-01	6.14e-01
rs4785187	16	49766772	G	A	0.24453	0.99472	1.39e-01	7.09e-01
rs4819027	21	46495224	C	G	0.71869	0.99227	5.38e-01	4.63e-01
rs4837631	09	1.22E+08	C	T	0.45726	0.99771	5.41e+00	2.00e-02
rs4857114	03	94213083	C	T	0.40656	0.99546	2.53e-01	6.15e-01
rs4888444	16	75690279	A	G	0.03976	1	3.50e-01	5.54e-01

rs4944844	11	73311705	T	C	0.84493	0.99227	8.67e-01	3.52e-01
rs540356	11	1.32E+08	C	A	0.37078	0.97763	1.84e-01	6.68e-01
rs540860	11	1.22E+08	A	G	0.56262	0.99122	5.73e+00	1.67e-02
rs551739	12	40414075	A	T	0.69483	0.99497	2.17e+00	1.41e-01
rs557544	06	1.09E+08	T	C	0.35686	0.95359	6.07e+00	1.37e-02
rs5592113(	01	2.1E+08	T	C	0.24056	0.99209	2.27e-02	8.80e-01
rs5594231	16	49626772	G	A	0.07952	0.98495	4.40e-01	5.07e-01
rs5604960	03	49585243	C	G	0.20278	1	1.05e+00	3.05e-01
rs5611385(	19	41353107	T	C	0.59244	0.99388	6.80e+00	9.10e-03
rs5616960	01	18439991	G	A	0.44135	0.97807	4.25e-01	5.14e-01
rs5622537	16	13755408	T	G	0.15805	0.99325	1.11e-02	9.16e-01
rs5634859	09	16749265	A	G	0.14215	0.99098	2.07e+00	1.50e-01
rs5682092	20	54387374	C	T	0.35487	0.99782	1.23e-02	9.12e-01
rs574835	11	64110668	G	A	0.3658	0.99582	1.17e-02	9.14e-01
rs5751239	22	42592239	C	T	0.52783	0.99016	1.75e+00	1.86e-01
rs5840086	04	31184484	G	A	0.38569	0.98876	1.95e+00	1.62e-01
rs5854718	19	18462708	G	A	0.24553	0.97706	8.41e-05	9.93e-01
rs591143	15	47647755	C	T	0.62923	0.9976	8.71e-01	3.51e-01
rs6045392	10	87362579	T	A	0.1501	0.99429	2.08e+00	1.49e-01
rs6074956	08	42602668	A	T	0.08052	0.97917	1.86e+00	1.73e-01
rs6078372	20	11860291	G	A	0.42048	1	3.35e-01	5.63e-01
rs6088618	20	33409350	G	A	0.4334	0.9982	1.72e-01	6.78e-01
rs6141314	20	31093514	G	A	0.21571	0.98678	8.78e-01	3.49e-01
rs6153374	02	22582968	T	C	0.37773	0.99251	6.85e-01	4.08e-01
rs6210726	02	422144	T	C	0.04473	0.99281	6.23e-01	4.30e-01
rs6213552	02	44299879	C	T	0.03877	0.98897	2.32e-03	9.62e-01
rs6218177	02	2.01E+08	G	A	0.55964	0.99216	6.70e-02	7.96e-01
rs6225071	03	85513793	A	G	0.6332	0.99757	2.07e-02	8.85e-01
rs6225417	03	84624766	G	A	0.01491	0.92355	3.06e+00	8.02e-02
rs6225890	03	75102433	C	G	0.12724	0.98078	1.17e+00	2.80e-01
rs6226687(	03	1.29E+08	C	G	0.07256	1	5.23e+00	2.22e-02
rs6241957	06	84356959	T	A	0.12227	0.98908	5.64e-03	9.40e-01
rs6265	11	27679916	C	T	0.19682	1	1.60e-02	8.99e-01
rs6438208	03	1.14E+08	G	A	0.24851	0.92147	5.52e+00	1.88e-02
rs6464024	07	1688369	C	T	0.43738	0.9967	5.85e-01	4.44e-01
rs6472232	08	66792632	T	G	0.37575	0.99245	3.90e-03	9.50e-01
rs6497840	16	25351633	G	A	0.71372	0.99882	2.34e-01	6.28e-01
rs6598539	15	99204483	T	C	0.51292	0.99706	1.02e+00	3.13e-01
rs6603895	01	18751483	T	A	0.51392	0.99982	7.84e-01	3.76e-01
rs6648087(	14	32448189	C	G	0.44235	0.99673	4.24e+00	3.95e-02
rs6668080(	03	85985324	G	T	0.43042	0.99979	3.06e-02	8.61e-01
rs669696	16	69626136	C	A	0.42346	0.99743	1.06e-01	7.45e-01
rs6699355	01	35384605	C	T	0.87773	0.93932	1.34e+00	2.47e-01
rs6705147	02	1.33E+08	C	T	0.32207	1	1.89e+00	1.69e-01
rs6728726	02	623976	T	C	0.83002	0.99982	8.67e-01	3.52e-01
rs6816088	04	35563391	T	C	0.15308	0.98579	3.66e-01	5.45e-01
rs6831786	04	67875548	C	A	0.55865	0.99673	1.36e+00	2.44e-01

rs6852117	04	1.73E+08	C	G	0.40159	0.99747	1.91e-01	6.62e-01
rs6861333	05	50703933	C	G	0.35785	0.9967	4.21e-01	5.17e-01
rs6868892	05	22193780	C	T	0.50497	0.99946	1.32e+00	2.51e-01
rs6959670	07	1.11E+08	C	T	0.35586	0.99631	1.08e+00	2.98e-01
rs6973168	07	1.15E+08	C	G	0.59046	0.9912	1.72e-02	8.96e-01
rs7092291	10	10040004	C	T	0.41252	0.99451	2.24e-01	6.36e-01
rs7127712	11	1.13E+08	A	T	0.43439	0.99908	1.40e-01	7.08e-01
rs71367545	18	77576337	G	A	0.16103	0.9958	2.30e+00	1.29e-01
rs71491832	11	1.25E+08	C	G	0.07455	0.99125	1.07e+01	1.04e-03
rs71563618	07	1.15E+08	A	G	0.08449	0.97872	1.12e-01	7.38e-01
rs71627577	05	43125795	A	G	0.11928	0.96018	1.20e+00	2.73e-01
rs7195043	16	90020861	C	T	0.44433	0.96125	1.61e+01	6.04e-05
rs7205551	16	69605968	G	A	0.43439	0.99704	2.11e+00	1.46e-01
rs7224742	17	30657058	C	T	0.6332	0.99629	1.73e+00	1.88e-01
rs72664906	01	32170141	T	C	0.11928	0.98921	4.64e+00	3.12e-02
rs72733235	09	38275772	T	C	0.17793	0.99794	1.08e-01	7.42e-01
rs72740955	15	78849779	C	T	0.37376	0.9971	5.78e-02	8.10e-01
rs72781639	02	24204148	C	G	0.16004	0.99969	1.50e-01	6.99e-01
rs72789627	05	1.07E+08	C	T	0.1342	0.98571	5.47e-01	4.60e-01
rs72904370	11	46040015	G	C	0.21769	0.99804	2.08e+00	1.49e-01
rs72976960	19	4029784	A	G	0.18489	0.998	3.19e+00	7.41e-02
rs73229090	08	27442127	C	A	0.11332	0.97546	9.07e-02	7.63e-01
rs7333559	13	1.01E+08	G	A	0.79125	0.98244	2.21e-01	6.39e-01
rs745570	17	77781725	A	G	0.49503	0.99906	4.27e-01	5.13e-01
rs7585579	02	60024857	C	G	0.5169	0.97405	3.83e-01	5.36e-01
rs7599488	02	60718347	C	T	0.4175	1	3.54e-01	5.52e-01
rs76132272	01	2.27E+08	C	T	0.05567	0.99992	3.73e-05	9.95e-01
rs7629352	03	16848835	A	G	0.29622	0.99702	6.25e-01	4.29e-01
rs763053	16	735921	T	C	0.2674	0.99138	1.69e+00	1.93e-01
rs76608582	19	4474725	C	A	0.04573	0.90501	1.83e-01	6.68e-01
rs7666804	04	57750148	T	C	0.39364	0.98979	6.31e-01	4.27e-01
rs7678019	04	2997194	G	A	0.37873	0.99922	1.40e-01	7.08e-01
rs77307359	04	34839767	T	C	0.21173	0.99717	2.05e-01	6.50e-01
rs7788527	07	70579487	T	C	0.68688	0.99545	2.66e+00	1.03e-01
rs7802341	07	74324953	A	T	0.74652	0.91799	1.23e+01	4.59e-04
rs7804551	07	99119110	A	G	0.16302	0.99948	1.95e-03	9.65e-01
rs7807019	07	1.18E+08	A	G	0.47018	0.99727	4.40e-02	8.34e-01
rs78175438	21	40663255	T	C	0.11829	1	1.86e-01	6.66e-01
rs7829715	08	59803836	T	C	0.56064	0.99299	2.71e-02	8.69e-01
rs7830359	08	10834258	C	T	0.3509	0.96679	8.38e-02	7.72e-01
rs79222572	06	1.65E+08	T	G	0.25547	0.99721	7.77e+00	5.32e-03
rs79250609	12	1.1E+08	C	T	0.17694	0.95269	1.39e+00	2.38e-01
rs7928017	11	1.13E+08	C	A	0.40756	0.99273	3.20e-01	5.72e-01
rs7929518	11	85980958	A	G	0.77237	0.99926	1.01e+00	3.14e-01
rs7933830	11	16377119	C	T	0.31909	0.9831	2.89e+00	8.89e-02
rs7944241	11	43695374	A	G	0.40954	0.99419	9.53e-02	7.57e-01
rs7947391	11	66186882	A	G	0.57754	0.99214	2.74e-02	8.68e-01

rs7969559	12	69655167	A	G	0.68191	0.99729	4.73e-01	4.91e-01
rs7984311	13	1.01E+08	G	A	0.38767	0.99069	6.47e-01	4.21e-01
rs7986094	13	31029931	A	C	0.69483	0.98992	5.84e-01	4.45e-01
rs8001839	13	97110073	A	G	0.32306	0.99855	3.23e+00	7.23e-02
rs8021229	14	1.04E+08	C	T	0.29622	0.99954	2.42e+00	1.20e-01
rs8029210[illegible]	06	26237068	A	G	0.29523	0.98632	9.90e-02	7.53e-01
rs806255	08	64619016	G	A	0.7326	0.99364	5.12e-01	4.74e-01
rs8067305	17	1975657	G	A	0.57455	0.99031	2.23e+00	1.35e-01
rs8069451	17	37504933	T	C	0.24652	0.98934	1.86e-01	6.66e-01
rs846781	06	1.01E+08	T	C	0.73758	0.99818	8.50e+00	3.54e-03
rs853684	06	28294550	T	C	0.38072	1	1.35e+00	2.45e-01
rs888292	18	25210972	A	T	0.29523	0.98376	4.64e-01	4.96e-01
rs911781	10	1.24E+08	A	G	0.56859	0.99716	8.00e-02	7.77e-01
rs9323328	14	58653514	A	G	0.52684	0.99512	1.53e-01	6.96e-01
rs9375371	06	98751680	G	A	0.29324	0.99404	1.06e-02	9.18e-01
rs9402093	06	1.29E+08	G	T	0.69185	0.99801	1.35e-01	7.13e-01
rs9423279	10	1.26E+08	C	G	0.63817	0.95876	1.69e+00	1.93e-01
rs951740	01	44011737	G	A	0.63618	0.99476	7.10e-01	3.99e-01
rs9522262	13	1.12E+08	C	G	0.51988	0.98922	7.10e-01	4.00e-01
rs9529052	13	66940097	T	A	0.50696	0.99694	5.18e-01	4.72e-01
rs9538536	13	60536321	T	G	0.66899	0.99656	2.69e-01	6.04e-01
rs9541499	13	69261072	T	G	0.0825	0.94676	2.07e+00	1.50e-01
rs9607805	22	41854446	C	T	0.70278	0.99136	6.33e-01	4.26e-01
rs9613472	22	27972479	A	G	0.54175	0.98829	4.08e+00	4.33e-02
rs9627272	22	46442288	G	C	0.38966	0.98319	6.95e-01	4.04e-01
rs963354	03	1.57E+08	C	A	0.70477	0.98999	1.37e+00	2.42e-01
rs9646638	19	41473136	A	G	0.01093	0.93936	8.22e-03	9.28e-01
rs9679319	02	1.04E+08	T	G	0.56163	0.99858	1.88e+00	1.70e-01
rs9763225	05	60257450	A	G	0.26839	0.99954	5.59e-02	8.13e-01
rs9881798	03	16846967	A	C	0.39165	0.99466	1.45e+00	2.29e-01
rs993700	04	67825894	T	C	0.7674	0.99879	3.87e-01	5.34e-01
rs9987376	08	93190014	T	G	0.56958	1	1.84e+00	1.75e-01

sure: Smoki	ure: Smoki	ure: Cigare	D female B	VD female S	VD male Be	CVD male S	D overall B	VD overall S
x			-2.21e-03	1.12e-02	1.14e-03	9.16e-03	-1.96e-04	7.08e-03
x			2.03e-02	1.09e-02	7.38e-03	8.96e-03	1.27e-02	6.94e-03
x			5.24e-03	1.12e-02	1.34e-02	9.18e-03	1.01e-02	7.10e-03
	x		-5.73e-05	1.12e-02	-1.83e-02	9.19e-03	-1.11e-02	7.10e-03
x			-2.49e-03	1.09e-02	1.39e-02	8.93e-03	7.52e-03	6.91e-03
x			1.73e-02	1.09e-02	1.31e-02	8.95e-03	1.47e-02	6.92e-03
	x		6.18e-03	1.16e-02	1.30e-02	9.54e-03	1.03e-02	7.37e-03
x			6.29e-03	1.09e-02	7.16e-03	8.93e-03	6.81e-03	6.90e-03
x			-6.25e-03	1.17e-02	-2.24e-02	9.64e-03	-1.57e-02	7.43e-03
x			3.55e-03	1.12e-02	1.44e-02	9.22e-03	9.94e-03	7.13e-03
	x		1.52e-02	1.16e-02	1.64e-02	9.52e-03	1.58e-02	7.37e-03
x			-1.14e-02	1.11e-02	-9.73e-03	9.13e-03	-1.04e-02	7.06e-03
x			-1.99e-03	1.34e-02	7.09e-03	1.10e-02	3.49e-03	8.48e-03
x			4.02e-03	1.10e-02	-5.82e-03	9.05e-03	-1.77e-03	7.00e-03
x			3.26e-02	2.30e-02	-1.30e-02	1.92e-02	5.64e-03	1.48e-02
x			4.20e-03	1.13e-02	-5.82e-03	9.31e-03	-1.68e-03	7.20e-03
x			1.53e-02	1.15e-02	-9.05e-03	9.44e-03	7.68e-04	7.30e-03
		x	2.47e-02	1.40e-02	5.27e-02	1.15e-02	4.15e-02	8.88e-03
x			2.53e-03	1.11e-02	-1.48e-02	9.12e-03	-7.94e-03	7.06e-03
x			-1.34e-02	1.28e-02	-1.50e-02	1.06e-02	-1.44e-02	8.15e-03
x			-1.68e-02	1.40e-02	1.21e-02	1.14e-02	5.62e-04	8.86e-03
x			-2.37e-02	1.11e-02	1.58e-03	9.11e-03	-8.52e-03	7.04e-03
x			2.47e-02	1.18e-02	1.89e-02	9.74e-03	2.13e-02	7.52e-03
		x	-1.99e-02	1.11e-02	-8.15e-03	9.15e-03	-1.29e-02	7.08e-03
x			1.39e-02	1.10e-02	4.88e-03	9.06e-03	8.66e-03	7.00e-03
x			3.03e-02	1.38e-02	-6.19e-03	1.14e-02	8.42e-03	8.79e-03
x			-1.21e-03	1.47e-02	-2.31e-02	1.21e-02	-1.40e-02	9.37e-03
x			-1.47e-02	1.09e-02	-3.88e-03	8.97e-03	-8.12e-03	6.93e-03
x			-1.57e-02	1.14e-02	-2.99e-03	9.28e-03	-8.11e-03	7.19e-03
x			6.26e-03	1.18e-02	3.17e-03	9.69e-03	4.49e-03	7.50e-03
x			-2.84e-02	1.81e-02	-4.90e-02	1.48e-02	-4.08e-02	1.15e-02
x			5.81e-03	1.40e-02	2.84e-02	1.15e-02	1.94e-02	8.90e-03
x			-1.95e-02	1.12e-02	-2.05e-02	9.16e-03	-2.03e-02	7.09e-03
		x	1.05e-02	1.47e-02	3.20e-02	1.20e-02	2.33e-02	9.31e-03
x			-1.24e-02	1.11e-02	-2.18e-02	9.07e-03	-1.80e-02	7.03e-03
x			-1.03e-03	1.09e-02	-1.29e-04	8.95e-03	-5.52e-04	6.92e-03
	x		5.03e-02	1.70e-02	2.23e-02	1.41e-02	3.37e-02	1.09e-02
x			-3.90e-04	2.27e-02	-9.68e-04	1.88e-02	-5.07e-04	1.45e-02
x			-1.23e-02	2.08e-02	-1.73e-02	1.69e-02	-1.55e-02	1.31e-02
x			1.17e-02	1.14e-02	9.11e-03	9.36e-03	1.03e-02	7.24e-03
x			3.29e-03	1.45e-02	3.83e-03	1.18e-02	3.58e-03	9.18e-03
x			3.00e-02	1.09e-02	2.30e-02	8.92e-03	2.57e-02	6.91e-03
x			-5.32e-03	1.29e-02	-2.32e-02	1.06e-02	-1.59e-02	8.17e-03
		x	-6.29e-03	1.10e-02	1.16e-02	9.05e-03	4.37e-03	6.99e-03
x			-3.48e-05	1.71e-02	8.01e-03	1.40e-02	4.95e-03	1.08e-02
		x	-2.74e-02	1.16e-02	-1.44e-02	9.54e-03	-1.96e-02	7.38e-03

x			3.93e-03	1.09e-02	3.01e-04	8.95e-03	1.75e-03	6.92e-03
x			-1.94e-02	1.78e-02	2.69e-02	1.45e-02	8.17e-03	1.12e-02
	x		-3.27e-02	1.41e-02	-1.93e-02	1.16e-02	-2.48e-02	8.96e-03
x			3.68e-03	1.25e-02	-5.95e-03	1.03e-02	-2.24e-03	7.96e-03
x			-1.10e-03	1.08e-02	-1.66e-02	8.91e-03	-1.04e-02	6.88e-03
		x	3.46e-02	1.25e-02	1.38e-02	1.03e-02	2.23e-02	7.97e-03
x			2.70e-03	1.71e-02	1.39e-03	1.41e-02	2.21e-03	1.09e-02
x			-3.65e-02	1.63e-02	-2.88e-02	1.33e-02	-3.18e-02	1.03e-02
x			1.17e-02	1.24e-02	1.76e-02	1.02e-02	1.52e-02	7.87e-03
x			-1.77e-02	1.49e-02	-5.86e-03	1.21e-02	-1.09e-02	9.41e-03
x			-4.13e-02	2.44e-02	4.13e-02	1.97e-02	7.90e-03	1.53e-02
		x	-2.31e-02	1.14e-02	-7.52e-03	9.38e-03	-1.39e-02	7.24e-03
		x	-1.92e-02	1.16e-02	-5.23e-04	9.57e-03	-7.89e-03	7.40e-03
	x		-9.73e-03	1.43e-02	2.96e-02	1.17e-02	1.40e-02	9.05e-03
x			-8.49e-03	1.64e-02	1.72e-02	1.34e-02	6.76e-03	1.04e-02
x			-7.19e-03	1.20e-02	4.58e-03	9.84e-03	-9.55e-05	7.61e-03
x			5.35e-03	1.09e-02	6.67e-03	8.96e-03	6.17e-03	6.94e-03
x			2.40e-02	1.23e-02	6.28e-03	1.01e-02	1.36e-02	7.82e-03
x			1.84e-02	1.14e-02	1.38e-02	9.38e-03	1.56e-02	7.25e-03
x			-1.25e-02	1.09e-02	9.08e-04	8.96e-03	-4.31e-03	6.93e-03
x			1.26e-02	1.38e-02	4.43e-03	1.14e-02	7.77e-03	8.78e-03
x			1.91e-02	1.19e-02	-2.83e-03	9.69e-03	6.07e-03	7.50e-03
x			-2.51e-02	1.69e-02	2.06e-02	1.37e-02	2.49e-03	1.06e-02
x			3.29e-02	1.15e-02	-5.50e-03	9.47e-03	9.95e-03	7.32e-03
x			1.60e-03	1.15e-02	-9.35e-03	9.39e-03	-4.99e-03	7.27e-03
x			-3.01e-03	1.23e-02	3.36e-03	1.02e-02	6.71e-04	7.84e-03
		x	2.46e-02	2.40e-02	-1.15e-02	1.99e-02	3.25e-03	1.54e-02
x			1.90e-02	1.53e-02	1.45e-02	1.26e-02	1.63e-02	9.73e-03
x			-2.82e-03	1.09e-02	7.77e-03	8.93e-03	3.42e-03	6.91e-03
x			4.05e-03	1.15e-02	-6.55e-04	9.47e-03	1.27e-03	7.33e-03
x			-1.69e-02	1.22e-02	1.01e-02	9.93e-03	-4.88e-04	7.70e-03
x			1.95e-02	1.09e-02	8.51e-03	8.96e-03	1.30e-02	6.93e-03
x			1.15e-02	1.31e-02	1.55e-02	1.07e-02	1.39e-02	8.30e-03
x			3.05e-02	1.16e-02	8.25e-03	9.45e-03	1.73e-02	7.32e-03
x			-3.07e-03	1.16e-02	2.09e-02	9.53e-03	1.13e-02	7.36e-03
x			2.09e-03	1.09e-02	2.11e-02	8.91e-03	1.35e-02	6.90e-03
x			-1.09e-02	1.12e-02	-1.86e-02	9.19e-03	-1.56e-02	7.11e-03
x			-1.05e-02	1.16e-02	2.95e-03	9.51e-03	-2.43e-03	7.35e-03
		x	7.88e-05	1.15e-02	-1.31e-02	9.49e-03	-7.81e-03	7.33e-03
x			1.86e-03	1.09e-02	-2.03e-02	8.97e-03	-1.14e-02	6.93e-03
		x	1.09e-02	1.30e-02	1.60e-02	1.07e-02	1.39e-02	8.28e-03
x			-9.92e-03	1.15e-02	-1.07e-02	9.48e-03	-1.04e-02	7.32e-03
x			-6.63e-03	1.25e-02	-1.80e-02	1.02e-02	-1.36e-02	7.92e-03
x			1.17e-02	1.12e-02	1.25e-02	9.22e-03	1.21e-02	7.13e-03
x			9.17e-03	1.26e-02	9.48e-03	1.04e-02	9.28e-03	8.01e-03
x			1.84e-02	1.09e-02	1.23e-02	8.95e-03	1.49e-02	6.92e-03
x			2.17e-02	1.24e-02	-8.77e-03	1.03e-02	3.45e-03	7.92e-03

x			4.50e-03	1.12e-02	-2.16e-02	9.16e-03	-1.11e-02	7.10e-03
x			2.35e-02	1.09e-02	1.29e-02	9.01e-03	1.71e-02	6.95e-03
x			2.90e-02	1.10e-02	1.46e-02	9.00e-03	2.06e-02	6.96e-03
x			1.84e-02	1.10e-02	1.19e-02	9.01e-03	1.45e-02	6.96e-03
x			2.04e-04	1.79e-02	-2.70e-03	1.48e-02	-1.53e-03	1.14e-02
		x	1.15e-02	1.10e-02	7.64e-03	8.97e-03	9.20e-03	6.95e-03
		x	2.14e-02	1.56e-02	1.84e-02	1.29e-02	2.00e-02	9.93e-03
		x	-1.09e-02	1.09e-02	-1.60e-02	8.94e-03	-1.40e-02	6.92e-03
x			4.61e-03	1.12e-02	8.92e-03	9.20e-03	7.21e-03	7.11e-03
x			1.13e-02	4.00e-02	-5.17e-02	3.32e-02	-2.65e-02	2.56e-02
x			-1.32e-03	1.15e-02	-4.15e-03	9.41e-03	-2.95e-03	7.28e-03
x			-4.46e-03	1.13e-02	8.17e-03	9.27e-03	3.21e-03	7.17e-03
x			-7.90e-03	1.54e-02	1.10e-02	1.26e-02	3.37e-03	9.77e-03
x			7.71e-03	1.09e-02	-1.02e-02	8.97e-03	-3.09e-03	6.94e-03
x			1.47e-02	1.36e-02	-2.28e-02	1.12e-02	-7.63e-03	8.67e-03
x			-8.64e-03	1.23e-02	-2.15e-02	1.01e-02	-1.60e-02	7.84e-03
		x	-1.34e-03	1.38e-02	-2.50e-03	1.13e-02	-1.94e-03	8.75e-03
x			6.53e-03	1.40e-02	2.43e-04	1.16e-02	2.82e-03	8.91e-03
x			-5.72e-02	2.04e-02	-2.02e-02	1.65e-02	-3.48e-02	1.28e-02
		x	-2.76e-03	1.98e-02	-8.51e-03	1.62e-02	-6.15e-03	1.26e-02
x			5.78e-03	1.10e-02	2.11e-02	9.03e-03	1.50e-02	6.98e-03
x			-3.27e-04	1.09e-02	-2.19e-03	8.94e-03	-1.45e-03	6.91e-03
		x	2.53e-02	1.12e-02	1.51e-02	9.22e-03	1.93e-02	7.12e-03
x			5.63e-03	1.29e-02	2.33e-02	1.05e-02	1.62e-02	8.15e-03
x			2.30e-02	1.10e-02	1.60e-02	9.02e-03	1.88e-02	6.98e-03
x			1.18e-02	1.30e-02	3.08e-04	1.07e-02	5.03e-03	8.27e-03
x			1.26e-02	1.11e-02	-4.29e-03	9.14e-03	2.56e-03	7.07e-03
x			-6.74e-04	1.10e-02	1.26e-02	9.01e-03	7.20e-03	6.97e-03
x			1.59e-02	1.11e-02	2.16e-02	9.02e-03	1.94e-02	6.99e-03
x			1.44e-02	1.10e-02	-2.78e-03	9.03e-03	4.11e-03	6.98e-03
x			-1.39e-02	1.50e-02	-1.61e-02	1.23e-02	-1.52e-02	9.50e-03
x			2.98e-03	1.09e-02	-3.30e-03	8.92e-03	-1.01e-03	6.90e-03
	x		-5.34e-04	1.09e-02	-1.84e-02	8.95e-03	-1.12e-02	6.93e-03
		x	-1.01e-02	1.11e-02	6.33e-03	9.12e-03	-2.06e-04	7.06e-03
x			-2.89e-02	1.16e-02	-1.03e-02	9.53e-03	-1.77e-02	7.37e-03
		x	2.97e-02	1.59e-02	-4.29e-03	1.32e-02	9.74e-03	1.02e-02
x			1.35e-02	1.18e-02	5.50e-03	9.74e-03	8.66e-03	7.53e-03
	x		-7.18e-03	1.22e-02	-1.56e-02	1.00e-02	-1.22e-02	7.73e-03
		x	-1.09e-03	1.11e-02	-3.22e-02	9.09e-03	-1.96e-02	7.04e-03
x			5.38e-02	1.78e-02	1.28e-02	1.48e-02	2.95e-02	1.14e-02
	x	x	-1.26e-02	1.14e-02	-3.45e-02	9.30e-03	-2.57e-02	7.19e-03
x			1.72e-02	1.40e-02	-8.74e-03	1.16e-02	1.85e-03	8.94e-03
x			1.55e-02	1.42e-02	-1.88e-02	1.17e-02	-5.05e-03	9.02e-03
x			1.49e-03	1.14e-02	-4.09e-03	9.39e-03	-1.92e-03	7.26e-03
		x	3.02e-02	1.54e-02	1.39e-02	1.27e-02	2.07e-02	9.78e-03
x			-4.45e-03	1.27e-02	-1.06e-02	1.04e-02	-8.03e-03	8.06e-03
x			1.12e-02	1.14e-02	-1.52e-02	9.35e-03	-4.76e-03	7.23e-03

x			3.71e-02	1.25e-02	1.38e-02	1.03e-02	2.34e-02	7.96e-03
x			-7.22e-04	1.12e-02	1.34e-03	9.18e-03	5.29e-04	7.09e-03
		x	2.75e-02	1.25e-02	4.29e-02	1.03e-02	3.67e-02	7.96e-03
x			5.98e-03	1.11e-02	-2.80e-03	9.09e-03	6.35e-04	7.03e-03
x			-1.22e-02	1.14e-02	-4.27e-03	9.38e-03	-7.55e-03	7.25e-03
x			-5.03e-03	1.44e-02	-1.11e-02	1.18e-02	-8.64e-03	9.12e-03
x			5.53e-03	1.09e-02	4.62e-03	8.93e-03	5.00e-03	6.91e-03
x			2.10e-02	1.11e-02	1.42e-02	9.11e-03	1.69e-02	7.05e-03
x			-3.62e-02	1.17e-02	-1.35e-02	9.51e-03	-2.25e-02	7.37e-03
x			9.84e-03	1.09e-02	4.91e-03	8.96e-03	6.90e-03	6.93e-03
		x	7.00e-03	1.41e-02	-1.73e-02	1.16e-02	-7.56e-03	8.97e-03
x			2.57e-02	1.25e-02	-5.23e-03	1.03e-02	7.26e-03	7.96e-03
x			2.66e-03	1.14e-02	6.21e-03	9.35e-03	4.84e-03	7.24e-03
x			-8.04e-03	1.09e-02	-1.29e-03	8.95e-03	-4.01e-03	6.93e-03
x			-4.08e-03	1.09e-02	-3.85e-03	8.98e-03	-3.94e-03	6.94e-03
x			1.14e-02	1.26e-02	1.61e-02	1.04e-02	1.42e-02	8.00e-03
		x	-2.08e-02	1.49e-02	-1.04e-02	1.21e-02	-1.45e-02	9.41e-03
		x	-1.49e-02	3.39e-02	-2.57e-02	2.78e-02	-2.16e-02	2.15e-02
x			2.73e-02	1.33e-02	-1.04e-02	1.10e-02	5.03e-03	8.46e-03
x			-2.90e-02	1.15e-02	-8.11e-03	9.39e-03	-1.66e-02	7.27e-03
	x		-4.56e-02	2.66e-02	-3.28e-03	2.15e-02	-2.03e-02	1.67e-02
x			-9.27e-03	2.10e-02	-1.84e-02	1.73e-02	-1.47e-02	1.34e-02
x			2.84e-03	1.13e-02	-2.47e-03	9.24e-03	-2.52e-04	7.15e-03
		x	-2.88e-02	1.17e-02	-4.19e-02	9.59e-03	-3.67e-02	7.41e-03
x			8.37e-03	1.15e-02	-8.25e-03	9.45e-03	-1.54e-03	7.31e-03
x			1.12e-02	1.15e-02	-2.27e-04	9.45e-03	4.14e-03	7.31e-03
x			-1.15e-02	1.10e-02	-1.09e-02	8.98e-03	-1.12e-02	6.95e-03
x			2.14e-02	1.10e-02	2.92e-02	9.02e-03	2.60e-02	6.98e-03
x			3.89e-03	1.38e-02	-8.85e-03	1.13e-02	-3.96e-03	8.76e-03
x			6.23e-03	1.23e-02	2.50e-02	1.01e-02	1.74e-02	7.79e-03
x			-6.73e-03	1.12e-02	-9.98e-03	9.17e-03	-8.52e-03	7.09e-03
x			-2.28e-02	1.13e-02	-2.91e-02	9.26e-03	-2.64e-02	7.17e-03
x			1.93e-02	1.25e-02	3.95e-03	1.03e-02	1.00e-02	7.96e-03
x			3.26e-03	1.09e-02	-2.55e-03	8.97e-03	-2.91e-04	6.93e-03
x			2.26e-02	1.10e-02	1.05e-02	8.95e-03	1.54e-02	6.93e-03
x			1.15e-02	1.09e-02	1.24e-02	8.93e-03	1.20e-02	6.91e-03
x			-8.41e-03	1.09e-02	2.22e-02	8.95e-03	9.77e-03	6.92e-03
x			6.16e-04	1.09e-02	5.00e-03	8.93e-03	3.31e-03	6.91e-03
x			-1.51e-02	1.48e-02	-3.55e-02	1.21e-02	-2.73e-02	9.38e-03
x			5.85e-03	1.12e-02	8.55e-03	9.18e-03	7.37e-03	7.11e-03
	x		-2.10e-02	1.13e-02	6.01e-03	9.27e-03	-4.95e-03	7.17e-03
x			7.94e-03	1.29e-02	-1.34e-02	1.06e-02	-4.69e-03	8.19e-03
x			8.85e-03	1.31e-02	1.19e-02	1.07e-02	1.09e-02	8.29e-03
x			-7.35e-03	1.20e-02	-1.58e-02	9.79e-03	-1.24e-02	7.58e-03
x			-5.09e-03	1.09e-02	1.24e-02	8.93e-03	5.44e-03	6.91e-03
x			7.46e-03	1.09e-02	-8.66e-03	8.99e-03	-2.28e-03	6.95e-03
x			-1.17e-02	2.59e-02	2.72e-02	2.11e-02	1.12e-02	1.63e-02

x			-5.06e-03	1.46e-02	-1.01e-02	1.19e-02	-7.92e-03	9.24e-03
x			9.82e-03	1.11e-02	1.38e-02	9.13e-03	1.22e-02	7.05e-03
x			5.41e-03	1.09e-02	5.35e-03	8.93e-03	5.52e-03	6.91e-03
x			-5.93e-03	1.19e-02	6.44e-04	9.80e-03	-1.99e-03	7.58e-03
x			7.51e-03	1.20e-02	-1.33e-02	9.83e-03	-4.77e-03	7.60e-03
x			-3.90e-02	1.36e-02	-1.50e-02	1.12e-02	-2.46e-02	8.64e-03
x			6.31e-03	1.90e-02	2.10e-02	1.56e-02	1.52e-02	1.21e-02
	x		2.93e-02	1.30e-02	1.86e-02	1.07e-02	2.30e-02	8.26e-03
	x	x	4.76e-03	1.10e-02	8.27e-03	9.01e-03	7.01e-03	6.97e-03
x			1.14e-02	1.10e-02	1.66e-02	9.01e-03	1.44e-02	6.96e-03
x			3.41e-03	1.60e-02	-1.58e-02	1.32e-02	-8.15e-03	1.02e-02
x			1.66e-02	1.54e-02	1.01e-02	1.26e-02	1.26e-02	9.78e-03
x			5.00e-03	1.13e-02	5.71e-03	9.24e-03	5.47e-03	7.15e-03
x			-8.60e-03	1.12e-02	5.57e-03	9.21e-03	-6.11e-05	7.11e-03
x			1.61e-02	1.09e-02	-1.57e-03	8.94e-03	5.43e-03	6.92e-03
x			2.18e-02	1.15e-02	5.86e-03	9.46e-03	1.23e-02	7.30e-03
x			-1.61e-02	1.24e-02	-4.51e-02	1.02e-02	-3.34e-02	7.88e-03
	x		-2.71e-02	1.11e-02	5.37e-03	9.08e-03	-7.80e-03	7.02e-03
x			-9.63e-03	1.48e-02	-1.93e-03	1.21e-02	-4.88e-03	9.40e-03
	x		1.54e-02	1.92e-02	1.71e-02	1.57e-02	1.64e-02	1.22e-02
		x	-2.61e-03	1.10e-02	-4.25e-03	9.02e-03	-3.56e-03	6.98e-03
x			2.37e-02	1.09e-02	1.17e-02	8.99e-03	1.65e-02	6.94e-03
x			9.31e-03	1.27e-02	8.97e-03	1.05e-02	8.98e-03	8.10e-03
x			5.36e-03	1.12e-02	2.47e-02	9.14e-03	1.69e-02	7.08e-03
x			-3.82e-02	2.58e-02	-4.01e-02	2.11e-02	-3.98e-02	1.63e-02
x			-1.52e-02	2.51e-02	1.81e-02	2.04e-02	4.79e-03	1.58e-02
x			-1.95e-03	1.10e-02	1.06e-02	9.02e-03	5.52e-03	6.97e-03
x			-8.71e-03	1.13e-02	-1.38e-02	9.26e-03	-1.17e-02	7.17e-03
x			5.15e-02	3.56e-02	-2.57e-02	3.01e-02	6.26e-03	2.30e-02
x			-2.70e-02	1.59e-02	-1.66e-03	1.30e-02	-1.17e-02	1.01e-02
x			9.84e-03	1.90e-02	4.03e-03	1.56e-02	6.45e-03	1.20e-02
x			3.50e-03	1.56e-02	3.00e-02	1.28e-02	1.95e-02	9.91e-03
x			-1.98e-02	1.39e-02	-1.74e-02	1.14e-02	-1.86e-02	8.82e-03
x			-1.50e-02	1.31e-02	-2.57e-03	1.07e-02	-7.54e-03	8.27e-03
x			-1.41e-02	1.10e-02	9.41e-03	8.99e-03	3.79e-06	6.96e-03
x			-1.02e-02	1.14e-02	1.90e-03	9.36e-03	-2.87e-03	7.24e-03
x			-4.13e-03	1.20e-02	-4.96e-03	9.88e-03	-4.55e-03	7.64e-03
x			1.24e-02	1.09e-02	2.74e-02	8.93e-03	2.13e-02	6.90e-03
		x	-1.70e-02	1.09e-02	-1.00e-02	8.92e-03	-1.29e-02	6.90e-03
x			3.82e-03	1.09e-02	9.03e-04	8.97e-03	2.30e-03	6.94e-03
x			-9.24e-03	1.11e-02	-9.97e-03	9.10e-03	-9.85e-03	7.04e-03
		x	-3.66e-02	1.11e-02	-2.98e-02	9.06e-03	-3.24e-02	7.02e-03
		x	-2.23e-02	1.74e-02	-8.70e-03	1.45e-02	-1.45e-02	1.11e-02
x			9.89e-03	1.16e-02	-6.83e-03	9.49e-03	-1.23e-04	7.34e-03
x			7.13e-03	1.44e-02	1.96e-02	1.18e-02	1.50e-02	9.13e-03
	x		-2.14e-02	1.44e-02	-4.24e-03	1.17e-02	-1.11e-02	9.07e-03
		x	-3.32e-02	1.10e-02	-5.33e-03	9.05e-03	-1.66e-02	6.99e-03

x			-2.63e-02	1.11e-02	-1.84e-02	9.03e-03	-2.16e-02	7.00e-03
x			-6.96e-03	1.12e-02	-1.43e-03	9.15e-03	-3.48e-03	7.08e-03
x			1.34e-02	1.08e-02	-4.17e-04	8.90e-03	5.19e-03	6.89e-03
x			-3.98e-03	1.18e-02	1.30e-02	9.64e-03	6.02e-03	7.47e-03
x			-1.33e-02	1.10e-02	-1.90e-02	9.02e-03	-1.67e-02	6.99e-03
x			-7.51e-03	1.10e-02	-8.00e-03	9.03e-03	-7.76e-03	6.98e-03
x			8.66e-03	1.11e-02	1.48e-02	9.15e-03	1.24e-02	7.07e-03
x			3.80e-02	1.33e-02	1.62e-02	1.09e-02	2.50e-02	8.42e-03
x			1.11e-02	2.05e-02	-1.18e-02	1.69e-02	-2.85e-03	1.30e-02
	x		3.22e-02	1.70e-02	2.97e-02	1.41e-02	3.07e-02	1.09e-02
x			3.06e-03	1.75e-02	-3.19e-02	1.44e-02	-1.77e-02	1.12e-02
x			-1.93e-02	1.10e-02	-1.17e-03	9.07e-03	-8.51e-03	7.01e-03
x			3.03e-02	1.10e-02	3.87e-02	8.99e-03	3.53e-02	6.95e-03
x			-2.97e-02	1.12e-02	-2.56e-02	9.17e-03	-2.72e-02	7.10e-03
x			2.21e-02	1.61e-02	1.68e-02	1.32e-02	1.89e-02	1.02e-02
x			-1.39e-02	1.47e-02	-1.67e-02	1.21e-02	-1.54e-02	9.36e-03
	x		1.68e-03	1.15e-02	-8.22e-03	9.49e-03	-4.25e-03	7.33e-03
	x		-3.10e-02	1.53e-02	1.04e-02	1.24e-02	-6.15e-03	9.66e-03
x			-1.27e-02	1.58e-02	-3.23e-03	1.30e-02	-7.12e-03	1.00e-02
x			-2.14e-02	1.32e-02	-1.10e-02	1.08e-02	-1.53e-02	8.37e-03
		x	-4.16e-02	1.50e-02	-1.11e-02	1.22e-02	-2.32e-02	9.44e-03
		x	1.32e-02	1.70e-02	-3.03e-02	1.41e-02	-1.26e-02	1.08e-02
x			-2.28e-02	1.34e-02	1.26e-02	1.10e-02	-1.57e-03	8.51e-03
x			-1.52e-02	1.09e-02	5.92e-03	8.91e-03	-2.65e-03	6.89e-03
x			1.79e-02	1.10e-02	1.99e-02	9.01e-03	1.90e-02	6.97e-03
		x	1.70e-02	1.10e-02	2.71e-02	8.98e-03	2.31e-02	6.95e-03
x			-4.25e-03	2.09e-02	2.11e-02	1.70e-02	1.13e-02	1.32e-02
x			6.81e-03	1.18e-02	4.90e-03	9.72e-03	5.63e-03	7.51e-03
x			-1.43e-02	1.31e-02	-3.81e-03	1.07e-02	-8.14e-03	8.29e-03
x			-8.03e-02	2.75e-02	-1.15e-02	2.18e-02	-3.86e-02	1.71e-02
x			-1.43e-02	1.12e-02	-2.95e-02	9.20e-03	-2.34e-02	7.12e-03
		x	-2.83e-02	1.14e-02	-1.71e-02	9.34e-03	-2.17e-02	7.23e-03
x			-2.26e-02	1.39e-02	-2.55e-02	1.14e-02	-2.44e-02	8.82e-03
x			-5.32e-03	1.16e-02	3.58e-03	9.53e-03	-8.83e-07	7.37e-03
		x	6.47e-03	1.32e-02	8.05e-03	1.08e-02	7.24e-03	8.35e-03
x			-3.09e-02	1.52e-02	-2.86e-02	1.24e-02	-2.96e-02	9.59e-03
	x		4.92e-03	1.09e-02	8.72e-03	8.96e-03	7.16e-03	6.92e-03
x			1.31e-02	1.62e-02	2.44e-02	1.33e-02	2.01e-02	1.03e-02
x			-1.24e-02	1.09e-02	4.77e-03	8.93e-03	-2.00e-03	6.90e-03
x			-2.09e-02	1.14e-02	-3.17e-02	9.36e-03	-2.74e-02	7.24e-03
x			-3.35e-03	1.25e-02	1.88e-02	1.03e-02	9.78e-03	7.97e-03
		x	-3.65e-02	1.44e-02	-2.91e-02	1.18e-02	-3.20e-02	9.15e-03
		x	-2.65e-02	1.10e-02	-2.09e-02	8.98e-03	-2.31e-02	6.95e-03
x			5.03e-03	1.32e-02	1.70e-02	1.08e-02	1.22e-02	8.36e-03
		x	-3.73e-03	1.18e-02	-8.55e-03	9.69e-03	-6.60e-03	7.50e-03
		x	-1.24e-02	1.10e-02	-1.79e-02	9.02e-03	-1.59e-02	6.98e-03
x			1.64e-02	1.11e-02	-1.39e-02	9.08e-03	-1.84e-03	7.03e-03

x			-1.19e-02	1.21e-02	-4.07e-03	9.93e-03	-7.32e-03	7.69e-03
x			2.45e-02	1.13e-02	1.46e-02	9.27e-03	1.86e-02	7.18e-03
x			-3.35e-03	1.18e-02	1.79e-02	9.79e-03	9.23e-03	7.55e-03
x			-6.58e-04	1.16e-02	-1.65e-02	9.55e-03	-9.92e-03	7.38e-03
		x	1.28e-02	1.16e-02	2.78e-02	9.49e-03	2.19e-02	7.34e-03
		x	-5.10e-03	1.23e-02	-8.11e-03	1.01e-02	-6.79e-03	7.79e-03
		x	-1.60e-03	1.25e-02	-1.14e-02	1.02e-02	-7.43e-03	7.92e-03
x			-9.48e-03	1.10e-02	2.06e-02	9.05e-03	8.46e-03	7.00e-03
x			1.53e-02	1.26e-02	2.20e-02	1.03e-02	1.92e-02	7.97e-03
x			-4.09e-04	1.21e-02	-9.61e-03	9.94e-03	-5.86e-03	7.69e-03
x			4.38e-03	1.11e-02	5.23e-04	9.11e-03	1.97e-03	7.05e-03
x			-2.19e-02	1.20e-02	-5.59e-03	9.84e-03	-1.21e-02	7.62e-03
x			-7.68e-03	1.09e-02	-5.37e-03	9.00e-03	-6.40e-03	6.96e-03
x			-9.16e-03	1.09e-02	-1.55e-02	8.94e-03	-1.30e-02	6.92e-03
x			5.15e-03	1.22e-02	-5.19e-03	1.01e-02	-9.63e-04	7.79e-03
x			2.71e-02	1.18e-02	6.68e-03	9.63e-03	1.48e-02	7.46e-03
x			2.85e-03	1.17e-02	1.99e-03	9.56e-03	2.34e-03	7.40e-03
x			-1.90e-03	1.12e-02	6.95e-03	9.22e-03	3.28e-03	7.12e-03
		x	1.66e-02	1.09e-02	2.23e-02	8.97e-03	1.99e-02	6.93e-03
x			-1.35e-02	1.09e-02	-3.96e-03	8.91e-03	-7.60e-03	6.89e-03
x			-2.71e-02	1.18e-02	-8.56e-03	9.72e-03	-1.61e-02	7.51e-03
x			-1.58e-02	2.04e-02	-8.63e-03	1.67e-02	-1.16e-02	1.29e-02
	x		-1.11e-02	1.22e-02	9.25e-03	1.00e-02	1.09e-03	7.77e-03
x			-1.32e-02	1.10e-02	-9.09e-03	8.97e-03	-1.08e-02	6.95e-03
x			-3.50e-03	1.12e-02	3.97e-03	9.14e-03	1.00e-03	7.08e-03
x			1.90e-03	1.16e-02	2.34e-03	9.53e-03	2.11e-03	7.36e-03
		x	-5.83e-02	5.73e-02	3.04e-02	4.44e-02	-3.50e-03	3.50e-02
x			6.33e-03	1.09e-02	6.57e-03	8.91e-03	6.60e-03	6.89e-03
x			3.36e-02	1.21e-02	1.97e-03	9.95e-03	1.46e-02	7.69e-03
		x	1.45e-03	1.11e-02	5.15e-03	9.08e-03	3.64e-03	7.02e-03
x			-3.32e-02	1.30e-02	2.50e-03	1.07e-02	-1.20e-02	8.28e-03
x			2.01e-03	1.10e-02	-1.04e-02	9.02e-03	-5.49e-03	6.98e-03

D female B	HD female S	HD male Be	CHD male S	D overall B	HD overall S	I female Be	MI female S	MI male Bet
-2.57e-03	1.25e-02	1.15e-03	9.69e-03	-8.53e-05	7.65e-03	-3.79e-03	2.26e-02	-7.99e-03
6.62e-03	1.22e-02	9.17e-03	9.48e-03	8.43e-03	7.49e-03	-6.97e-03	2.21e-02	6.73e-03
8.13e-03	1.25e-02	1.20e-02	9.71e-03	1.05e-02	7.67e-03	1.07e-02	2.26e-02	-2.47e-03
-3.76e-03	1.25e-02	-1.50e-02	9.71e-03	-1.09e-02	7.67e-03	-4.44e-02	2.27e-02	-1.43e-02
-3.57e-03	1.21e-02	1.44e-02	9.44e-03	8.07e-03	7.46e-03	1.94e-02	2.20e-02	2.86e-02
2.23e-02	1.22e-02	1.64e-02	9.47e-03	1.85e-02	7.47e-03	3.46e-02	2.20e-02	1.94e-02
6.98e-04	1.29e-02	7.26e-03	1.01e-02	4.95e-03	7.96e-03	-1.44e-02	2.34e-02	2.03e-02
1.09e-03	1.21e-02	1.00e-02	9.44e-03	6.63e-03	7.46e-03	1.99e-03	2.20e-02	-3.12e-03
-5.79e-03	1.30e-02	-2.68e-02	1.02e-02	-1.87e-02	8.03e-03	-7.66e-03	2.36e-02	-1.13e-02
1.07e-02	1.25e-02	1.63e-02	9.75e-03	1.40e-02	7.70e-03	2.71e-02	2.27e-02	-5.60e-03
1.38e-02	1.30e-02	1.72e-02	1.01e-02	1.59e-02	7.95e-03	1.91e-02	2.35e-02	3.15e-02
-1.80e-03	1.24e-02	-2.55e-03	9.64e-03	-2.28e-03	7.62e-03	-2.30e-03	2.25e-02	-1.85e-02
1.27e-03	1.49e-02	8.81e-03	1.16e-02	6.02e-03	9.15e-03	1.25e-02	2.69e-02	1.93e-02
5.74e-03	1.23e-02	6.40e-03	9.57e-03	6.29e-03	7.56e-03	-1.03e-02	2.23e-02	9.22e-03
3.32e-02	2.56e-02	7.59e-03	2.02e-02	1.75e-02	1.59e-02	5.75e-02	4.59e-02	2.22e-03
4.50e-03	1.27e-02	-3.31e-03	9.85e-03	-2.50e-04	7.78e-03	-2.00e-02	2.29e-02	-1.76e-02
5.69e-03	1.28e-02	-1.15e-02	9.98e-03	-4.96e-03	7.89e-03	3.31e-02	2.32e-02	6.16e-03
3.14e-02	1.56e-02	6.50e-02	1.21e-02	5.23e-02	9.56e-03	3.79e-02	2.82e-02	5.71e-02
-1.13e-02	1.24e-02	-2.10e-02	9.65e-03	-1.75e-02	7.63e-03	1.83e-02	2.25e-02	-3.53e-02
-1.47e-02	1.43e-02	-1.48e-02	1.12e-02	-1.48e-02	8.81e-03	-8.72e-03	2.59e-02	-1.39e-02
-1.12e-02	1.57e-02	1.51e-02	1.21e-02	5.24e-03	9.56e-03	-2.54e-02	2.85e-02	3.15e-02
-2.71e-02	1.24e-02	-5.48e-04	9.63e-03	-1.04e-02	7.60e-03	-1.24e-02	2.24e-02	3.85e-03
1.91e-02	1.32e-02	2.35e-02	1.03e-02	2.19e-02	8.12e-03	-2.59e-02	2.41e-02	3.10e-02
-1.80e-02	1.24e-02	-9.04e-03	9.68e-03	-1.25e-02	7.64e-03	-1.95e-02	2.25e-02	-1.08e-02
8.86e-03	1.23e-02	1.07e-02	9.57e-03	1.01e-02	7.56e-03	-2.97e-02	2.23e-02	-2.45e-03
2.79e-02	1.54e-02	-7.16e-03	1.20e-02	6.06e-03	9.50e-03	3.20e-02	2.79e-02	-1.37e-03
3.27e-03	1.64e-02	-1.65e-02	1.28e-02	-8.80e-03	1.01e-02	-2.83e-02	3.01e-02	-5.20e-03
-1.33e-02	1.22e-02	-2.93e-03	9.48e-03	-6.69e-03	7.49e-03	-1.77e-02	2.21e-02	1.49e-02
-6.79e-03	1.27e-02	1.12e-03	9.80e-03	-1.86e-03	7.76e-03	-9.86e-03	2.30e-02	2.30e-02
7.79e-03	1.32e-02	4.67e-03	1.02e-02	5.93e-03	8.09e-03	1.81e-02	2.38e-02	1.43e-02
-3.11e-02	2.02e-02	-3.86e-02	1.56e-02	-3.58e-02	1.24e-02	-3.19e-02	3.67e-02	-6.55e-02
1.43e-02	1.56e-02	2.01e-02	1.22e-02	1.80e-02	9.61e-03	4.45e-02	2.81e-02	1.58e-02
-2.04e-02	1.25e-02	-1.72e-02	9.69e-03	-1.87e-02	7.65e-03	2.08e-03	2.26e-02	-1.99e-02
1.05e-02	1.64e-02	3.44e-02	1.27e-02	2.54e-02	1.00e-02	4.63e-02	2.93e-02	4.67e-02
-1.76e-02	1.24e-02	-2.47e-02	9.59e-03	-2.20e-02	7.59e-03	-3.37e-02	2.25e-02	-1.79e-02
1.25e-02	1.22e-02	4.98e-03	9.46e-03	7.74e-03	7.47e-03	1.81e-02	2.20e-02	1.18e-02
4.12e-02	1.90e-02	1.55e-02	1.49e-02	2.55e-02	1.18e-02	3.59e-02	3.44e-02	-2.04e-02
-8.11e-03	2.55e-02	-2.06e-03	1.99e-02	-3.99e-03	1.57e-02	9.66e-03	4.58e-02	-6.22e-03
1.61e-03	2.31e-02	-3.21e-02	1.79e-02	-1.97e-02	1.42e-02	-5.05e-03	4.18e-02	-7.70e-02
5.08e-03	1.27e-02	1.74e-02	9.89e-03	1.28e-02	7.81e-03	1.77e-02	2.30e-02	1.39e-02
-1.06e-02	1.62e-02	4.26e-03	1.25e-02	-1.40e-03	9.92e-03	1.02e-04	2.93e-02	-1.31e-04
2.53e-02	1.22e-02	2.29e-02	9.43e-03	2.36e-02	7.46e-03	-8.67e-03	2.21e-02	1.01e-02
1.95e-03	1.43e-02	-2.38e-02	1.12e-02	-1.39e-02	8.83e-03	-1.39e-02	2.60e-02	-3.08e-02
-1.58e-02	1.23e-02	1.37e-02	9.56e-03	2.62e-03	7.55e-03	-3.39e-02	2.22e-02	1.63e-02
-1.61e-02	1.91e-02	6.86e-03	1.48e-02	-1.47e-03	1.17e-02	-1.77e-02	3.48e-02	1.47e-02
-2.80e-02	1.30e-02	-8.53e-03	1.01e-02	-1.58e-02	7.97e-03	-1.98e-02	2.35e-02	-1.40e-02

9.90e-03	1.22e-02	6.33e-04	9.46e-03	4.07e-03	7.47e-03	8.36e-03	2.21e-02	1.21e-02
5.80e-04	1.98e-02	3.34e-02	1.53e-02	2.08e-02	1.21e-02	9.14e-03	3.58e-02	4.97e-02
-4.29e-02	1.57e-02	-2.35e-02	1.22e-02	-3.10e-02	9.66e-03	-8.40e-02	2.81e-02	-4.13e-02
1.59e-02	1.40e-02	5.33e-04	1.09e-02	6.15e-03	8.59e-03	2.91e-02	2.52e-02	-7.58e-05
4.36e-03	1.21e-02	-1.33e-02	9.42e-03	-6.74e-03	7.43e-03	-1.61e-02	2.19e-02	-2.87e-02
3.53e-02	1.40e-02	1.59e-02	1.09e-02	2.34e-02	8.60e-03	2.49e-02	2.53e-02	1.22e-02
-1.80e-02	1.92e-02	-5.35e-03	1.49e-02	-9.72e-03	1.18e-02	2.84e-02	3.44e-02	1.40e-02
-5.19e-02	1.83e-02	-3.06e-02	1.41e-02	-3.85e-02	1.12e-02	-5.71e-02	3.33e-02	-9.18e-03
1.12e-02	1.39e-02	2.38e-02	1.07e-02	1.90e-02	8.50e-03	2.60e-02	2.50e-02	1.94e-02
3.17e-03	1.65e-02	-9.02e-03	1.28e-02	-4.74e-03	1.01e-02	3.65e-02	2.96e-02	-1.91e-02
-2.95e-02	2.72e-02	5.28e-02	2.08e-02	2.15e-02	1.65e-02	-1.03e-01	5.09e-02	4.29e-03
-2.35e-02	1.27e-02	-2.48e-03	9.92e-03	-1.05e-02	7.82e-03	-3.13e-02	2.30e-02	-1.53e-03
-1.19e-02	1.30e-02	4.11e-04	1.01e-02	-4.05e-03	7.99e-03	-1.20e-04	2.36e-02	-1.55e-02
6.62e-03	1.60e-02	3.07e-02	1.23e-02	2.19e-02	9.76e-03	2.28e-02	2.88e-02	2.98e-02
-1.43e-02	1.83e-02	2.42e-02	1.41e-02	9.69e-03	1.12e-02	2.47e-02	3.27e-02	3.06e-03
-7.31e-03	1.34e-02	3.19e-03	1.04e-02	-6.94e-04	8.22e-03	1.59e-03	2.43e-02	1.83e-02
6.72e-04	1.22e-02	7.81e-03	9.48e-03	5.16e-03	7.49e-03	3.27e-02	2.21e-02	-2.70e-03
2.11e-02	1.38e-02	4.98e-03	1.07e-02	1.12e-02	8.45e-03	3.32e-02	2.48e-02	7.41e-03
2.14e-02	1.27e-02	1.43e-02	9.91e-03	1.69e-02	7.83e-03	1.98e-02	2.31e-02	1.23e-02
-1.18e-02	1.22e-02	-7.67e-04	9.47e-03	-4.64e-03	7.48e-03	-2.74e-02	2.20e-02	3.73e-03
2.15e-02	1.53e-02	1.29e-02	1.20e-02	1.62e-02	9.46e-03	-2.98e-03	2.80e-02	1.95e-02
2.23e-02	1.32e-02	-1.02e-02	1.02e-02	2.12e-03	8.10e-03	-2.47e-02	2.38e-02	1.47e-02
-2.49e-02	1.89e-02	1.28e-02	1.45e-02	-1.23e-03	1.15e-02	-3.28e-02	3.44e-02	7.23e-03
2.98e-02	1.28e-02	-5.55e-03	1.00e-02	7.91e-03	7.91e-03	1.05e-02	2.33e-02	1.26e-02
4.30e-03	1.28e-02	-1.09e-02	9.93e-03	-5.25e-03	7.86e-03	3.42e-03	2.33e-02	-3.06e-02
-9.84e-03	1.38e-02	1.32e-03	1.07e-02	-3.03e-03	8.47e-03	-3.84e-02	2.48e-02	7.78e-03
3.60e-02	2.66e-02	-1.99e-02	2.11e-02	1.68e-03	1.66e-02	2.28e-02	4.83e-02	1.22e-02
2.57e-02	1.70e-02	1.84e-02	1.33e-02	2.10e-02	1.05e-02	5.16e-02	3.05e-02	8.50e-03
-7.20e-03	1.22e-02	1.24e-02	9.44e-03	4.93e-03	7.46e-03	-1.82e-02	2.20e-02	8.88e-03
-3.88e-03	1.29e-02	-4.65e-03	1.00e-02	-4.36e-03	7.91e-03	-1.37e-02	2.33e-02	-1.34e-02
-1.73e-02	1.36e-02	2.21e-02	1.05e-02	7.65e-03	8.31e-03	-2.38e-02	2.47e-02	1.55e-02
2.03e-02	1.22e-02	7.27e-03	9.48e-03	1.23e-02	7.48e-03	2.63e-02	2.21e-02	-1.00e-03
-2.69e-03	1.47e-02	1.50e-02	1.13e-02	8.27e-03	8.97e-03	2.83e-02	2.63e-02	1.70e-02
3.24e-02	1.29e-02	5.13e-04	9.98e-03	1.26e-02	7.91e-03	4.19e-02	2.35e-02	1.74e-02
-6.15e-03	1.29e-02	2.53e-02	1.01e-02	1.34e-02	7.95e-03	-7.76e-03	2.34e-02	1.62e-02
7.96e-03	1.21e-02	2.03e-02	9.42e-03	1.57e-02	7.45e-03	4.01e-02	2.20e-02	2.25e-02
-1.80e-02	1.25e-02	-2.07e-02	9.72e-03	-1.97e-02	7.68e-03	-3.86e-02	2.28e-02	-1.59e-02
-1.47e-02	1.29e-02	2.17e-03	1.00e-02	-4.17e-03	7.94e-03	3.27e-02	2.33e-02	-3.93e-03
1.50e-02	1.29e-02	-1.36e-02	1.00e-02	-2.71e-03	7.91e-03	3.33e-02	2.32e-02	-1.93e-02
-2.89e-03	1.22e-02	-2.71e-02	9.48e-03	-1.80e-02	7.49e-03	7.97e-03	2.21e-02	-4.81e-02
2.26e-02	1.46e-02	1.79e-02	1.13e-02	1.96e-02	8.95e-03	1.70e-02	2.64e-02	2.38e-02
-1.41e-02	1.28e-02	-6.79e-03	1.00e-02	-9.57e-03	7.91e-03	-5.20e-03	2.33e-02	1.09e-02
-1.50e-02	1.39e-02	-1.75e-02	1.08e-02	-1.68e-02	8.56e-03	6.45e-03	2.52e-02	-2.16e-02
1.40e-02	1.25e-02	1.71e-02	9.74e-03	1.58e-02	7.70e-03	9.93e-03	2.27e-02	2.49e-02
7.31e-03	1.41e-02	4.58e-03	1.10e-02	5.53e-03	8.66e-03	1.97e-02	2.54e-02	-1.98e-02
2.66e-02	1.22e-02	1.41e-02	9.46e-03	1.90e-02	7.47e-03	5.21e-02	2.20e-02	1.57e-02
9.45e-03	1.39e-02	-1.28e-02	1.08e-02	-4.57e-03	8.56e-03	1.36e-02	2.52e-02	-2.46e-02

-6.54e-03	1.25e-02	-1.52e-02	9.69e-03	-1.20e-02	7.67e-03	-4.00e-02	2.28e-02	-1.21e-02
2.03e-02	1.22e-02	8.21e-03	9.52e-03	1.27e-02	7.51e-03	7.47e-04	2.21e-02	2.38e-02
2.52e-02	1.22e-02	1.80e-02	9.51e-03	2.09e-02	7.52e-03	6.56e-03	2.22e-02	2.60e-02
1.65e-02	1.22e-02	1.18e-02	9.53e-03	1.36e-02	7.52e-03	1.19e-02	2.22e-02	-1.39e-02
-5.18e-03	2.01e-02	-1.32e-04	1.56e-02	-2.10e-03	1.23e-02	-3.54e-02	3.68e-02	-2.16e-02
1.44e-02	1.22e-02	1.44e-02	9.48e-03	1.45e-02	7.50e-03	2.49e-02	2.22e-02	8.92e-03
9.07e-03	1.74e-02	1.92e-02	1.36e-02	1.58e-02	1.07e-02	-2.82e-02	3.20e-02	1.20e-02
-1.14e-02	1.22e-02	-1.59e-02	9.45e-03	-1.43e-02	7.47e-03	-1.86e-02	2.21e-02	3.51e-03
1.06e-02	1.25e-02	5.66e-03	9.73e-03	7.57e-03	7.68e-03	2.90e-02	2.26e-02	2.36e-03
-2.65e-03	4.49e-02	-5.63e-02	3.52e-02	-3.65e-02	2.77e-02	4.67e-02	7.98e-02	-8.12e-02
8.20e-03	1.28e-02	-6.30e-03	9.95e-03	-6.28e-04	7.86e-03	-1.26e-02	2.32e-02	-1.10e-02
8.32e-04	1.26e-02	5.21e-03	9.80e-03	3.65e-03	7.74e-03	-1.92e-02	2.29e-02	7.45e-03
-1.13e-02	1.71e-02	9.92e-03	1.34e-02	1.87e-03	1.05e-02	1.45e-02	3.13e-02	-8.75e-03
1.86e-02	1.22e-02	-1.11e-02	9.48e-03	-3.38e-05	7.49e-03	5.20e-02	2.21e-02	-2.04e-02
1.15e-02	1.52e-02	-2.37e-02	1.19e-02	-1.04e-02	9.37e-03	-3.64e-02	2.79e-02	-2.07e-02
-2.03e-02	1.37e-02	-1.73e-02	1.07e-02	-1.81e-02	8.46e-03	-1.14e-02	2.50e-02	-7.84e-04
-1.95e-02	1.53e-02	-8.26e-03	1.19e-02	-1.25e-02	9.43e-03	-7.03e-02	2.74e-02	-2.17e-02
1.55e-02	1.56e-02	4.55e-03	1.22e-02	8.80e-03	9.61e-03	-8.94e-03	2.84e-02	-2.48e-02
-5.29e-02	2.28e-02	-2.38e-02	1.75e-02	-3.44e-02	1.39e-02	-7.28e-02	4.18e-02	-1.64e-02
1.56e-03	2.21e-02	-7.95e-03	1.71e-02	-4.43e-03	1.36e-02	4.43e-02	3.95e-02	3.59e-02
6.26e-03	1.23e-02	2.38e-02	9.54e-03	1.72e-02	7.53e-03	4.99e-02	2.22e-02	4.24e-02
2.23e-03	1.21e-02	-2.15e-03	9.46e-03	-4.12e-04	7.46e-03	8.29e-03	2.20e-02	-1.05e-03
2.42e-02	1.25e-02	2.08e-02	9.74e-03	2.20e-02	7.69e-03	-2.61e-02	2.28e-02	2.76e-02
-2.80e-04	1.44e-02	3.22e-02	1.11e-02	2.00e-02	8.79e-03	-2.33e-02	2.62e-02	3.62e-02
2.52e-02	1.23e-02	1.40e-02	9.53e-03	1.82e-02	7.53e-03	-6.82e-05	2.23e-02	7.95e-03
2.35e-02	1.46e-02	-7.84e-04	1.13e-02	8.45e-03	8.93e-03	-1.14e-02	2.62e-02	-3.52e-03
1.79e-02	1.24e-02	2.70e-03	9.66e-03	8.50e-03	7.64e-03	4.13e-02	2.25e-02	5.84e-03
-1.36e-02	1.23e-02	1.43e-02	9.53e-03	3.76e-03	7.53e-03	2.03e-02	2.22e-02	-6.41e-05
1.35e-02	1.23e-02	1.68e-02	9.54e-03	1.58e-02	7.55e-03	7.75e-03	2.24e-02	1.30e-02
1.04e-02	1.23e-02	-4.44e-03	9.55e-03	1.16e-03	7.54e-03	6.01e-02	2.23e-02	1.68e-03
-2.08e-02	1.68e-02	-1.38e-02	1.30e-02	-1.64e-02	1.03e-02	-2.69e-03	3.02e-02	-9.87e-03
1.02e-02	1.21e-02	-8.21e-03	9.43e-03	-1.53e-03	7.45e-03	4.09e-02	2.20e-02	9.05e-03
-4.40e-03	1.22e-02	-1.74e-02	9.46e-03	-1.25e-02	7.48e-03	1.21e-02	2.21e-02	-3.95e-02
-7.23e-03	1.24e-02	1.40e-02	9.65e-03	6.08e-03	7.62e-03	-9.55e-03	2.25e-02	-1.38e-02
-2.50e-02	1.30e-02	-6.72e-03	1.01e-02	-1.35e-02	7.96e-03	-2.88e-02	2.35e-02	-1.46e-02
1.37e-02	1.78e-02	-7.29e-03	1.40e-02	9.38e-04	1.10e-02	7.40e-02	3.16e-02	-8.26e-03
8.52e-04	1.32e-02	5.48e-03	1.03e-02	3.61e-03	8.13e-03	-1.95e-02	2.38e-02	-9.49e-04
-2.91e-03	1.36e-02	-6.38e-03	1.06e-02	-5.07e-03	8.34e-03	-1.38e-02	2.47e-02	4.65e-03
3.53e-03	1.24e-02	-3.28e-02	9.61e-03	-1.90e-02	7.60e-03	1.62e-02	2.24e-02	-4.90e-02
5.71e-02	1.98e-02	1.48e-02	1.56e-02	3.10e-02	1.23e-02	1.04e-01	3.52e-02	1.89e-02
-2.01e-02	1.27e-02	-3.63e-02	9.82e-03	-3.02e-02	7.76e-03	-2.08e-02	2.29e-02	-4.42e-02
1.85e-02	1.56e-02	-1.01e-02	1.23e-02	7.90e-04	9.65e-03	-8.72e-03	2.85e-02	-9.63e-03
8.89e-03	1.58e-02	-1.39e-02	1.23e-02	-5.38e-03	9.74e-03	3.97e-02	2.84e-02	8.23e-03
-1.76e-03	1.28e-02	1.71e-04	9.93e-03	-6.38e-04	7.84e-03	-1.10e-02	2.31e-02	-8.73e-03
4.37e-02	1.71e-02	2.40e-02	1.34e-02	3.19e-02	1.05e-02	9.06e-02	3.05e-02	3.09e-02
-1.51e-03	1.41e-02	-1.06e-02	1.10e-02	-7.03e-03	8.71e-03	-2.43e-02	2.58e-02	-2.08e-02
7.32e-03	1.27e-02	-2.10e-02	9.88e-03	-1.04e-02	7.80e-03	-2.28e-02	2.30e-02	-2.54e-02

5.13e-02	1.39e-02	1.48e-02	1.09e-02	2.88e-02	8.59e-03	7.52e-02	2.50e-02	6.10e-03
1.09e-03	1.25e-02	8.83e-03	9.71e-03	5.96e-03	7.66e-03	3.91e-02	2.27e-02	8.32e-03
3.76e-02	1.40e-02	4.69e-02	1.09e-02	4.34e-02	8.61e-03	1.28e-01	2.60e-02	4.66e-02
4.65e-03	1.24e-02	-2.04e-03	9.61e-03	3.53e-04	7.59e-03	-2.87e-02	2.23e-02	-1.01e-02
-1.08e-02	1.28e-02	-4.86e-03	9.92e-03	-7.14e-03	7.83e-03	-3.49e-03	2.31e-02	-2.95e-03
1.10e-02	1.60e-02	-7.07e-03	1.25e-02	-3.29e-04	9.83e-03	-1.44e-02	2.91e-02	-1.32e-02
1.13e-02	1.21e-02	-7.13e-04	9.44e-03	3.86e-03	7.46e-03	2.17e-03	2.20e-02	9.81e-03
2.37e-02	1.24e-02	1.40e-02	9.63e-03	1.77e-02	7.61e-03	3.19e-02	2.25e-02	3.31e-02
-2.97e-02	1.30e-02	-7.96e-03	1.00e-02	-1.61e-02	7.95e-03	-1.86e-02	2.35e-02	-4.18e-03
2.91e-03	1.22e-02	5.80e-03	9.47e-03	4.75e-03	7.48e-03	-2.94e-02	2.21e-02	-2.31e-03
2.04e-03	1.57e-02	-1.10e-02	1.23e-02	-6.24e-03	9.69e-03	-6.22e-03	2.86e-02	-1.17e-02
1.87e-02	1.40e-02	-6.98e-03	1.09e-02	2.66e-03	8.60e-03	-1.53e-02	2.55e-02	1.64e-02
2.34e-03	1.28e-02	6.39e-03	9.89e-03	4.89e-03	7.82e-03	-5.16e-03	2.31e-02	1.27e-02
-1.56e-02	1.22e-02	-2.21e-03	9.46e-03	-7.24e-03	7.48e-03	-1.08e-02	2.21e-02	-6.71e-03
-7.76e-04	1.22e-02	-4.34e-03	9.49e-03	-3.02e-03	7.49e-03	1.15e-02	2.21e-02	8.27e-03
1.77e-02	1.40e-02	1.45e-02	1.09e-02	1.57e-02	8.63e-03	3.06e-02	2.53e-02	-5.44e-04
-2.99e-02	1.67e-02	-1.30e-02	1.28e-02	-1.94e-02	1.02e-02	-9.67e-03	3.00e-02	-2.18e-02
-3.66e-02	3.81e-02	-4.71e-03	2.93e-02	-1.67e-02	2.32e-02	2.77e-02	6.74e-02	2.37e-02
2.70e-02	1.48e-02	-1.26e-02	1.16e-02	2.71e-03	9.14e-03	3.99e-02	2.67e-02	-1.32e-02
-1.42e-02	1.28e-02	-2.59e-03	9.92e-03	-7.05e-03	7.84e-03	-8.45e-03	2.32e-02	-2.93e-03
-4.54e-02	2.97e-02	-5.52e-03	2.27e-02	-2.07e-02	1.80e-02	-7.62e-02	5.45e-02	3.83e-03
1.53e-02	2.33e-02	-2.28e-02	1.84e-02	-8.32e-03	1.44e-02	5.30e-02	4.16e-02	-1.12e-03
8.74e-04	1.26e-02	-5.98e-03	9.77e-03	-3.37e-03	7.72e-03	3.09e-03	2.28e-02	-2.03e-02
-4.02e-02	1.30e-02	-4.77e-02	1.01e-02	-4.49e-02	7.99e-03	-1.84e-02	2.36e-02	-4.91e-02
4.54e-03	1.29e-02	-2.14e-02	1.00e-02	-1.16e-02	7.90e-03	-1.05e-02	2.34e-02	-2.14e-02
5.62e-03	1.29e-02	-3.96e-03	1.00e-02	-6.53e-04	7.90e-03	-2.49e-02	2.34e-02	-1.43e-03
-2.05e-02	1.22e-02	-1.18e-02	9.49e-03	-1.51e-02	7.51e-03	-5.90e-03	2.22e-02	-1.29e-02
2.03e-02	1.23e-02	3.10e-02	9.54e-03	2.69e-02	7.54e-03	1.76e-02	2.23e-02	1.42e-02
-6.47e-03	1.55e-02	-9.68e-03	1.20e-02	-8.76e-03	9.47e-03	-4.32e-02	2.83e-02	-1.86e-02
4.55e-03	1.37e-02	2.93e-02	1.07e-02	1.99e-02	8.42e-03	-4.10e-03	2.48e-02	2.59e-02
-9.64e-03	1.25e-02	-1.01e-02	9.69e-03	-9.75e-03	7.66e-03	-9.71e-04	2.26e-02	-1.68e-02
-3.80e-02	1.26e-02	-2.81e-02	9.78e-03	-3.18e-02	7.73e-03	-7.03e-02	2.27e-02	-2.93e-02
1.08e-04	1.40e-02	2.78e-03	1.09e-02	1.60e-03	8.58e-03	-6.73e-03	2.52e-02	4.64e-03
-7.08e-03	1.22e-02	-1.40e-03	9.48e-03	-3.57e-03	7.49e-03	-1.68e-02	2.21e-02	9.38e-03
2.29e-02	1.22e-02	1.10e-02	9.47e-03	1.56e-02	7.49e-03	5.18e-02	2.22e-02	3.85e-03
1.32e-02	1.22e-02	1.28e-02	9.44e-03	1.30e-02	7.46e-03	-2.75e-02	2.21e-02	1.61e-02
5.45e-03	1.22e-02	2.22e-02	9.46e-03	1.58e-02	7.48e-03	1.56e-02	2.21e-02	2.35e-02
1.61e-03	1.22e-02	8.00e-03	9.44e-03	5.64e-03	7.46e-03	-2.99e-02	2.21e-02	9.86e-03
-9.06e-03	1.65e-02	-2.73e-02	1.28e-02	-2.05e-02	1.01e-02	8.26e-03	3.01e-02	-3.16e-02
1.04e-02	1.25e-02	1.32e-02	9.71e-03	1.20e-02	7.68e-03	-2.24e-02	2.27e-02	8.93e-03
-2.42e-02	1.26e-02	7.24e-03	9.80e-03	-4.73e-03	7.74e-03	-2.73e-02	2.28e-02	1.58e-03
1.06e-02	1.44e-02	-1.59e-02	1.12e-02	-5.84e-03	8.85e-03	-2.66e-02	2.63e-02	-1.61e-02
3.00e-02	1.45e-02	2.87e-02	1.13e-02	2.94e-02	8.92e-03	5.26e-02	2.61e-02	3.65e-02
-1.26e-02	1.33e-02	-2.34e-02	1.03e-02	-1.94e-02	8.18e-03	-6.38e-03	2.42e-02	-2.13e-02
1.36e-03	1.22e-02	1.08e-02	9.44e-03	7.39e-03	7.46e-03	-1.81e-02	2.20e-02	4.15e-03
1.05e-02	1.22e-02	-1.43e-02	9.51e-03	-5.12e-03	7.50e-03	2.16e-02	2.21e-02	-9.12e-04
-1.69e-02	2.90e-02	1.67e-02	2.23e-02	3.53e-03	1.77e-02	7.92e-02	5.05e-02	-3.82e-03

-6.02e-03	1.63e-02	-2.25e-02	1.26e-02	-1.62e-02	9.96e-03	-1.81e-02	2.94e-02	-2.54e-02
1.25e-02	1.24e-02	1.03e-02	9.65e-03	1.11e-02	7.62e-03	1.22e-02	2.25e-02	-6.43e-03
3.57e-03	1.22e-02	2.28e-03	9.43e-03	2.96e-03	7.46e-03	3.04e-02	2.21e-02	7.49e-03
-6.97e-03	1.33e-02	-6.24e-04	1.04e-02	-2.99e-03	8.18e-03	-3.71e-02	2.40e-02	8.38e-04
-7.61e-03	1.34e-02	-1.89e-02	1.04e-02	-1.44e-02	8.22e-03	-4.33e-02	2.44e-02	9.93e-03
-3.30e-02	1.52e-02	-1.76e-02	1.18e-02	-2.32e-02	9.33e-03	-4.16e-02	2.77e-02	1.07e-02
4.07e-03	2.13e-02	2.23e-02	1.64e-02	1.55e-02	1.30e-02	-1.87e-02	3.89e-02	4.74e-02
2.66e-02	1.45e-02	1.94e-02	1.13e-02	2.22e-02	8.92e-03	3.46e-02	2.62e-02	1.27e-02
1.08e-02	1.23e-02	1.39e-02	9.53e-03	1.30e-02	7.53e-03	-8.28e-03	2.22e-02	-8.28e-03
1.11e-02	1.22e-02	1.36e-02	9.53e-03	1.27e-02	7.52e-03	2.45e-02	2.22e-02	4.01e-03
2.13e-02	1.78e-02	-1.21e-02	1.39e-02	3.86e-04	1.10e-02	-4.61e-03	3.25e-02	-2.27e-02
2.84e-02	1.72e-02	-5.73e-03	1.34e-02	6.94e-03	1.06e-02	-1.77e-02	3.15e-02	5.00e-03
3.99e-03	1.26e-02	7.60e-03	9.77e-03	6.37e-03	7.72e-03	-4.44e-02	2.29e-02	-8.07e-04
-7.35e-03	1.25e-02	4.05e-03	9.74e-03	-1.89e-04	7.68e-03	-1.29e-02	2.27e-02	1.70e-02
3.00e-02	1.22e-02	2.46e-03	9.46e-03	1.28e-02	7.48e-03	3.35e-02	2.21e-02	1.25e-02
2.88e-02	1.28e-02	2.32e-03	1.00e-02	1.22e-02	7.89e-03	5.65e-02	2.31e-02	4.98e-03
-2.16e-02	1.38e-02	-4.44e-02	1.08e-02	-3.57e-02	8.52e-03	-4.07e-02	2.52e-02	-5.94e-02
-2.35e-02	1.23e-02	8.51e-04	9.60e-03	-8.36e-03	7.58e-03	-3.40e-02	2.23e-02	-3.65e-04
-9.34e-03	1.66e-02	2.68e-03	1.28e-02	-1.68e-03	1.01e-02	2.19e-02	2.98e-02	3.93e-03
-7.74e-03	2.16e-02	1.75e-02	1.66e-02	8.08e-03	1.32e-02	8.21e-04	3.88e-02	1.92e-02
-3.61e-03	1.23e-02	-1.31e-02	9.54e-03	-9.44e-03	7.54e-03	-3.02e-03	2.23e-02	-1.00e-03
2.38e-02	1.22e-02	1.77e-02	9.50e-03	1.99e-02	7.49e-03	2.93e-02	2.21e-02	1.15e-02
1.53e-02	1.42e-02	1.25e-02	1.11e-02	1.33e-02	8.74e-03	-2.01e-02	2.59e-02	1.85e-02
4.69e-03	1.25e-02	1.88e-02	9.67e-03	1.34e-02	7.65e-03	-2.77e-03	2.26e-02	9.82e-03
-5.45e-02	2.89e-02	-4.01e-02	2.23e-02	-4.61e-02	1.77e-02	-5.58e-02	5.28e-02	-7.75e-02
-3.78e-02	2.83e-02	2.23e-02	2.15e-02	-1.05e-04	1.71e-02	-9.18e-02	5.25e-02	-1.69e-02
-1.35e-04	1.22e-02	1.36e-02	9.54e-03	8.42e-03	7.53e-03	1.93e-02	2.22e-02	5.58e-03
-5.76e-03	1.26e-02	-1.53e-02	9.78e-03	-1.17e-02	7.74e-03	-2.21e-02	2.28e-02	-1.72e-02
3.56e-02	3.99e-02	-1.95e-02	3.18e-02	1.78e-03	2.49e-02	-9.76e-03	7.33e-02	-5.21e-02
-3.63e-02	1.78e-02	-4.16e-03	1.37e-02	-1.59e-02	1.09e-02	-2.81e-02	3.23e-02	1.76e-02
1.77e-02	2.11e-02	7.67e-03	1.64e-02	1.16e-02	1.30e-02	1.73e-02	3.82e-02	1.72e-02
6.39e-03	1.74e-02	2.39e-02	1.35e-02	1.75e-02	1.07e-02	-1.43e-02	3.18e-02	2.14e-02
-2.10e-02	1.55e-02	-8.09e-03	1.20e-02	-1.34e-02	9.52e-03	8.74e-03	2.80e-02	-1.41e-02
-9.28e-03	1.46e-02	2.56e-03	1.13e-02	-1.95e-03	8.92e-03	-4.72e-03	2.64e-02	1.86e-02
-1.15e-02	1.23e-02	9.51e-03	9.50e-03	1.68e-03	7.52e-03	2.73e-02	2.22e-02	1.47e-03
-2.25e-02	1.28e-02	-2.94e-03	9.90e-03	-1.02e-02	7.83e-03	1.12e-02	2.31e-02	-1.36e-02
5.52e-03	1.34e-02	-1.57e-03	1.04e-02	1.19e-03	8.25e-03	-2.32e-02	2.42e-02	-9.02e-03
5.67e-03	1.21e-02	2.81e-02	9.44e-03	1.95e-02	7.45e-03	1.74e-02	2.20e-02	3.35e-02
-1.77e-02	1.21e-02	-1.97e-02	9.43e-03	-1.91e-02	7.45e-03	-1.87e-02	2.20e-02	-2.10e-02
-2.05e-04	1.22e-02	3.08e-03	9.49e-03	2.18e-03	7.50e-03	-3.74e-02	2.22e-02	2.03e-02
2.13e-03	1.24e-02	-1.73e-02	9.63e-03	-1.02e-02	7.61e-03	8.95e-04	2.24e-02	-1.25e-02
-4.04e-02	1.24e-02	-2.65e-02	9.58e-03	-3.15e-02	7.58e-03	-2.87e-02	2.24e-02	-3.53e-02
-1.95e-02	1.95e-02	-4.58e-03	1.53e-02	-1.06e-02	1.20e-02	-4.51e-03	3.54e-02	1.00e-02
1.24e-02	1.29e-02	-8.83e-03	1.00e-02	-8.77e-04	7.93e-03	4.05e-02	2.33e-02	5.18e-03
1.92e-02	1.61e-02	2.35e-02	1.25e-02	2.24e-02	9.88e-03	1.68e-02	2.92e-02	2.17e-03
-2.95e-02	1.61e-02	-1.35e-02	1.24e-02	-1.95e-02	9.81e-03	-5.25e-02	2.94e-02	-7.94e-03
-4.11e-02	1.23e-02	-2.37e-03	9.57e-03	-1.70e-02	7.55e-03	-5.10e-02	2.22e-02	-7.43e-03

-2.66e-02	1.24e-02	-2.44e-02	9.55e-03	-2.53e-02	7.56e-03	-2.49e-02	2.24e-02	-1.93e-02
-1.59e-02	1.25e-02	1.27e-03	9.67e-03	-5.02e-03	7.64e-03	-9.81e-03	2.26e-02	3.10e-03
1.56e-02	1.21e-02	6.72e-03	9.41e-03	1.01e-02	7.44e-03	6.92e-03	2.19e-02	-4.04e-03
1.85e-03	1.32e-02	1.35e-02	1.02e-02	8.94e-03	8.06e-03	7.04e-04	2.38e-02	-6.70e-04
-1.75e-02	1.23e-02	-1.24e-02	9.54e-03	-1.42e-02	7.54e-03	9.75e-03	2.24e-02	3.90e-04
-1.19e-03	1.23e-02	-1.41e-02	9.55e-03	-9.13e-03	7.54e-03	9.26e-04	2.22e-02	-1.17e-02
1.19e-02	1.24e-02	1.90e-02	9.66e-03	1.63e-02	7.63e-03	2.87e-02	2.25e-02	1.68e-02
2.96e-02	1.48e-02	2.24e-02	1.15e-02	2.51e-02	9.09e-03	9.82e-03	2.69e-02	1.90e-02
-7.14e-03	2.30e-02	-1.45e-02	1.79e-02	-1.19e-02	1.41e-02	5.76e-02	4.08e-02	-3.90e-02
3.78e-02	1.89e-02	1.39e-02	1.49e-02	2.31e-02	1.17e-02	5.33e-02	3.40e-02	-4.75e-03
6.00e-04	1.96e-02	-3.45e-02	1.53e-02	-2.11e-02	1.21e-02	5.00e-03	3.54e-02	-4.53e-02
-1.33e-02	1.23e-02	-5.39e-04	9.59e-03	-5.40e-03	7.57e-03	5.34e-03	2.23e-02	-7.57e-03
4.06e-02	1.22e-02	3.99e-02	9.50e-03	4.00e-02	7.51e-03	3.25e-02	2.21e-02	4.75e-02
-2.07e-02	1.25e-02	-1.73e-02	9.70e-03	-1.84e-02	7.67e-03	2.50e-03	2.27e-02	-1.62e-02
2.31e-02	1.79e-02	9.73e-03	1.40e-02	1.46e-02	1.10e-02	-1.63e-02	3.29e-02	3.58e-02
-1.28e-02	1.65e-02	-1.30e-02	1.28e-02	-1.28e-02	1.01e-02	1.69e-02	2.95e-02	-3.33e-03
1.72e-02	1.28e-02	-8.48e-03	1.00e-02	1.29e-03	7.91e-03	2.66e-02	2.32e-02	-1.54e-02
-3.42e-02	1.72e-02	2.26e-02	1.31e-02	1.49e-03	1.04e-02	-5.15e-02	3.13e-02	-6.25e-03
-9.62e-03	1.76e-02	-2.69e-03	1.37e-02	-5.40e-03	1.08e-02	6.29e-02	3.12e-02	-3.15e-04
-2.38e-02	1.48e-02	-2.25e-02	1.14e-02	-2.31e-02	9.05e-03	5.84e-03	2.66e-02	-1.46e-02
-4.36e-02	1.67e-02	-6.33e-03	1.28e-02	-2.02e-02	1.02e-02	-2.90e-02	3.03e-02	-2.28e-02
-6.95e-04	1.90e-02	-3.17e-02	1.49e-02	-1.99e-02	1.17e-02	-4.17e-02	3.50e-02	-1.88e-02
-2.30e-02	1.49e-02	7.98e-03	1.16e-02	-3.66e-03	9.19e-03	-8.40e-03	2.71e-02	-5.08e-04
-2.76e-02	1.21e-02	1.42e-03	9.42e-03	-9.66e-03	7.44e-03	-1.89e-02	2.20e-02	1.41e-02
2.19e-02	1.23e-02	1.93e-02	9.52e-03	2.01e-02	7.52e-03	4.88e-02	2.22e-02	7.20e-03
1.44e-02	1.23e-02	3.25e-02	9.49e-03	2.57e-02	7.51e-03	4.25e-02	2.22e-02	4.84e-02
6.32e-03	2.32e-02	2.27e-02	1.80e-02	1.72e-02	1.42e-02	2.27e-02	4.18e-02	3.28e-02
1.13e-02	1.32e-02	2.66e-03	1.03e-02	5.88e-03	8.11e-03	2.13e-03	2.40e-02	7.97e-03
-1.23e-02	1.46e-02	9.22e-04	1.13e-02	-4.18e-03	8.95e-03	-4.77e-02	2.67e-02	9.00e-03
-8.22e-02	3.08e-02	-1.11e-02	2.30e-02	-3.73e-02	1.84e-02	-7.55e-02	5.61e-02	-6.49e-03
-2.27e-03	1.25e-02	-2.34e-02	9.72e-03	-1.55e-02	7.69e-03	2.94e-03	2.27e-02	-2.69e-02
-2.76e-02	1.27e-02	-2.30e-02	9.88e-03	-2.49e-02	7.81e-03	-4.32e-02	2.31e-02	-1.79e-02
-3.41e-02	1.56e-02	-3.11e-02	1.21e-02	-3.24e-02	9.54e-03	4.39e-04	2.79e-02	-3.83e-02
2.30e-03	1.30e-02	4.29e-03	1.01e-02	3.55e-03	7.96e-03	-8.70e-03	2.35e-02	1.07e-02
-4.05e-04	1.47e-02	8.74e-03	1.14e-02	5.04e-03	9.02e-03	7.49e-03	2.67e-02	3.85e-03
-3.02e-02	1.69e-02	-1.97e-02	1.31e-02	-2.39e-02	1.04e-02	-9.94e-02	3.15e-02	-1.41e-02
2.29e-03	1.22e-02	1.35e-02	9.47e-03	9.21e-03	7.48e-03	1.34e-02	2.21e-02	5.10e-03
3.37e-03	1.81e-02	2.72e-02	1.41e-02	1.85e-02	1.11e-02	-4.03e-02	3.33e-02	1.10e-02
-1.60e-02	1.21e-02	1.75e-03	9.43e-03	-4.64e-03	7.46e-03	1.21e-04	2.20e-02	5.52e-03
-2.18e-02	1.28e-02	-2.91e-02	9.89e-03	-2.65e-02	7.82e-03	-5.03e-02	2.32e-02	-2.62e-02
3.10e-03	1.40e-02	1.33e-02	1.09e-02	9.36e-03	8.60e-03	2.55e-03	2.53e-02	2.05e-02
-4.67e-02	1.61e-02	-2.74e-02	1.25e-02	-3.46e-02	9.89e-03	-3.01e-02	2.91e-02	-1.27e-02
-2.58e-02	1.22e-02	-7.18e-03	9.49e-03	-1.41e-02	7.51e-03	-4.53e-03	2.22e-02	2.19e-03
-1.83e-03	1.47e-02	2.06e-02	1.14e-02	1.22e-02	9.03e-03	-6.42e-03	2.66e-02	2.16e-02
1.39e-03	1.32e-02	-2.86e-03	1.02e-02	-1.28e-03	8.09e-03	-1.82e-02	2.39e-02	1.60e-03
-1.46e-02	1.23e-02	-2.41e-02	9.54e-03	-2.08e-02	7.54e-03	-1.00e-02	2.23e-02	-1.60e-02
2.61e-02	1.24e-02	-1.24e-02	9.60e-03	1.91e-03	7.59e-03	-1.54e-02	2.24e-02	1.40e-02

-1.08e-02	1.35e-02	-5.43e-03	1.05e-02	-7.68e-03	8.30e-03	-6.48e-03	2.46e-02	-5.14e-03
2.80e-02	1.26e-02	1.47e-02	9.80e-03	1.98e-02	7.75e-03	4.55e-02	2.29e-02	4.12e-02
3.97e-03	1.32e-02	2.69e-02	1.04e-02	1.82e-02	8.17e-03	-6.61e-03	2.39e-02	3.41e-02
7.20e-03	1.30e-02	-1.50e-02	1.01e-02	-6.39e-03	7.97e-03	1.72e-02	2.34e-02	-1.15e-02
1.58e-02	1.29e-02	2.67e-02	1.00e-02	2.28e-02	7.93e-03	-3.43e-02	2.36e-02	4.74e-02
2.38e-03	1.37e-02	-4.74e-03	1.06e-02	-1.84e-03	8.40e-03	-1.52e-02	2.48e-02	-1.14e-02
-2.26e-03	1.39e-02	-9.14e-03	1.08e-02	-6.59e-03	8.55e-03	-4.56e-02	2.50e-02	-1.71e-02
1.12e-03	1.23e-02	2.02e-02	9.57e-03	1.30e-02	7.56e-03	2.44e-02	2.23e-02	3.68e-02
2.03e-02	1.40e-02	1.74e-02	1.09e-02	1.83e-02	8.60e-03	7.55e-02	2.51e-02	2.02e-02
-6.97e-03	1.35e-02	-1.64e-03	1.05e-02	-3.71e-03	8.31e-03	6.32e-03	2.46e-02	-3.22e-03
-6.07e-03	1.24e-02	1.41e-03	9.63e-03	-1.59e-03	7.61e-03	-3.16e-02	2.26e-02	-1.87e-02
-2.83e-02	1.34e-02	-3.90e-03	1.04e-02	-1.30e-02	8.23e-03	-2.11e-02	2.43e-02	-2.24e-02
-6.36e-04	1.22e-02	-1.21e-02	9.51e-03	-7.92e-03	7.51e-03	-1.72e-03	2.21e-02	-8.30e-03
-1.17e-02	1.22e-02	-1.88e-02	9.45e-03	-1.63e-02	7.48e-03	-2.43e-02	2.21e-02	-2.37e-03
-6.30e-03	1.37e-02	-1.44e-02	1.07e-02	-1.13e-02	8.42e-03	-2.05e-02	2.49e-02	2.05e-03
2.07e-02	1.31e-02	9.78e-03	1.02e-02	1.37e-02	8.05e-03	4.01e-02	2.39e-02	2.39e-05
9.00e-03	1.30e-02	2.31e-03	1.01e-02	4.90e-03	7.99e-03	-1.73e-02	2.35e-02	-6.66e-04
-6.66e-03	1.25e-02	8.99e-03	9.75e-03	3.04e-03	7.69e-03	-4.05e-03	2.27e-02	1.55e-02
2.04e-02	1.22e-02	2.97e-02	9.48e-03	2.61e-02	7.49e-03	1.16e-02	2.21e-02	1.16e-02
-9.94e-03	1.21e-02	-4.67e-03	9.42e-03	-6.49e-03	7.44e-03	-1.29e-02	2.20e-02	-1.01e-02
-2.81e-02	1.32e-02	-6.92e-03	1.03e-02	-1.51e-02	8.11e-03	-6.46e-02	2.38e-02	-1.00e-02
-2.69e-02	2.29e-02	5.79e-04	1.77e-02	-9.82e-03	1.40e-02	-6.17e-02	4.21e-02	4.90e-03
-1.03e-02	1.37e-02	9.40e-03	1.06e-02	1.98e-03	8.39e-03	-1.62e-02	2.47e-02	-8.13e-03
-1.68e-02	1.23e-02	-1.19e-02	9.48e-03	-1.38e-02	7.51e-03	2.54e-03	2.22e-02	4.78e-03
-4.25e-03	1.25e-02	6.42e-04	9.66e-03	-1.16e-03	7.64e-03	7.75e-04	2.26e-02	7.11e-03
-1.42e-03	1.29e-02	5.56e-03	1.01e-02	2.87e-03	7.95e-03	1.98e-02	2.35e-02	-1.08e-02
-7.89e-02	6.45e-02	6.07e-02	4.65e-02	1.12e-02	3.76e-02	5.41e-02	1.11e-01	1.31e-02
5.52e-03	1.21e-02	6.82e-03	9.42e-03	6.51e-03	7.44e-03	4.01e-04	2.20e-02	1.31e-02
2.19e-02	1.35e-02	-3.81e-03	1.05e-02	5.81e-03	8.31e-03	4.40e-02	2.44e-02	-2.06e-02
6.81e-03	1.23e-02	6.92e-03	9.59e-03	6.87e-03	7.58e-03	-4.55e-03	2.24e-02	8.52e-03
-3.75e-02	1.44e-02	1.48e-03	1.13e-02	-1.34e-02	8.93e-03	-8.34e-02	2.59e-02	-3.34e-03
-4.41e-03	1.23e-02	-4.05e-03	9.53e-03	-4.23e-03	7.54e-03	-4.21e-03	2.23e-02	-1.77e-03

MI male SE	I overall Be	/I overall S	R female B	TR female S	TR male Be	STR male S	R overall B	TR overall S
1.36e-02	-6.49e-03	1.17e-02	-6.53e-03	1.93e-02	-6.33e-03	1.66e-02	-6.63e-03	1.26e-02
1.33e-02	3.17e-03	1.14e-02	6.85e-02	1.89e-02	8.32e-04	1.62e-02	2.94e-02	1.23e-02
1.36e-02	8.43e-04	1.17e-02	8.83e-03	1.93e-02	1.50e-02	1.66e-02	1.25e-02	1.26e-02
1.37e-02	-2.23e-02	1.17e-02	4.40e-03	1.93e-02	-2.36e-02	1.66e-02	-1.18e-02	1.26e-02
1.33e-02	2.66e-02	1.14e-02	4.25e-03	1.88e-02	2.61e-03	1.62e-02	3.11e-03	1.22e-02
1.33e-02	2.34e-02	1.14e-02	1.08e-02	1.88e-02	6.85e-03	1.62e-02	8.55e-03	1.23e-02
1.42e-02	1.13e-02	1.22e-02	8.16e-03	2.00e-02	3.14e-02	1.73e-02	2.13e-02	1.31e-02
1.33e-02	-1.82e-03	1.14e-02	1.09e-02	1.88e-02	-1.03e-02	1.61e-02	-1.29e-03	1.22e-02
1.43e-02	-1.02e-02	1.23e-02	-1.18e-02	2.02e-02	1.78e-03	1.74e-02	-3.87e-03	1.32e-02
1.37e-02	3.00e-03	1.17e-02	-1.53e-02	1.93e-02	1.00e-02	1.67e-02	-6.97e-04	1.26e-02
1.41e-02	2.80e-02	1.21e-02	1.87e-02	2.01e-02	2.32e-02	1.72e-02	2.12e-02	1.30e-02
1.36e-02	-1.43e-02	1.16e-02	-1.64e-02	1.92e-02	-3.27e-02	1.65e-02	-2.57e-02	1.25e-02
1.62e-02	1.74e-02	1.39e-02	-2.11e-02	2.32e-02	-4.05e-03	1.98e-02	-1.12e-02	1.51e-02
1.35e-02	4.14e-03	1.15e-02	-1.15e-03	1.91e-02	-3.13e-02	1.63e-02	-1.84e-02	1.24e-02
2.85e-02	1.72e-02	2.42e-02	-4.16e-03	4.02e-02	-7.59e-02	3.57e-02	-4.51e-02	2.67e-02
1.38e-02	-1.80e-02	1.18e-02	-4.90e-03	1.96e-02	1.02e-02	1.69e-02	3.92e-03	1.28e-02
1.40e-02	1.35e-02	1.20e-02	2.93e-02	1.98e-02	-8.69e-03	1.71e-02	7.48e-03	1.29e-02
1.69e-02	5.18e-02	1.45e-02	2.91e-04	2.44e-02	-2.06e-02	2.10e-02	-1.16e-02	1.59e-02
1.36e-02	-2.12e-02	1.16e-02	4.17e-02	1.92e-02	3.32e-03	1.65e-02	1.96e-02	1.25e-02
1.57e-02	-1.25e-02	1.34e-02	-4.10e-03	2.21e-02	-1.19e-02	1.91e-02	-8.71e-03	1.45e-02
1.69e-02	1.65e-02	1.45e-02	-2.13e-02	2.43e-02	1.30e-02	2.06e-02	-1.28e-03	1.57e-02
1.36e-02	-3.43e-04	1.16e-02	-1.89e-02	1.92e-02	-2.11e-03	1.65e-02	-9.24e-03	1.25e-02
1.45e-02	1.60e-02	1.24e-02	4.23e-02	2.04e-02	3.71e-03	1.77e-02	2.01e-02	1.33e-02
1.36e-02	-1.35e-02	1.17e-02	-1.76e-02	1.92e-02	2.41e-03	1.66e-02	-6.01e-03	1.26e-02
1.35e-02	-9.64e-03	1.15e-02	3.17e-02	1.90e-02	1.53e-02	1.64e-02	2.25e-02	1.24e-02
1.69e-02	7.34e-03	1.45e-02	3.41e-02	2.38e-02	-1.31e-02	2.06e-02	6.78e-03	1.56e-02
1.80e-02	-1.10e-02	1.54e-02	-1.29e-02	2.56e-02	-3.25e-02	2.20e-02	-2.38e-02	1.67e-02
1.34e-02	6.40e-03	1.14e-02	-8.61e-03	1.88e-02	-1.03e-02	1.62e-02	-9.58e-03	1.23e-02
1.38e-02	1.44e-02	1.18e-02	-4.30e-02	1.97e-02	-1.38e-02	1.68e-02	-2.62e-02	1.28e-02
1.44e-02	1.56e-02	1.23e-02	1.04e-02	2.04e-02	-3.11e-03	1.75e-02	2.69e-03	1.33e-02
2.23e-02	-5.67e-02	1.91e-02	-3.29e-02	3.14e-02	-7.44e-02	2.72e-02	-5.68e-02	2.05e-02
1.71e-02	2.35e-02	1.46e-02	-1.10e-02	2.44e-02	5.81e-02	2.06e-02	2.91e-02	1.57e-02
1.36e-02	-1.45e-02	1.17e-02	-2.63e-02	1.93e-02	-2.14e-02	1.66e-02	-2.34e-02	1.26e-02
1.78e-02	4.67e-02	1.52e-02	1.78e-02	2.53e-02	1.90e-02	2.18e-02	1.84e-02	1.65e-02
1.35e-02	-2.20e-02	1.16e-02	-3.52e-03	1.91e-02	-1.78e-02	1.64e-02	-1.16e-02	1.25e-02
1.33e-02	1.35e-02	1.14e-02	-2.86e-02	1.88e-02	-1.30e-02	1.62e-02	-1.97e-02	1.23e-02
2.12e-02	-4.85e-03	1.81e-02	5.98e-02	2.92e-02	3.07e-02	2.54e-02	4.31e-02	1.92e-02
2.80e-02	-1.35e-03	2.39e-02	5.86e-03	3.92e-02	7.01e-03	3.39e-02	6.64e-03	2.56e-02
2.57e-02	-5.79e-02	2.19e-02	-4.22e-02	3.63e-02	3.21e-02	3.00e-02	1.39e-03	2.31e-02
1.39e-02	1.48e-02	1.19e-02	1.31e-02	1.97e-02	-2.84e-03	1.69e-02	4.19e-03	1.28e-02
1.76e-02	-2.46e-04	1.51e-02	5.33e-02	2.46e-02	-7.47e-03	2.15e-02	1.87e-02	1.62e-02
1.33e-02	5.02e-03	1.14e-02	3.56e-02	1.88e-02	1.56e-02	1.61e-02	2.42e-02	1.22e-02
1.58e-02	-2.61e-02	1.35e-02	-3.86e-02	2.24e-02	-3.35e-02	1.92e-02	-3.56e-02	1.46e-02
1.35e-02	2.92e-03	1.15e-02	2.12e-02	1.90e-02	-1.38e-02	1.63e-02	8.76e-04	1.24e-02
2.08e-02	6.52e-03	1.79e-02	3.12e-02	2.92e-02	3.22e-02	2.52e-02	3.16e-02	1.91e-02
1.42e-02	-1.55e-02	1.22e-02	3.06e-03	2.00e-02	-3.14e-02	1.73e-02	-1.68e-02	1.31e-02

1.33e-02	1.12e-02	1.14e-02	-2.02e-02	1.89e-02	-7.53e-03	1.62e-02	-1.28e-02	1.23e-02
2.13e-02	3.87e-02	1.83e-02	-5.06e-02	3.12e-02	-4.31e-03	2.64e-02	-2.39e-02	2.01e-02
1.71e-02	-5.30e-02	1.46e-02	1.05e-03	2.46e-02	2.32e-02	2.12e-02	1.40e-02	1.60e-02
1.53e-02	7.53e-03	1.31e-02	-4.16e-02	2.19e-02	-2.99e-02	1.87e-02	-3.49e-02	1.42e-02
1.33e-02	-2.55e-02	1.13e-02	8.65e-03	1.87e-02	-1.50e-02	1.61e-02	-4.83e-03	1.22e-02
1.54e-02	1.58e-02	1.31e-02	3.87e-02	2.16e-02	-2.12e-03	1.87e-02	1.54e-02	1.41e-02
2.09e-02	1.84e-02	1.78e-02	5.99e-02	2.90e-02	9.00e-03	2.54e-02	3.10e-02	1.91e-02
1.98e-02	-2.18e-02	1.70e-02	-1.47e-03	2.79e-02	1.96e-02	2.38e-02	1.07e-02	1.81e-02
1.51e-02	2.11e-02	1.29e-02	3.31e-03	2.15e-02	-5.03e-03	1.85e-02	-1.41e-03	1.40e-02
1.81e-02	-4.39e-03	1.55e-02	-9.05e-02	2.63e-02	2.97e-03	2.19e-02	-3.62e-02	1.68e-02
2.95e-02	-2.43e-02	2.55e-02	-6.85e-02	4.28e-02	-3.08e-02	3.63e-02	-4.68e-02	2.77e-02
1.40e-02	-9.69e-03	1.19e-02	-2.45e-02	1.96e-02	-1.50e-02	1.70e-02	-1.91e-02	1.28e-02
1.42e-02	-1.14e-02	1.22e-02	-2.69e-02	2.01e-02	-5.68e-03	1.73e-02	-1.46e-02	1.31e-02
1.73e-02	2.81e-02	1.48e-02	-3.88e-02	2.50e-02	1.56e-02	2.11e-02	-7.50e-03	1.61e-02
1.99e-02	9.03e-03	1.70e-02	2.39e-04	2.82e-02	-3.17e-03	2.42e-02	-1.83e-03	1.84e-02
1.46e-02	1.40e-02	1.25e-02	2.21e-03	2.07e-02	2.22e-05	1.78e-02	9.46e-04	1.35e-02
1.33e-02	6.84e-03	1.14e-02	7.73e-03	1.89e-02	3.82e-03	1.62e-02	5.52e-03	1.23e-02
1.50e-02	1.45e-02	1.29e-02	2.08e-02	2.13e-02	5.48e-03	1.83e-02	1.21e-02	1.39e-02
1.39e-02	1.42e-02	1.19e-02	2.72e-02	1.97e-02	2.59e-02	1.69e-02	2.64e-02	1.28e-02
1.33e-02	-4.25e-03	1.14e-02	-1.51e-02	1.88e-02	2.26e-02	1.62e-02	6.66e-03	1.23e-02
1.69e-02	1.34e-02	1.45e-02	-1.29e-02	2.39e-02	-9.47e-03	2.07e-02	-1.09e-02	1.56e-02
1.45e-02	4.06e-03	1.24e-02	9.12e-03	2.04e-02	2.67e-02	1.76e-02	1.92e-02	1.33e-02
2.04e-02	-3.45e-03	1.75e-02	-3.02e-02	2.94e-02	2.70e-02	2.46e-02	3.42e-03	1.88e-02
1.41e-02	1.23e-02	1.21e-02	2.94e-02	1.99e-02	-7.94e-03	1.71e-02	7.77e-03	1.30e-02
1.39e-02	-2.17e-02	1.20e-02	-1.82e-03	1.98e-02	-2.39e-03	1.70e-02	-2.26e-03	1.29e-02
1.51e-02	-4.79e-03	1.29e-02	1.57e-02	2.14e-02	4.41e-03	1.84e-02	9.13e-03	1.39e-02
2.95e-02	1.53e-02	2.52e-02	-2.33e-02	4.21e-02	-1.33e-02	3.61e-02	-1.78e-02	2.74e-02
1.87e-02	1.99e-02	1.60e-02	-1.16e-02	2.66e-02	5.62e-03	2.28e-02	-1.65e-03	1.73e-02
1.33e-02	1.50e-03	1.14e-02	1.88e-02	1.88e-02	-3.72e-03	1.61e-02	5.85e-03	1.23e-02
1.41e-02	-1.35e-02	1.20e-02	1.10e-02	2.00e-02	3.71e-03	1.71e-02	6.82e-03	1.30e-02
1.48e-02	5.39e-03	1.27e-02	-1.50e-02	2.11e-02	-2.73e-02	1.81e-02	-2.19e-02	1.37e-02
1.33e-02	6.63e-03	1.14e-02	1.11e-02	1.89e-02	1.25e-02	1.62e-02	1.19e-02	1.23e-02
1.59e-02	2.00e-02	1.37e-02	3.07e-02	2.25e-02	6.39e-03	1.94e-02	1.67e-02	1.47e-02
1.41e-02	2.43e-02	1.21e-02	4.24e-02	2.01e-02	6.53e-03	1.71e-02	2.16e-02	1.30e-02
1.42e-02	9.97e-03	1.21e-02	1.92e-02	2.01e-02	5.67e-04	1.72e-02	8.53e-03	1.31e-02
1.33e-02	2.74e-02	1.14e-02	-9.52e-03	1.88e-02	2.42e-02	1.61e-02	9.97e-03	1.22e-02
1.37e-02	-2.18e-02	1.17e-02	2.45e-03	1.93e-02	-1.29e-02	1.66e-02	-6.65e-03	1.26e-02
1.41e-02	6.04e-03	1.21e-02	-7.56e-04	2.00e-02	1.67e-02	1.71e-02	9.42e-03	1.30e-02
1.41e-02	-4.92e-03	1.21e-02	-3.11e-02	2.00e-02	-6.86e-04	1.72e-02	-1.37e-02	1.30e-02
1.33e-02	-3.31e-02	1.14e-02	4.65e-03	1.89e-02	-4.60e-03	1.62e-02	-6.81e-04	1.23e-02
1.60e-02	2.19e-02	1.37e-02	-1.59e-02	2.24e-02	8.54e-03	1.93e-02	-1.70e-03	1.46e-02
1.41e-02	6.57e-03	1.21e-02	1.11e-03	1.99e-02	-1.95e-02	1.71e-02	-1.09e-02	1.30e-02
1.53e-02	-1.44e-02	1.31e-02	2.86e-02	2.14e-02	-2.77e-02	1.86e-02	-3.61e-03	1.40e-02
1.37e-02	2.08e-02	1.17e-02	-3.35e-03	1.95e-02	-3.00e-02	1.67e-02	-1.87e-02	1.27e-02
1.55e-02	-9.31e-03	1.32e-02	7.05e-03	2.18e-02	2.99e-02	1.86e-02	2.02e-02	1.42e-02
1.33e-02	2.58e-02	1.14e-02	-1.63e-03	1.88e-02	7.44e-03	1.62e-02	3.69e-03	1.23e-02
1.53e-02	-1.47e-02	1.31e-02	3.39e-02	2.14e-02	3.60e-03	1.85e-02	1.66e-02	1.40e-02

1.36e-02	-1.95e-02	1.17e-02	7.80e-03	1.93e-02	-3.23e-02	1.66e-02	-1.52e-02	1.26e-02
1.34e-02	1.76e-02	1.15e-02	2.79e-02	1.89e-02	1.96e-02	1.63e-02	2.30e-02	1.23e-02
1.34e-02	2.10e-02	1.15e-02	4.63e-02	1.90e-02	8.86e-03	1.63e-02	2.49e-02	1.24e-02
1.34e-02	-7.03e-03	1.15e-02	1.90e-02	1.89e-02	1.61e-02	1.63e-02	1.73e-02	1.24e-02
2.21e-02	-2.56e-02	1.89e-02	2.33e-02	3.07e-02	-8.25e-03	2.67e-02	5.34e-03	2.02e-02
1.33e-02	1.33e-02	1.14e-02	-1.26e-02	1.90e-02	-2.63e-02	1.63e-02	-2.06e-02	1.23e-02
1.92e-02	1.53e-03	1.64e-02	4.16e-02	2.67e-02	2.64e-02	2.32e-02	3.30e-02	1.75e-02
1.33e-02	-2.53e-03	1.14e-02	-1.21e-02	1.89e-02	-1.11e-02	1.62e-02	-1.15e-02	1.23e-02
1.37e-02	9.74e-03	1.17e-02	-1.54e-02	1.94e-02	2.03e-02	1.66e-02	5.18e-03	1.26e-02
5.03e-02	-4.74e-02	4.27e-02	2.99e-02	6.86e-02	9.21e-03	5.90e-02	1.79e-02	4.47e-02
1.40e-02	-1.12e-02	1.20e-02	-1.44e-02	1.99e-02	1.24e-03	1.70e-02	-5.62e-03	1.29e-02
1.38e-02	5.11e-04	1.18e-02	-5.21e-03	1.95e-02	2.71e-02	1.67e-02	1.37e-02	1.27e-02
1.87e-02	-2.53e-03	1.61e-02	-9.56e-03	2.65e-02	-1.40e-02	2.27e-02	-1.21e-02	1.73e-02
1.34e-02	-1.13e-03	1.14e-02	-1.29e-02	1.89e-02	-5.47e-03	1.62e-02	-8.64e-03	1.23e-02
1.67e-02	-2.48e-02	1.44e-02	3.90e-02	2.33e-02	-7.08e-03	2.03e-02	1.27e-02	1.53e-02
1.51e-02	-3.23e-03	1.29e-02	1.53e-02	2.14e-02	-3.93e-02	1.82e-02	-1.62e-02	1.39e-02
1.68e-02	-3.50e-02	1.43e-02	2.44e-02	2.40e-02	-6.77e-03	2.04e-02	6.56e-03	1.56e-02
1.73e-02	-2.05e-02	1.48e-02	-1.68e-02	2.43e-02	-3.57e-03	2.09e-02	-9.39e-03	1.59e-02
2.46e-02	-3.10e-02	2.12e-02	-4.10e-02	3.52e-02	-1.17e-02	2.99e-02	-2.40e-02	2.28e-02
2.38e-02	3.80e-02	2.04e-02	-3.55e-03	3.43e-02	-5.28e-03	2.93e-02	-4.24e-03	2.23e-02
1.34e-02	4.44e-02	1.15e-02	7.57e-03	1.90e-02	7.98e-03	1.63e-02	7.98e-03	1.24e-02
1.33e-02	1.73e-03	1.14e-02	1.88e-02	1.88e-02	5.02e-03	1.62e-02	1.07e-02	1.23e-02
1.37e-02	1.31e-02	1.17e-02	2.62e-02	1.93e-02	-1.95e-02	1.67e-02	1.90e-04	1.26e-02
1.56e-02	2.04e-02	1.34e-02	3.11e-03	2.23e-02	-3.24e-02	1.92e-02	-1.73e-02	1.45e-02
1.34e-02	5.87e-03	1.15e-02	1.38e-02	1.90e-02	3.32e-03	1.63e-02	7.88e-03	1.24e-02
1.59e-02	-5.49e-03	1.36e-02	-1.78e-02	2.24e-02	-1.20e-02	1.93e-02	-1.44e-02	1.46e-02
1.36e-02	1.54e-02	1.16e-02	-8.38e-03	1.93e-02	-3.60e-02	1.66e-02	-2.42e-02	1.26e-02
1.34e-02	5.31e-03	1.15e-02	2.74e-02	1.90e-02	-8.08e-03	1.63e-02	6.94e-03	1.24e-02
1.34e-02	1.18e-02	1.15e-02	1.06e-02	1.91e-02	3.52e-02	1.63e-02	2.48e-02	1.24e-02
1.34e-02	1.70e-02	1.15e-02	2.61e-02	1.90e-02	-5.76e-03	1.63e-02	7.55e-03	1.24e-02
1.83e-02	-7.70e-03	1.56e-02	-3.58e-03	2.58e-02	-5.39e-03	2.22e-02	-4.84e-03	1.68e-02
1.33e-02	1.74e-02	1.14e-02	-1.05e-02	1.88e-02	1.27e-02	1.61e-02	2.62e-03	1.22e-02
1.33e-02	-2.58e-02	1.14e-02	1.19e-02	1.89e-02	-1.58e-02	1.62e-02	-4.12e-03	1.23e-02
1.36e-02	-1.25e-02	1.16e-02	-3.29e-02	1.92e-02	-7.33e-03	1.65e-02	-1.81e-02	1.25e-02
1.42e-02	-1.83e-02	1.22e-02	-4.68e-02	2.02e-02	-2.68e-02	1.73e-02	-3.51e-02	1.31e-02
1.97e-02	1.46e-02	1.67e-02	7.95e-02	2.70e-02	8.62e-03	2.38e-02	3.92e-02	1.78e-02
1.45e-02	-6.24e-03	1.24e-02	3.75e-02	2.06e-02	6.49e-03	1.76e-02	1.96e-02	1.34e-02
1.48e-02	-1.66e-04	1.27e-02	-2.27e-02	2.11e-02	-1.47e-02	1.81e-02	-1.79e-02	1.37e-02
1.36e-02	-3.15e-02	1.16e-02	-7.78e-03	1.92e-02	-2.61e-02	1.65e-02	-1.84e-02	1.25e-02
2.19e-02	4.25e-02	1.86e-02	5.44e-02	3.06e-02	1.15e-02	2.67e-02	3.00e-02	2.01e-02
1.38e-02	-3.80e-02	1.18e-02	1.02e-02	1.97e-02	-3.85e-02	1.68e-02	-1.80e-02	1.28e-02
1.73e-02	-9.43e-03	1.48e-02	-8.84e-03	2.43e-02	5.19e-03	2.09e-02	-5.54e-04	1.58e-02
1.73e-02	1.65e-02	1.48e-02	3.10e-02	2.44e-02	-2.09e-02	2.11e-02	1.26e-03	1.60e-02
1.40e-02	-9.57e-03	1.20e-02	1.03e-02	1.98e-02	-1.62e-02	1.69e-02	-4.98e-03	1.29e-02
1.87e-02	4.76e-02	1.60e-02	-2.58e-02	2.70e-02	-3.17e-02	2.32e-02	-2.93e-02	1.76e-02
1.56e-02	-2.15e-02	1.33e-02	-1.55e-02	2.20e-02	-1.97e-02	1.89e-02	-1.80e-02	1.43e-02
1.39e-02	-2.47e-02	1.19e-02	2.47e-02	1.97e-02	-7.48e-03	1.69e-02	5.70e-03	1.28e-02

1.54e-02	2.51e-02	1.31e-02	-1.18e-02	2.18e-02	1.29e-02	1.86e-02	2.58e-03	1.42e-02
1.37e-02	1.65e-02	1.17e-02	3.08e-03	1.93e-02	-3.58e-02	1.66e-02	-1.93e-02	1.26e-02
1.54e-02	6.80e-02	1.32e-02	1.41e-02	2.16e-02	1.37e-02	1.85e-02	1.39e-02	1.41e-02
1.35e-02	-1.51e-02	1.16e-02	1.93e-03	1.91e-02	5.44e-03	1.64e-02	3.89e-03	1.25e-02
1.40e-02	-3.12e-03	1.20e-02	-7.80e-03	1.97e-02	1.52e-03	1.70e-02	-2.57e-03	1.29e-02
1.76e-02	-1.36e-02	1.51e-02	-5.30e-02	2.52e-02	1.74e-04	2.13e-02	-2.22e-02	1.63e-02
1.33e-02	7.89e-03	1.14e-02	6.81e-03	1.88e-02	1.74e-02	1.62e-02	1.29e-02	1.22e-02
1.35e-02	3.28e-02	1.16e-02	5.21e-03	1.92e-02	1.85e-02	1.65e-02	1.28e-02	1.25e-02
1.41e-02	-8.13e-03	1.21e-02	-3.99e-02	2.02e-02	-1.77e-02	1.72e-02	-2.70e-02	1.31e-02
1.33e-02	-9.45e-03	1.14e-02	2.35e-02	1.88e-02	-2.03e-03	1.62e-02	8.74e-03	1.23e-02
1.73e-02	-1.03e-02	1.48e-02	4.86e-03	2.43e-02	-3.01e-02	2.12e-02	-1.50e-02	1.60e-02
1.53e-02	7.93e-03	1.31e-02	3.43e-02	2.16e-02	-5.81e-03	1.86e-02	1.15e-02	1.41e-02
1.39e-02	7.95e-03	1.19e-02	6.85e-03	1.97e-02	-3.36e-03	1.69e-02	1.25e-03	1.28e-02
1.33e-02	-7.85e-03	1.14e-02	-1.08e-02	1.89e-02	-9.44e-03	1.62e-02	-1.00e-02	1.23e-02
1.34e-02	9.15e-03	1.14e-02	6.97e-03	1.88e-02	-1.64e-02	1.63e-02	-6.33e-03	1.23e-02
1.54e-02	7.98e-03	1.32e-02	-1.94e-03	2.18e-02	4.70e-03	1.87e-02	1.91e-03	1.42e-02
1.81e-02	-1.86e-02	1.55e-02	9.67e-03	2.55e-02	-1.29e-02	2.20e-02	-3.17e-03	1.66e-02
4.08e-02	2.49e-02	3.49e-02	2.88e-02	5.74e-02	-9.15e-02	5.19e-02	-3.93e-02	3.85e-02
1.64e-02	1.39e-03	1.40e-02	1.94e-02	2.29e-02	5.62e-04	1.98e-02	8.60e-03	1.50e-02
1.40e-02	-4.44e-03	1.20e-02	-8.15e-02	2.00e-02	-3.22e-02	1.71e-02	-5.32e-02	1.30e-02
3.18e-02	-1.75e-02	2.75e-02	-8.07e-02	4.67e-02	4.26e-02	3.81e-02	-7.76e-03	2.95e-02
2.57e-02	1.36e-02	2.19e-02	-5.57e-02	3.70e-02	5.98e-03	3.12e-02	-1.96e-02	2.38e-02
1.38e-02	-1.41e-02	1.18e-02	9.12e-04	1.95e-02	1.10e-02	1.67e-02	6.86e-03	1.27e-02
1.42e-02	-4.10e-02	1.22e-02	-1.39e-02	2.02e-02	-1.66e-02	1.74e-02	-1.55e-02	1.32e-02
1.41e-02	-1.86e-02	1.21e-02	2.27e-02	1.98e-02	9.85e-03	1.71e-02	1.54e-02	1.29e-02
1.41e-02	-8.00e-03	1.21e-02	2.26e-02	1.99e-02	-1.47e-03	1.71e-02	8.65e-03	1.30e-02
1.34e-02	-1.11e-02	1.14e-02	4.09e-03	1.89e-02	-1.92e-02	1.62e-02	-9.30e-03	1.23e-02
1.34e-02	1.50e-02	1.15e-02	2.91e-02	1.90e-02	1.37e-02	1.63e-02	2.01e-02	1.24e-02
1.69e-02	-2.53e-02	1.45e-02	1.57e-02	2.38e-02	-1.59e-02	2.05e-02	-2.61e-03	1.55e-02
1.50e-02	1.79e-02	1.29e-02	8.56e-03	2.12e-02	6.59e-03	1.82e-02	7.53e-03	1.38e-02
1.36e-02	-1.24e-02	1.17e-02	-4.19e-03	1.93e-02	-1.89e-03	1.66e-02	-2.75e-03	1.26e-02
1.38e-02	-4.03e-02	1.18e-02	3.06e-03	1.96e-02	-2.49e-02	1.67e-02	-1.28e-02	1.27e-02
1.53e-02	1.43e-03	1.31e-02	5.73e-02	2.19e-02	5.70e-03	1.86e-02	2.74e-02	1.42e-02
1.34e-02	2.23e-03	1.14e-02	2.34e-02	1.89e-02	-9.54e-03	1.62e-02	4.26e-03	1.23e-02
1.33e-02	1.68e-02	1.14e-02	2.21e-02	1.89e-02	-5.37e-04	1.62e-02	8.95e-03	1.23e-02
1.33e-02	4.63e-03	1.14e-02	-5.68e-03	1.88e-02	1.40e-02	1.61e-02	5.55e-03	1.23e-02
1.33e-02	2.14e-02	1.14e-02	-4.61e-02	1.89e-02	2.47e-02	1.62e-02	-5.52e-03	1.23e-02
1.33e-02	-7.13e-04	1.14e-02	1.02e-02	1.88e-02	-1.50e-02	1.62e-02	-4.30e-03	1.23e-02
1.80e-02	-2.10e-02	1.55e-02	-2.61e-02	2.54e-02	-5.72e-02	2.17e-02	-4.39e-02	1.65e-02
1.37e-02	2.38e-04	1.17e-02	-2.36e-02	1.93e-02	2.85e-03	1.66e-02	-8.32e-03	1.26e-02
1.38e-02	-6.19e-03	1.18e-02	-1.78e-02	1.95e-02	5.15e-03	1.68e-02	-4.54e-03	1.27e-02
1.58e-02	-1.89e-02	1.35e-02	-2.01e-02	2.24e-02	9.65e-03	1.91e-02	-2.65e-03	1.45e-02
1.58e-02	4.11e-02	1.36e-02	-3.63e-02	2.28e-02	-4.21e-02	1.96e-02	-3.93e-02	1.49e-02
1.45e-02	-1.76e-02	1.25e-02	7.76e-04	2.07e-02	2.74e-02	1.78e-02	1.61e-02	1.35e-02
1.33e-02	-1.79e-03	1.14e-02	-2.34e-02	1.88e-02	1.73e-02	1.62e-02	-2.15e-06	1.23e-02
1.34e-02	4.92e-03	1.14e-02	-2.63e-03	1.89e-02	1.79e-02	1.62e-02	9.16e-03	1.23e-02
3.15e-02	1.82e-02	2.68e-02	1.72e-02	4.43e-02	7.35e-02	3.72e-02	4.98e-02	2.85e-02

1.76e-02	-2.31e-02	1.51e-02	-2.14e-02	2.51e-02	3.75e-02	2.18e-02	1.27e-02	1.65e-02
1.36e-02	-1.49e-03	1.16e-02	7.08e-03	1.92e-02	2.09e-02	1.65e-02	1.49e-02	1.25e-02
1.33e-02	1.38e-02	1.14e-02	1.35e-02	1.89e-02	2.14e-02	1.62e-02	1.81e-02	1.23e-02
1.46e-02	-9.14e-03	1.25e-02	-6.81e-03	2.06e-02	-3.37e-03	1.77e-02	-4.83e-03	1.34e-02
1.46e-02	-3.90e-03	1.25e-02	5.48e-02	2.05e-02	4.64e-03	1.78e-02	2.60e-02	1.34e-02
1.65e-02	-2.80e-03	1.42e-02	-4.76e-02	2.37e-02	2.13e-03	2.01e-02	-1.91e-02	1.53e-02
2.29e-02	3.04e-02	1.97e-02	2.46e-02	3.27e-02	3.60e-02	2.79e-02	3.13e-02	2.12e-02
1.59e-02	1.87e-02	1.36e-02	2.10e-02	2.24e-02	4.16e-03	1.94e-02	1.15e-02	1.47e-02
1.34e-02	-7.86e-03	1.15e-02	-1.27e-02	1.90e-02	-1.81e-02	1.63e-02	-1.59e-02	1.23e-02
1.34e-02	9.46e-03	1.15e-02	-8.18e-04	1.89e-02	2.46e-02	1.63e-02	1.37e-02	1.24e-02
1.97e-02	-1.79e-02	1.68e-02	-1.56e-02	2.78e-02	-3.15e-02	2.40e-02	-2.46e-02	1.82e-02
1.88e-02	-1.23e-03	1.62e-02	-4.23e-03	2.68e-02	6.16e-02	2.25e-02	3.41e-02	1.72e-02
1.38e-02	-1.22e-02	1.18e-02	-1.21e-02	1.95e-02	-3.77e-03	1.67e-02	-7.42e-03	1.27e-02
1.37e-02	9.06e-03	1.17e-02	-8.97e-03	1.93e-02	1.49e-02	1.66e-02	4.84e-03	1.26e-02
1.33e-02	1.82e-02	1.14e-02	-1.28e-02	1.89e-02	-9.86e-03	1.62e-02	-1.12e-02	1.23e-02
1.41e-02	1.89e-02	1.20e-02	-4.90e-03	1.99e-02	2.85e-02	1.71e-02	1.44e-02	1.29e-02
1.53e-02	-5.44e-02	1.31e-02	2.69e-02	2.12e-02	-3.21e-02	1.85e-02	-7.04e-03	1.39e-02
1.35e-02	-9.31e-03	1.16e-02	-3.02e-02	1.91e-02	1.84e-02	1.64e-02	-2.38e-03	1.25e-02
1.80e-02	8.65e-03	1.54e-02	-5.96e-03	2.56e-02	2.47e-03	2.19e-02	-9.87e-04	1.67e-02
2.33e-02	1.45e-02	2.00e-02	5.04e-02	3.26e-02	8.00e-03	2.84e-02	2.58e-02	2.14e-02
1.34e-02	-1.24e-03	1.15e-02	9.19e-03	1.90e-02	1.48e-02	1.63e-02	1.23e-02	1.24e-02
1.34e-02	1.63e-02	1.14e-02	2.74e-02	1.88e-02	-2.63e-02	1.63e-02	-3.28e-03	1.23e-02
1.55e-02	8.02e-03	1.33e-02	-2.53e-04	2.20e-02	8.21e-03	1.89e-02	4.73e-03	1.44e-02
1.36e-02	6.19e-03	1.17e-02	2.68e-02	1.93e-02	4.14e-02	1.65e-02	3.53e-02	1.25e-02
3.19e-02	-7.27e-02	2.73e-02	2.62e-03	4.39e-02	-4.00e-02	3.83e-02	-2.19e-02	2.89e-02
3.06e-02	-3.65e-02	2.64e-02	3.31e-02	4.26e-02	-5.03e-02	3.77e-02	-1.47e-02	2.82e-02
1.34e-02	9.34e-03	1.15e-02	-4.91e-03	1.90e-02	8.61e-03	1.63e-02	2.85e-03	1.24e-02
1.38e-02	-1.87e-02	1.18e-02	-1.02e-02	1.95e-02	-7.10e-03	1.67e-02	-8.53e-03	1.27e-02
4.53e-02	-4.05e-02	3.86e-02	5.02e-02	6.12e-02	-1.20e-02	5.42e-02	1.56e-02	4.06e-02
1.92e-02	5.80e-03	1.65e-02	-8.91e-03	2.74e-02	-8.84e-03	2.35e-02	-8.93e-03	1.78e-02
2.30e-02	1.77e-02	1.97e-02	-3.21e-02	3.32e-02	1.15e-02	2.81e-02	-6.72e-03	2.14e-02
1.90e-02	1.19e-02	1.63e-02	9.37e-03	2.69e-02	2.08e-02	2.31e-02	1.59e-02	1.75e-02
1.70e-02	-8.46e-03	1.45e-02	-4.97e-03	2.40e-02	-3.42e-02	2.07e-02	-2.16e-02	1.57e-02
1.58e-02	1.24e-02	1.36e-02	-2.02e-02	2.26e-02	-6.55e-03	1.93e-02	-1.20e-02	1.47e-02
1.34e-02	8.26e-03	1.15e-02	-1.80e-02	1.90e-02	1.94e-02	1.62e-02	3.47e-03	1.23e-02
1.40e-02	-6.92e-03	1.19e-02	2.99e-02	1.96e-02	1.16e-02	1.69e-02	1.94e-02	1.28e-02
1.47e-02	-1.25e-02	1.26e-02	-2.78e-02	2.07e-02	-2.82e-02	1.78e-02	-2.79e-02	1.35e-02
1.33e-02	2.91e-02	1.14e-02	3.88e-02	1.88e-02	1.16e-02	1.61e-02	2.32e-02	1.22e-02
1.33e-02	-2.05e-02	1.14e-02	-2.05e-02	1.88e-02	3.35e-02	1.62e-02	1.06e-02	1.22e-02
1.33e-02	5.33e-03	1.14e-02	1.61e-03	1.89e-02	5.49e-03	1.62e-02	3.74e-03	1.23e-02
1.36e-02	-9.10e-03	1.16e-02	-2.87e-02	1.92e-02	1.77e-02	1.64e-02	-1.93e-03	1.25e-02
1.35e-02	-3.31e-02	1.16e-02	-2.86e-02	1.92e-02	-3.13e-02	1.64e-02	-3.02e-02	1.25e-02
2.16e-02	5.78e-03	1.85e-02	-1.53e-02	3.01e-02	-4.52e-02	2.58e-02	-3.25e-02	1.96e-02
1.41e-02	1.44e-02	1.21e-02	3.08e-03	2.00e-02	-6.14e-05	1.71e-02	1.34e-03	1.30e-02
1.75e-02	6.70e-03	1.50e-02	-1.39e-02	2.47e-02	1.13e-02	2.13e-02	7.94e-04	1.61e-02
1.74e-02	-1.95e-02	1.50e-02	-9.87e-03	2.48e-02	3.91e-02	2.09e-02	1.84e-02	1.60e-02
1.35e-02	-1.91e-02	1.15e-02	-1.50e-02	1.90e-02	-1.36e-02	1.64e-02	-1.44e-02	1.24e-02

1.34e-02	-2.10e-02	1.15e-02	-1.24e-02	1.91e-02	1.30e-02	1.63e-02	2.23e-03	1.24e-02
1.36e-02	-1.72e-04	1.17e-02	7.51e-03	1.93e-02	-1.44e-02	1.66e-02	-4.91e-03	1.26e-02
1.32e-02	-1.07e-03	1.13e-02	9.99e-03	1.87e-02	-1.82e-02	1.61e-02	-6.11e-03	1.22e-02
1.44e-02	-5.83e-04	1.23e-02	-2.80e-02	2.05e-02	-4.69e-03	1.75e-02	-1.47e-02	1.33e-02
1.34e-02	2.97e-03	1.15e-02	5.21e-03	1.91e-02	-4.00e-02	1.63e-02	-2.10e-02	1.24e-02
1.34e-02	-8.20e-03	1.15e-02	-2.33e-02	1.90e-02	2.62e-02	1.63e-02	5.12e-03	1.24e-02
1.36e-02	2.01e-02	1.16e-02	3.38e-03	1.92e-02	-9.77e-03	1.66e-02	-3.87e-03	1.25e-02
1.61e-02	1.65e-02	1.39e-02	4.61e-02	2.28e-02	2.24e-02	1.96e-02	3.27e-02	1.49e-02
2.54e-02	-1.32e-02	2.16e-02	3.94e-02	3.50e-02	2.84e-02	3.02e-02	3.27e-02	2.29e-02
2.11e-02	1.09e-02	1.80e-02	1.19e-03	2.96e-02	5.10e-02	2.52e-02	2.97e-02	1.92e-02
2.17e-02	-3.17e-02	1.85e-02	4.05e-03	3.03e-02	-1.04e-02	2.61e-02	-4.24e-03	1.97e-02
1.35e-02	-4.25e-03	1.16e-02	-4.26e-02	1.91e-02	1.76e-02	1.64e-02	-7.99e-03	1.24e-02
1.34e-02	4.32e-02	1.14e-02	4.00e-03	1.89e-02	2.33e-02	1.63e-02	1.52e-02	1.23e-02
1.36e-02	-1.10e-02	1.17e-02	-5.81e-02	1.93e-02	-3.32e-02	1.66e-02	-4.39e-02	1.26e-02
1.95e-02	2.19e-02	1.68e-02	4.54e-03	2.79e-02	3.72e-02	2.37e-02	2.34e-02	1.80e-02
1.80e-02	2.33e-03	1.54e-02	-3.39e-03	2.54e-02	-1.18e-02	2.19e-02	-8.05e-03	1.66e-02
1.41e-02	-3.93e-03	1.21e-02	-2.83e-02	2.00e-02	4.93e-03	1.71e-02	-9.28e-03	1.30e-02
1.86e-02	-1.82e-02	1.60e-02	-1.51e-02	2.64e-02	-6.33e-02	2.30e-02	-4.29e-02	1.73e-02
1.93e-02	1.69e-02	1.64e-02	-2.68e-02	2.75e-02	-1.16e-02	2.35e-02	-1.79e-02	1.79e-02
1.61e-02	-9.11e-03	1.38e-02	-2.45e-02	2.29e-02	3.28e-02	1.93e-02	8.66e-03	1.48e-02
1.82e-02	-2.44e-02	1.56e-02	-4.07e-02	2.59e-02	-4.34e-02	2.23e-02	-4.23e-02	1.69e-02
2.09e-02	-2.48e-02	1.80e-02	5.04e-02	2.89e-02	-1.54e-02	2.54e-02	1.30e-02	1.91e-02
1.64e-02	-2.65e-03	1.40e-02	2.23e-03	2.32e-02	1.74e-02	2.00e-02	1.10e-02	1.51e-02
1.33e-02	4.97e-03	1.14e-02	2.46e-02	1.88e-02	2.44e-02	1.61e-02	2.46e-02	1.22e-02
1.34e-02	1.81e-02	1.15e-02	-4.54e-04	1.90e-02	2.18e-02	1.63e-02	1.25e-02	1.24e-02
1.33e-02	4.68e-02	1.14e-02	3.97e-02	1.89e-02	1.64e-02	1.62e-02	2.63e-02	1.23e-02
2.52e-02	3.08e-02	2.16e-02	-2.65e-02	3.64e-02	3.06e-02	3.07e-02	6.66e-03	2.34e-02
1.44e-02	6.38e-03	1.24e-02	1.77e-02	2.04e-02	6.66e-03	1.76e-02	1.14e-02	1.33e-02
1.59e-02	-6.02e-03	1.37e-02	-1.47e-02	2.26e-02	-5.12e-03	1.94e-02	-9.04e-03	1.47e-02
3.24e-02	-2.45e-02	2.81e-02	-6.59e-02	4.76e-02	-1.66e-02	3.96e-02	-3.71e-02	3.04e-02
1.37e-02	-1.91e-02	1.17e-02	-4.65e-02	1.95e-02	-2.73e-02	1.67e-02	-3.54e-02	1.27e-02
1.39e-02	-2.47e-02	1.19e-02	-1.85e-02	1.97e-02	1.69e-03	1.69e-02	-6.72e-03	1.28e-02
1.71e-02	-2.81e-02	1.46e-02	1.36e-02	2.38e-02	1.68e-02	2.04e-02	1.55e-02	1.55e-02
1.42e-02	5.50e-03	1.21e-02	-2.22e-02	2.00e-02	-5.13e-03	1.72e-02	-1.24e-02	1.30e-02
1.60e-02	4.52e-03	1.37e-02	2.51e-02	2.27e-02	2.23e-02	1.94e-02	2.35e-02	1.47e-02
1.84e-02	-3.67e-02	1.59e-02	-2.14e-02	2.62e-02	-4.32e-02	2.26e-02	-3.37e-02	1.71e-02
1.33e-02	7.26e-03	1.14e-02	1.01e-02	1.88e-02	-1.08e-02	1.62e-02	-1.79e-03	1.23e-02
1.98e-02	-2.51e-03	1.71e-02	2.47e-02	2.78e-02	1.77e-02	2.41e-02	2.08e-02	1.82e-02
1.33e-02	4.25e-03	1.14e-02	7.69e-03	1.88e-02	1.28e-02	1.61e-02	1.05e-02	1.22e-02
1.39e-02	-3.26e-02	1.20e-02	-2.96e-02	1.98e-02	-1.89e-02	1.69e-02	-2.32e-02	1.29e-02
1.53e-02	1.56e-02	1.31e-02	-1.25e-02	2.17e-02	2.58e-02	1.86e-02	9.60e-03	1.41e-02
1.76e-02	-1.72e-02	1.51e-02	1.08e-02	2.46e-02	-2.83e-02	2.15e-02	-1.16e-02	1.62e-02
1.34e-02	6.08e-04	1.14e-02	-2.24e-02	1.89e-02	-4.81e-02	1.63e-02	-3.71e-02	1.23e-02
1.61e-02	1.43e-02	1.38e-02	3.38e-02	2.29e-02	-8.94e-04	1.95e-02	1.38e-02	1.48e-02
1.44e-02	-3.61e-03	1.24e-02	-2.11e-02	2.05e-02	-3.06e-03	1.75e-02	-1.05e-02	1.33e-02
1.34e-02	-1.46e-02	1.15e-02	1.01e-02	1.90e-02	-4.87e-03	1.63e-02	1.44e-03	1.24e-02
1.35e-02	5.88e-03	1.16e-02	-3.01e-03	1.91e-02	-9.86e-03	1.64e-02	-6.88e-03	1.25e-02

1.48e-02	-5.84e-03	1.27e-02	-2.63e-02	2.09e-02	5.03e-03	1.80e-02	-8.05e-03	1.36e-02
1.38e-02	4.23e-02	1.18e-02	1.49e-02	1.96e-02	2.02e-02	1.67e-02	1.78e-02	1.27e-02
1.46e-02	2.30e-02	1.25e-02	-2.46e-02	2.04e-02	-5.09e-03	1.77e-02	-1.36e-02	1.33e-02
1.42e-02	-3.38e-03	1.22e-02	-1.43e-02	2.01e-02	-1.56e-02	1.73e-02	-1.49e-02	1.31e-02
1.41e-02	2.60e-02	1.21e-02	2.12e-04	2.00e-02	2.26e-02	1.72e-02	1.32e-02	1.30e-02
1.50e-02	-1.23e-02	1.29e-02	-3.96e-04	2.11e-02	-2.91e-02	1.83e-02	-1.69e-02	1.38e-02
1.52e-02	-2.50e-02	1.30e-02	-9.90e-04	2.15e-02	1.01e-02	1.86e-02	5.41e-03	1.41e-02
1.35e-02	3.35e-02	1.16e-02	-3.37e-02	1.90e-02	-6.93e-03	1.64e-02	-1.85e-02	1.24e-02
1.53e-02	3.50e-02	1.31e-02	2.33e-03	2.18e-02	3.58e-02	1.85e-02	2.15e-02	1.41e-02
1.48e-02	-5.51e-04	1.27e-02	1.92e-02	2.10e-02	-2.40e-02	1.79e-02	-5.39e-03	1.36e-02
1.36e-02	-2.23e-02	1.16e-02	2.42e-02	1.92e-02	-5.09e-04	1.65e-02	1.01e-02	1.25e-02
1.47e-02	-2.19e-02	1.26e-02	-2.38e-04	2.07e-02	-1.38e-02	1.78e-02	-7.95e-03	1.35e-02
1.34e-02	-6.60e-03	1.15e-02	-2.59e-02	1.89e-02	2.05e-02	1.63e-02	7.69e-04	1.23e-02
1.33e-02	-8.26e-03	1.14e-02	5.55e-03	1.89e-02	3.91e-03	1.62e-02	4.69e-03	1.23e-02
1.50e-02	-3.86e-03	1.28e-02	3.25e-02	2.10e-02	1.37e-02	1.82e-02	2.18e-02	1.38e-02
1.43e-02	1.05e-02	1.23e-02	4.13e-02	2.04e-02	-9.43e-04	1.74e-02	1.70e-02	1.32e-02
1.42e-02	-5.18e-03	1.22e-02	-1.72e-02	2.01e-02	-3.96e-03	1.73e-02	-9.72e-03	1.31e-02
1.37e-02	1.02e-02	1.18e-02	6.26e-03	1.94e-02	1.07e-02	1.67e-02	8.63e-03	1.26e-02
1.33e-02	1.15e-02	1.14e-02	5.75e-03	1.89e-02	-1.53e-02	1.62e-02	-6.45e-03	1.23e-02
1.32e-02	-1.06e-02	1.14e-02	-2.58e-02	1.87e-02	1.34e-02	1.61e-02	-2.98e-03	1.22e-02
1.45e-02	-2.48e-02	1.24e-02	-1.72e-02	2.05e-02	-2.80e-02	1.75e-02	-2.32e-02	1.33e-02
2.48e-02	-1.25e-02	2.14e-02	4.24e-02	3.45e-02	-3.79e-02	3.06e-02	-3.24e-03	2.29e-02
1.49e-02	-1.04e-02	1.28e-02	-2.68e-02	2.11e-02	-4.81e-03	1.81e-02	-1.42e-02	1.37e-02
1.34e-02	4.06e-03	1.15e-02	-7.61e-03	1.90e-02	-1.02e-02	1.62e-02	-9.04e-03	1.23e-02
1.36e-02	5.48e-03	1.17e-02	2.99e-03	1.93e-02	2.43e-03	1.65e-02	2.79e-03	1.25e-02
1.42e-02	-2.49e-03	1.21e-02	-1.03e-03	2.00e-02	1.07e-02	1.73e-02	5.85e-03	1.31e-02
6.62e-02	2.30e-02	5.69e-02	-1.75e-02	9.78e-02	1.96e-02	8.03e-02	5.16e-03	6.20e-02
1.33e-02	9.67e-03	1.14e-02	-5.09e-04	1.88e-02	4.06e-03	1.61e-02	2.07e-03	1.22e-02
1.49e-02	-3.46e-03	1.27e-02	4.55e-02	2.08e-02	3.35e-02	1.79e-02	3.85e-02	1.36e-02
1.35e-02	5.05e-03	1.16e-02	9.94e-03	1.91e-02	-4.36e-03	1.64e-02	1.70e-03	1.24e-02
1.60e-02	-2.54e-02	1.36e-02	-1.49e-02	2.25e-02	1.08e-03	1.94e-02	-5.61e-03	1.47e-02
1.34e-02	-2.58e-03	1.15e-02	2.26e-02	1.91e-02	-2.99e-02	1.63e-02	-7.73e-03	1.24e-02

STR femaleI	STR femalI	STR male	H STR male	STR overallI	STR overaI	female Be	CH female S	CH male Bet
-1.24e-03	2.14e-02	-6.43e-03	1.75e-02	-4.45e-03	1.36e-02	8.61e-02	5.11e-02	-5.18e-02
6.33e-02	2.10e-02	-3.94e-03	1.71e-02	2.30e-02	1.33e-02	1.92e-02	5.05e-02	2.36e-02
-9.29e-03	2.14e-02	1.86e-02	1.76e-02	7.55e-03	1.36e-02	1.09e-01	5.23e-02	-2.98e-03
1.52e-02	2.14e-02	-1.63e-02	1.76e-02	-3.82e-03	1.36e-02	-6.76e-02	5.21e-02	3.79e-03
1.16e-02	2.08e-02	1.01e-02	1.71e-02	1.05e-02	1.32e-02	1.24e-02	5.02e-02	-3.00e-03
1.42e-02	2.09e-02	9.56e-03	1.71e-02	1.14e-02	1.32e-02	-1.65e-03	5.03e-02	2.33e-02
1.36e-02	2.22e-02	3.12e-02	1.83e-02	2.40e-02	1.41e-02	9.40e-03	5.35e-02	4.14e-02
2.64e-03	2.08e-02	-1.51e-03	1.71e-02	2.02e-04	1.32e-02	7.27e-02	5.03e-02	-3.92e-02
-2.54e-02	2.24e-02	1.92e-03	1.84e-02	-8.98e-03	1.42e-02	1.22e-01	5.27e-02	-5.57e-02
-1.11e-02	2.14e-02	7.68e-03	1.76e-02	1.96e-04	1.36e-02	7.11e-02	5.22e-02	2.70e-02
4.54e-03	2.23e-02	3.42e-02	1.81e-02	2.23e-02	1.41e-02	9.08e-02	5.30e-02	5.53e-02
-1.22e-02	2.13e-02	-3.02e-02	1.75e-02	-2.29e-02	1.35e-02	-1.29e-02	5.13e-02	-6.07e-02
-2.23e-02	2.58e-02	-3.09e-03	2.10e-02	-1.08e-02	1.63e-02	-5.25e-02	6.27e-02	-7.25e-03
4.26e-03	2.11e-02	-3.43e-02	1.73e-02	-1.87e-02	1.34e-02	3.35e-02	5.11e-02	-3.47e-02
1.73e-02	4.42e-02	-8.82e-02	3.79e-02	-4.47e-02	2.88e-02	-1.28e-01	1.13e-01	-3.14e-02
-6.71e-03	2.17e-02	1.92e-03	1.78e-02	-1.34e-03	1.38e-02	6.96e-03	5.24e-02	2.50e-02
2.29e-02	2.20e-02	-1.27e-02	1.81e-02	1.69e-03	1.40e-02	9.43e-03	5.31e-02	-1.57e-02
-9.35e-04	2.70e-02	-2.52e-02	2.22e-02	-1.54e-02	1.72e-02	1.47e-02	6.48e-02	1.03e-02
4.53e-02	2.12e-02	1.54e-03	1.74e-02	1.90e-02	1.35e-02	3.24e-02	5.12e-02	6.43e-02
-2.29e-02	2.46e-02	-1.37e-02	2.02e-02	-1.76e-02	1.56e-02	1.10e-01	5.74e-02	4.38e-02
-2.44e-02	2.70e-02	2.44e-02	2.18e-02	5.18e-03	1.69e-02	-1.07e-01	6.68e-02	-3.03e-02
-1.69e-02	2.12e-02	-7.35e-03	1.74e-02	-1.12e-02	1.35e-02	-5.40e-02	5.10e-02	7.85e-02
2.40e-02	2.26e-02	3.93e-03	1.87e-02	1.20e-02	1.44e-02	4.36e-02	5.43e-02	-2.00e-02
-1.38e-02	2.13e-02	8.75e-05	1.75e-02	-5.45e-03	1.35e-02	-8.31e-02	5.12e-02	3.10e-02
4.08e-02	2.10e-02	1.66e-02	1.73e-02	2.66e-02	1.34e-02	1.97e-02	5.08e-02	4.16e-02
2.95e-02	2.64e-02	-9.84e-03	2.18e-02	5.76e-03	1.68e-02	4.80e-03	6.41e-02	-4.65e-02
2.93e-03	2.82e-02	-3.83e-02	2.34e-02	-2.13e-02	1.80e-02	-8.49e-03	6.82e-02	2.20e-02
-1.13e-02	2.09e-02	-2.21e-02	1.72e-02	-1.78e-02	1.33e-02	9.82e-02	5.07e-02	1.62e-02
-6.62e-02	2.19e-02	-1.19e-02	1.78e-02	-3.35e-02	1.38e-02	1.24e-01	5.16e-02	-1.74e-02
3.33e-02	2.25e-02	-7.25e-03	1.86e-02	9.13e-03	1.43e-02	-5.72e-02	5.52e-02	-4.41e-02
-2.07e-02	3.46e-02	-8.54e-02	2.89e-02	-5.93e-02	2.22e-02	-2.76e-02	8.38e-02	2.74e-02
-5.45e-03	2.70e-02	4.47e-02	2.18e-02	2.47e-02	1.70e-02	-7.91e-02	6.65e-02	1.29e-01
-2.87e-02	2.14e-02	-2.90e-02	1.75e-02	-2.89e-02	1.35e-02	1.73e-02	5.17e-02	-8.16e-02
6.55e-03	2.81e-02	3.61e-02	2.29e-02	2.42e-02	1.78e-02	6.47e-02	6.65e-02	-8.22e-02
2.57e-02	2.12e-02	-2.10e-02	1.74e-02	-2.13e-03	1.34e-02	-2.81e-02	5.13e-02	-3.46e-02
-2.30e-02	2.09e-02	-1.39e-02	1.71e-02	-1.76e-02	1.32e-02	-7.42e-02	5.04e-02	-6.25e-03
4.79e-02	3.25e-02	3.45e-02	2.68e-02	3.99e-02	2.07e-02	-3.22e-02	8.06e-02	1.29e-02
1.90e-02	4.32e-02	-7.31e-03	3.61e-02	3.61e-03	2.77e-02	-2.29e-02	1.06e-01	1.40e-01
-7.04e-02	4.07e-02	2.12e-02	3.19e-02	-1.43e-02	2.51e-02	1.88e-01	8.86e-02	3.89e-02
-3.93e-03	2.19e-02	-5.79e-03	1.79e-02	-4.79e-03	1.39e-02	1.16e-01	5.19e-02	-2.00e-02
6.57e-02	2.72e-02	-1.52e-02	2.28e-02	1.79e-02	1.75e-02	-1.33e-02	6.71e-02	7.43e-03
4.58e-02	2.09e-02	7.40e-03	1.71e-02	2.29e-02	1.32e-02	7.37e-03	5.03e-02	5.35e-03
-5.26e-02	2.49e-02	-4.45e-02	2.04e-02	-4.77e-02	1.58e-02	5.26e-02	5.85e-02	5.52e-03
2.35e-02	2.11e-02	-1.42e-02	1.73e-02	7.55e-04	1.34e-02	2.19e-02	5.09e-02	-8.45e-02
1.45e-02	3.25e-02	2.74e-02	2.67e-02	2.20e-02	2.06e-02	1.58e-01	7.44e-02	-5.08e-02
-1.86e-02	2.23e-02	-1.83e-02	1.83e-02	-1.85e-02	1.41e-02	2.95e-02	5.33e-02	-8.62e-02

-3.55e-02	2.09e-02	-1.81e-02	1.71e-02	-2.49e-02	1.33e-02	7.89e-02	5.03e-02	5.91e-02
-4.07e-02	3.44e-02	1.27e-02	2.77e-02	-8.70e-03	2.16e-02	-2.77e-02	8.28e-02	-1.11e-01
5.87e-03	2.73e-02	2.61e-02	2.24e-02	1.81e-02	1.73e-02	1.52e-02	6.60e-02	-3.36e-02
-6.05e-02	2.44e-02	-3.83e-02	1.98e-02	-4.72e-02	1.54e-02	-2.25e-02	5.83e-02	-4.68e-02
-2.46e-03	2.07e-02	-1.33e-02	1.70e-02	-8.95e-03	1.32e-02	1.36e-02	4.99e-02	-2.60e-02
3.22e-02	2.39e-02	-6.37e-04	1.98e-02	1.28e-02	1.53e-02	1.54e-02	5.79e-02	-1.01e-02
5.53e-02	3.22e-02	1.14e-02	2.68e-02	2.93e-02	2.06e-02	3.72e-02	7.80e-02	4.96e-02
-8.65e-03	3.10e-02	6.30e-03	2.53e-02	4.39e-04	1.96e-02	-1.23e-01	7.82e-02	5.13e-02
-4.64e-03	2.38e-02	8.41e-03	1.95e-02	3.22e-03	1.51e-02	-6.02e-02	5.82e-02	-4.13e-02
-9.02e-02	2.92e-02	-5.22e-03	2.32e-02	-3.90e-02	1.82e-02	-7.47e-02	7.01e-02	6.01e-02
-9.76e-02	4.80e-02	-5.04e-02	3.87e-02	-6.94e-02	3.01e-02	-5.97e-03	1.12e-01	-8.70e-02
-2.04e-02	2.18e-02	-1.57e-02	1.79e-02	-1.77e-02	1.38e-02	-3.21e-02	5.24e-02	1.06e-02
-1.41e-02	2.23e-02	-8.27e-03	1.83e-02	-1.05e-02	1.41e-02	-9.85e-02	5.31e-02	5.77e-02
-3.07e-02	2.77e-02	-2.18e-03	2.25e-02	-1.38e-02	1.74e-02	3.95e-03	6.61e-02	7.72e-02
-2.60e-02	3.15e-02	-2.05e-02	2.58e-02	-2.28e-02	2.00e-02	7.55e-03	7.51e-02	1.64e-02
2.58e-04	2.30e-02	-2.88e-03	1.88e-02	-1.58e-03	1.46e-02	-2.27e-02	5.56e-02	1.18e-01
1.34e-02	2.09e-02	-2.04e-03	1.72e-02	4.21e-03	1.33e-02	-2.70e-02	5.05e-02	1.50e-02
2.67e-02	2.35e-02	1.94e-03	1.94e-02	1.21e-02	1.50e-02	4.21e-02	5.66e-02	4.18e-02
3.55e-02	2.18e-02	2.99e-02	1.79e-02	3.21e-02	1.38e-02	-2.62e-02	5.31e-02	-1.38e-03
-1.39e-02	2.09e-02	3.71e-02	1.72e-02	1.67e-02	1.33e-02	5.79e-02	5.04e-02	-1.99e-02
-1.74e-02	2.65e-02	1.64e-03	2.18e-02	-6.04e-03	1.69e-02	-4.45e-02	6.45e-02	-1.22e-01
2.32e-02	2.27e-02	2.17e-02	1.86e-02	2.24e-02	1.44e-02	-7.25e-02	5.38e-02	5.89e-02
-2.01e-02	3.24e-02	3.10e-02	2.60e-02	1.11e-02	2.03e-02	3.94e-03	7.76e-02	9.69e-02
5.76e-02	2.19e-02	-1.84e-02	1.82e-02	1.21e-02	1.40e-02	-7.68e-02	5.40e-02	-1.58e-02
-6.89e-03	2.20e-02	-1.15e-02	1.80e-02	-9.84e-03	1.39e-02	-1.53e-02	5.29e-02	3.46e-02
2.57e-02	2.38e-02	-3.92e-03	1.94e-02	7.83e-03	1.50e-02	-7.85e-02	5.60e-02	7.08e-03
-3.76e-02	4.69e-02	-1.74e-02	3.83e-02	-2.57e-02	2.97e-02	-4.60e-02	1.14e-01	3.11e-02
-2.05e-02	2.96e-02	6.71e-03	2.41e-02	-4.24e-03	1.87e-02	-4.02e-02	7.18e-02	7.22e-02
3.74e-03	2.09e-02	-1.05e-02	1.71e-02	-4.82e-03	1.32e-02	2.05e-02	5.03e-02	2.73e-02
8.57e-03	2.21e-02	-1.28e-03	1.81e-02	2.64e-03	1.40e-02	-8.56e-02	5.26e-02	-6.78e-03
-1.03e-02	2.33e-02	-3.65e-02	1.92e-02	-2.58e-02	1.48e-02	2.24e-02	5.59e-02	4.93e-02
6.58e-03	2.09e-02	1.15e-02	1.72e-02	9.49e-03	1.33e-02	-2.53e-02	5.04e-02	8.35e-02
1.23e-02	2.50e-02	1.01e-02	2.05e-02	1.09e-02	1.59e-02	9.78e-02	5.90e-02	-1.61e-02
5.49e-02	2.23e-02	9.09e-03	1.81e-02	2.73e-02	1.40e-02	-2.23e-02	5.31e-02	-1.45e-02
1.70e-02	2.22e-02	-4.67e-03	1.82e-02	4.18e-03	1.41e-02	5.13e-02	5.39e-02	3.77e-02
4.51e-03	2.08e-02	2.10e-02	1.70e-02	1.45e-02	1.32e-02	-1.69e-02	5.02e-02	3.11e-02
-3.06e-03	2.15e-02	-1.85e-02	1.76e-02	-1.25e-02	1.36e-02	4.92e-03	5.17e-02	3.49e-02
-8.23e-03	2.21e-02	1.03e-02	1.82e-02	2.96e-03	1.40e-02	5.24e-02	5.29e-02	6.43e-02
-1.85e-02	2.22e-02	7.26e-03	1.81e-02	-3.13e-03	1.40e-02	-6.72e-02	5.39e-02	-7.81e-02
-6.61e-03	2.09e-02	-6.02e-03	1.72e-02	-6.31e-03	1.33e-02	5.08e-03	5.04e-02	-1.32e-03
1.32e-02	2.50e-02	6.20e-03	2.04e-02	9.19e-03	1.58e-02	-4.48e-02	5.93e-02	1.13e-01
1.09e-02	2.21e-02	-2.07e-02	1.81e-02	-8.08e-03	1.40e-02	2.34e-02	5.34e-02	-2.04e-02
2.91e-02	2.37e-02	-3.22e-02	1.97e-02	-7.38e-03	1.52e-02	-3.77e-02	5.80e-02	-1.30e-02
1.38e-02	2.15e-02	-4.44e-02	1.77e-02	-2.10e-02	1.37e-02	-1.76e-02	5.21e-02	-2.03e-03
2.09e-04	2.42e-02	1.88e-02	1.98e-02	1.13e-02	1.53e-02	9.37e-02	5.70e-02	9.19e-03
2.72e-03	2.09e-02	1.05e-02	1.71e-02	7.47e-03	1.32e-02	-5.80e-02	5.04e-02	3.80e-02
4.27e-02	2.37e-02	9.68e-03	1.95e-02	2.31e-02	1.51e-02	8.07e-02	5.66e-02	4.70e-02

-1.26e-02	2.15e-02	-2.87e-02	1.76e-02	-2.22e-02	1.36e-02	5.39e-02	5.14e-02	-2.52e-02
5.27e-02	2.09e-02	1.64e-02	1.72e-02	3.09e-02	1.33e-02	-2.23e-02	5.06e-02	5.98e-03
4.17e-02	2.10e-02	1.16e-02	1.72e-02	2.39e-02	1.33e-02	7.84e-02	5.09e-02	2.67e-02
2.07e-02	2.10e-02	2.13e-02	1.73e-02	2.11e-02	1.33e-02	2.45e-02	5.06e-02	-2.07e-02
2.79e-02	3.40e-02	3.05e-03	2.81e-02	1.32e-02	2.17e-02	-8.75e-03	8.32e-02	-5.78e-02
-1.95e-02	2.10e-02	-1.41e-02	1.72e-02	-1.63e-02	1.33e-02	-1.62e-04	5.07e-02	-1.40e-01
5.26e-02	2.95e-02	2.92e-02	2.45e-02	3.89e-02	1.89e-02	-1.39e-04	7.23e-02	-3.92e-02
-1.14e-02	2.09e-02	-1.97e-02	1.71e-02	-1.65e-02	1.32e-02	1.31e-02	5.04e-02	1.13e-01
-2.79e-02	2.15e-02	1.91e-02	1.76e-02	3.07e-04	1.36e-02	6.92e-02	5.14e-02	3.40e-03
2.93e-02	7.61e-02	2.25e-02	6.21e-02	2.49e-02	4.81e-02	2.22e-01	1.67e-01	5.62e-02
1.86e-03	2.20e-02	1.27e-02	1.80e-02	8.08e-03	1.39e-02	-9.04e-02	5.38e-02	-9.64e-02
-4.05e-03	2.16e-02	2.76e-02	1.77e-02	1.52e-02	1.37e-02	-7.60e-02	5.27e-02	-1.56e-02
1.19e-02	2.96e-02	-1.56e-02	2.40e-02	-4.64e-03	1.87e-02	-4.31e-02	7.02e-02	4.29e-02
-2.21e-02	2.10e-02	-8.38e-04	1.72e-02	-9.45e-03	1.33e-02	5.92e-02	5.04e-02	-7.88e-02
4.15e-02	2.58e-02	4.35e-03	2.14e-02	1.95e-02	1.65e-02	3.50e-02	6.24e-02	-1.90e-02
2.43e-02	2.38e-02	-4.23e-02	1.93e-02	-1.57e-02	1.50e-02	1.19e-01	5.88e-02	4.87e-02
2.47e-02	2.66e-02	7.43e-04	2.17e-02	1.05e-02	1.68e-02	1.06e-01	6.59e-02	-9.25e-02
-1.35e-02	2.69e-02	-1.74e-02	2.22e-02	-1.60e-02	1.71e-02	-2.36e-02	6.51e-02	7.57e-02
-1.73e-02	3.87e-02	-2.56e-02	3.18e-02	-2.23e-02	2.46e-02	-6.31e-02	9.50e-02	-1.28e-02
8.59e-04	3.80e-02	5.07e-03	3.09e-02	3.62e-03	2.40e-02	1.35e-02	9.10e-02	1.23e-02
1.23e-02	2.10e-02	1.46e-02	1.73e-02	1.38e-02	1.33e-02	-9.65e-02	5.11e-02	3.68e-02
3.08e-03	2.08e-02	1.28e-02	1.71e-02	8.76e-03	1.32e-02	6.85e-02	5.03e-02	-6.21e-02
2.15e-02	2.14e-02	-2.18e-02	1.77e-02	-4.18e-03	1.36e-02	4.65e-02	5.15e-02	-5.25e-03
3.89e-03	2.47e-02	-3.35e-02	2.03e-02	-1.84e-02	1.57e-02	-8.28e-03	5.97e-02	-1.16e-01
3.13e-03	2.11e-02	-2.93e-03	1.73e-02	-3.67e-04	1.33e-02	-2.80e-02	5.09e-02	7.59e-02
-1.49e-02	2.48e-02	-2.10e-02	2.03e-02	-1.84e-02	1.57e-02	-1.17e-02	5.99e-02	3.91e-02
-1.04e-02	2.14e-02	-2.19e-02	1.75e-02	-1.73e-02	1.36e-02	-6.83e-02	5.19e-02	-1.48e-01
2.96e-02	2.11e-02	2.99e-03	1.72e-02	1.36e-02	1.33e-02	-2.34e-02	5.07e-02	-1.04e-01
1.06e-02	2.12e-02	2.95e-02	1.73e-02	2.20e-02	1.34e-02	-1.28e-02	5.09e-02	8.45e-02
2.93e-02	2.11e-02	-4.50e-03	1.73e-02	8.77e-03	1.34e-02	5.02e-02	5.09e-02	1.69e-02
-1.50e-02	2.87e-02	-2.05e-02	2.36e-02	-1.85e-02	1.82e-02	-1.40e-02	6.92e-02	1.50e-01
9.81e-03	2.08e-02	7.67e-03	1.71e-02	8.33e-03	1.32e-02	-1.85e-02	5.01e-02	1.47e-02
3.97e-03	2.09e-02	-2.41e-02	1.71e-02	-1.29e-02	1.32e-02	3.16e-02	5.05e-02	1.79e-02
-3.89e-02	2.12e-02	-1.48e-02	1.74e-02	-2.44e-02	1.35e-02	-6.44e-02	5.10e-02	4.29e-04
-3.98e-02	2.23e-02	-2.33e-02	1.83e-02	-2.98e-02	1.41e-02	6.74e-02	5.29e-02	-4.83e-02
7.86e-02	2.99e-02	1.49e-02	2.51e-02	4.09e-02	1.92e-02	2.22e-02	7.35e-02	-9.89e-02
3.39e-02	2.28e-02	9.41e-03	1.87e-02	1.92e-02	1.44e-02	1.29e-02	5.47e-02	-3.83e-02
-3.02e-02	2.34e-02	-1.74e-02	1.92e-02	-2.23e-02	1.48e-02	7.35e-02	5.52e-02	5.47e-02
-1.70e-02	2.13e-02	-2.97e-02	1.74e-02	-2.47e-02	1.35e-02	4.94e-02	5.10e-02	-9.14e-03
5.44e-02	3.39e-02	-3.70e-04	2.84e-02	2.20e-02	2.18e-02	1.05e-01	7.99e-02	1.91e-03
1.25e-02	2.18e-02	-3.33e-02	1.78e-02	-1.50e-02	1.38e-02	3.86e-02	5.28e-02	-8.32e-02
-1.01e-02	2.70e-02	2.32e-02	2.20e-02	1.00e-02	1.70e-02	-5.34e-02	6.60e-02	-7.69e-02
2.73e-02	2.70e-02	-1.89e-02	2.24e-02	-2.37e-04	1.72e-02	1.58e-02	6.54e-02	6.24e-02
1.46e-02	2.19e-02	-2.27e-02	1.79e-02	-7.77e-03	1.39e-02	-5.41e-02	5.24e-02	4.36e-02
-3.54e-02	3.00e-02	-3.38e-02	2.46e-02	-3.45e-02	1.90e-02	1.20e-02	7.13e-02	3.55e-02
-4.01e-02	2.45e-02	-2.83e-02	2.01e-02	-3.31e-02	1.55e-02	8.79e-02	5.72e-02	9.83e-03
2.91e-02	2.19e-02	-5.81e-03	1.79e-02	7.76e-03	1.38e-02	-2.45e-02	5.23e-02	8.45e-03

-2.17e-02	2.42e-02	-6.60e-04	1.98e-02	-8.94e-03	1.53e-02	4.78e-02	5.74e-02	4.59e-02
-5.52e-03	2.13e-02	-4.11e-02	1.75e-02	-2.68e-02	1.35e-02	6.63e-02	5.19e-02	-4.58e-02
4.60e-02	2.41e-02	1.65e-02	1.96e-02	2.83e-02	1.52e-02	-1.17e-02	5.74e-02	5.60e-02
-7.17e-03	2.12e-02	8.60e-03	1.74e-02	2.19e-03	1.34e-02	2.43e-02	5.12e-02	6.47e-03
-3.02e-02	2.18e-02	2.50e-03	1.80e-02	-1.08e-02	1.39e-02	7.11e-02	5.34e-02	5.54e-02
-5.99e-02	2.80e-02	3.77e-03	2.25e-02	-2.15e-02	1.75e-02	1.19e-02	6.60e-02	2.03e-02
1.63e-02	2.08e-02	1.92e-02	1.71e-02	1.80e-02	1.32e-02	-2.80e-02	5.03e-02	1.90e-02
-1.25e-02	2.13e-02	1.55e-02	1.74e-02	4.36e-03	1.35e-02	3.36e-02	5.12e-02	-2.50e-03
-3.08e-02	2.23e-02	-1.16e-02	1.82e-02	-1.93e-02	1.41e-02	-5.55e-02	5.41e-02	-5.34e-02
2.54e-02	2.09e-02	-3.27e-03	1.71e-02	8.11e-03	1.32e-02	5.68e-02	5.03e-02	-2.31e-02
-7.93e-03	2.70e-02	-3.12e-02	2.24e-02	-2.18e-02	1.72e-02	-5.90e-02	6.63e-02	-3.28e-02
1.96e-02	2.40e-02	2.49e-03	1.97e-02	9.51e-03	1.52e-02	4.03e-02	5.75e-02	-9.13e-02
-2.30e-03	2.19e-02	-2.56e-03	1.79e-02	-2.21e-03	1.39e-02	-4.00e-02	5.31e-02	-2.38e-02
-1.31e-02	2.09e-02	-2.16e-02	1.71e-02	-1.82e-02	1.33e-02	6.07e-02	5.04e-02	4.55e-02
1.10e-02	2.09e-02	-1.51e-02	1.72e-02	-4.45e-03	1.33e-02	-1.22e-03	5.04e-02	-2.26e-02
-6.94e-03	2.42e-02	-7.17e-03	1.99e-02	-7.07e-03	1.53e-02	-1.19e-02	5.83e-02	5.05e-02
1.49e-03	2.83e-02	-1.15e-02	2.32e-02	-6.20e-03	1.80e-02	-6.76e-05	6.83e-02	4.98e-02
1.21e-01	6.11e-02	-9.14e-02	5.50e-02	-1.31e-03	4.09e-02	-2.13e-01	1.71e-01	1.30e-02
2.65e-02	2.53e-02	-5.07e-03	2.10e-02	7.75e-03	1.62e-02	3.26e-03	6.15e-02	-6.14e-02
-9.09e-02	2.22e-02	-3.69e-02	1.81e-02	-5.86e-02	1.40e-02	-6.75e-02	5.33e-02	1.62e-02
-7.52e-02	5.17e-02	2.95e-02	4.04e-02	-1.10e-02	3.18e-02	-2.17e-01	1.33e-01	5.44e-02
-5.98e-02	4.11e-02	-1.44e-02	3.32e-02	-3.22e-02	2.58e-02	5.86e-02	9.46e-02	-3.82e-02
-5.26e-03	2.16e-02	1.92e-03	1.77e-02	-8.37e-04	1.37e-02	1.54e-02	5.19e-02	8.51e-02
-1.23e-02	2.24e-02	-1.76e-02	1.84e-02	-1.55e-02	1.42e-02	-8.69e-02	5.32e-02	-5.93e-02
1.22e-02	2.20e-02	4.29e-03	1.81e-02	7.65e-03	1.40e-02	9.84e-02	5.24e-02	8.72e-03
1.79e-02	2.20e-02	-8.30e-04	1.81e-02	6.59e-03	1.40e-02	1.25e-02	5.31e-02	-4.27e-02
2.81e-02	2.10e-02	-1.79e-02	1.72e-02	5.64e-04	1.33e-02	-8.76e-02	5.04e-02	-2.54e-02
2.62e-02	2.11e-02	1.22e-02	1.73e-02	1.77e-02	1.34e-02	1.37e-01	5.13e-02	1.19e-02
-9.76e-03	2.65e-02	-1.87e-02	2.17e-02	-1.53e-02	1.68e-02	5.43e-02	6.28e-02	-1.71e-02
-8.51e-03	2.34e-02	-2.28e-03	1.92e-02	-4.62e-03	1.49e-02	2.86e-02	5.70e-02	5.88e-02
-3.70e-03	2.14e-02	-1.36e-02	1.75e-02	-9.49e-03	1.36e-02	4.51e-03	5.17e-02	1.19e-01
3.05e-03	2.17e-02	-2.47e-02	1.77e-02	-1.33e-02	1.37e-02	4.87e-02	5.27e-02	3.11e-02
5.63e-02	2.42e-02	9.71e-03	1.97e-02	2.82e-02	1.53e-02	7.04e-02	5.87e-02	1.48e-02
3.35e-02	2.09e-02	-6.00e-03	1.72e-02	9.69e-03	1.33e-02	-3.01e-02	5.03e-02	7.02e-02
1.50e-02	2.10e-02	-9.23e-03	1.71e-02	4.12e-04	1.33e-02	-6.09e-05	5.06e-02	4.18e-02
-1.83e-03	2.09e-02	1.17e-02	1.71e-02	6.15e-03	1.32e-02	7.36e-02	5.03e-02	5.21e-02
-3.78e-02	2.09e-02	1.88e-02	1.71e-02	-4.07e-03	1.33e-02	-6.81e-02	5.06e-02	-2.37e-04
3.14e-02	2.08e-02	-1.75e-02	1.71e-02	2.08e-03	1.32e-02	-3.92e-02	5.04e-02	-6.69e-02
-2.58e-02	2.82e-02	-6.29e-02	2.29e-02	-4.79e-02	1.78e-02	-2.32e-02	6.80e-02	2.40e-02
-1.82e-02	2.15e-02	-6.35e-05	1.76e-02	-7.25e-03	1.36e-02	4.21e-02	5.21e-02	-2.00e-02
-2.36e-02	2.16e-02	7.88e-03	1.77e-02	-4.74e-03	1.37e-02	-3.38e-03	5.22e-02	-3.29e-02
-2.22e-02	2.48e-02	6.87e-03	2.02e-02	-4.48e-03	1.57e-02	-6.75e-03	5.97e-02	-2.30e-02
-3.28e-02	2.52e-02	-3.01e-02	2.07e-02	-3.08e-02	1.60e-02	-7.37e-02	6.15e-02	-6.94e-02
-5.38e-03	2.29e-02	2.38e-02	1.88e-02	1.20e-02	1.45e-02	-6.16e-03	5.52e-02	1.58e-02
-3.77e-02	2.09e-02	2.16e-02	1.71e-02	-2.27e-03	1.32e-02	6.28e-02	5.01e-02	-2.01e-02
-9.52e-03	2.09e-02	2.77e-02	1.72e-02	1.26e-02	1.33e-02	-1.38e-02	5.05e-02	-2.00e-02
4.20e-03	4.94e-02	5.73e-02	3.95e-02	3.62e-02	3.08e-02	-7.67e-02	1.23e-01	6.28e-02

-1.72e-02	2.78e-02	2.18e-02	2.30e-02	6.37e-03	1.77e-02	-1.82e-02	6.70e-02	1.45e-01
1.64e-02	2.13e-02	1.98e-02	1.74e-02	1.82e-02	1.35e-02	-5.03e-02	5.15e-02	4.79e-02
3.05e-02	2.09e-02	2.45e-02	1.71e-02	2.69e-02	1.32e-02	-5.90e-02	5.03e-02	-8.21e-03
-8.66e-03	2.28e-02	9.86e-04	1.88e-02	-2.84e-03	1.45e-02	-3.61e-02	5.47e-02	-3.21e-02
3.98e-02	2.28e-02	1.68e-03	1.88e-02	1.70e-02	1.45e-02	5.37e-02	5.48e-02	7.06e-02
-7.67e-02	2.65e-02	-8.16e-04	2.13e-02	-3.11e-02	1.66e-02	1.43e-02	6.22e-02	1.52e-02
1.20e-02	3.64e-02	5.30e-03	2.99e-02	8.08e-03	2.31e-02	1.29e-01	8.38e-02	8.15e-02
2.14e-02	2.49e-02	1.36e-02	2.05e-02	1.69e-02	1.58e-02	5.36e-02	5.94e-02	-2.63e-02
-1.07e-02	2.10e-02	-2.29e-02	1.72e-02	-1.80e-02	1.33e-02	-5.15e-02	5.05e-02	-1.33e-02
-3.33e-04	2.10e-02	1.65e-02	1.72e-02	9.60e-03	1.33e-02	3.71e-02	5.06e-02	6.41e-02
-2.21e-02	3.09e-02	-2.60e-02	2.53e-02	-2.44e-02	1.96e-02	1.63e-02	7.34e-02	-3.51e-02
6.35e-04	2.97e-02	7.14e-02	2.37e-02	4.33e-02	1.85e-02	-3.12e-02	7.24e-02	-4.73e-02
6.43e-03	2.16e-02	-1.19e-02	1.77e-02	-4.63e-03	1.37e-02	-5.08e-02	5.24e-02	5.36e-02
1.29e-02	2.14e-02	1.04e-02	1.76e-02	1.15e-02	1.36e-02	-6.44e-02	5.21e-02	3.69e-02
-1.53e-02	2.09e-02	-1.39e-02	1.71e-02	-1.45e-02	1.32e-02	3.05e-02	5.05e-02	6.13e-02
3.15e-05	2.20e-02	3.50e-02	1.80e-02	2.11e-02	1.39e-02	-9.23e-02	5.40e-02	-5.26e-03
2.66e-02	2.35e-02	-3.22e-02	1.95e-02	-8.51e-03	1.50e-02	2.48e-02	5.67e-02	-4.34e-02
-3.74e-02	2.11e-02	2.37e-02	1.74e-02	-1.00e-03	1.34e-02	3.95e-02	5.13e-02	9.02e-02
-4.85e-03	2.84e-02	5.43e-03	2.32e-02	1.41e-03	1.80e-02	3.69e-03	6.83e-02	-5.22e-02
2.04e-02	3.66e-02	-2.47e-03	3.02e-02	6.41e-03	2.33e-02	8.50e-02	8.61e-02	-7.37e-02
2.21e-04	2.11e-02	1.43e-02	1.72e-02	8.61e-03	1.33e-02	5.81e-02	5.06e-02	-2.43e-02
2.86e-02	2.09e-02	-1.91e-02	1.72e-02	2.89e-04	1.33e-02	5.37e-02	5.02e-02	-1.45e-01
-1.62e-02	2.45e-02	9.37e-03	2.00e-02	-8.51e-04	1.55e-02	3.77e-03	5.89e-02	1.29e-02
2.91e-02	2.14e-02	3.61e-02	1.75e-02	3.33e-02	1.35e-02	6.58e-02	5.13e-02	7.72e-02
1.78e-02	4.84e-02	-4.65e-02	4.06e-02	-2.05e-02	3.11e-02	5.03e-02	1.15e-01	-1.95e-01
1.41e-02	4.76e-02	-6.52e-02	4.01e-02	-3.32e-02	3.07e-02	5.52e-02	1.13e-01	3.17e-02
-1.94e-03	2.10e-02	2.39e-02	1.73e-02	1.35e-02	1.33e-02	3.22e-02	5.08e-02	1.06e-02
1.51e-03	2.17e-02	1.43e-03	1.77e-02	1.33e-03	1.37e-02	-1.29e-03	5.23e-02	1.69e-04
2.74e-02	6.85e-02	-1.12e-02	5.74e-02	5.01e-03	4.40e-02	-2.93e-01	1.90e-01	-2.29e-01
-9.04e-03	3.04e-02	-7.84e-03	2.49e-02	-8.30e-03	1.92e-02	3.49e-02	7.22e-02	-8.94e-02
-8.16e-03	3.65e-02	1.50e-02	2.97e-02	5.82e-03	2.30e-02	-6.47e-02	9.01e-02	7.66e-02
6.55e-03	2.99e-02	2.37e-02	2.44e-02	1.67e-02	1.89e-02	-1.16e-02	7.25e-02	-6.99e-02
1.06e-02	2.65e-02	-4.61e-02	2.20e-02	-2.30e-02	1.69e-02	-1.03e-01	6.61e-02	-8.87e-03
-3.45e-02	2.51e-02	-4.92e-03	2.04e-02	-1.63e-02	1.59e-02	6.72e-02	5.92e-02	5.37e-02
-1.71e-02	2.11e-02	3.09e-02	1.72e-02	1.15e-02	1.33e-02	1.69e-02	5.07e-02	-1.20e-01
4.04e-02	2.17e-02	1.36e-02	1.79e-02	2.44e-02	1.38e-02	-1.20e-01	5.36e-02	1.40e-02
-1.40e-02	2.30e-02	-2.64e-02	1.88e-02	-2.13e-02	1.46e-02	-3.44e-02	5.52e-02	-4.72e-02
6.17e-02	2.08e-02	1.51e-02	1.71e-02	3.38e-02	1.32e-02	-9.40e-02	5.01e-02	4.03e-03
-3.41e-02	2.08e-02	3.82e-02	1.71e-02	9.16e-03	1.32e-02	-3.90e-04	5.03e-02	1.92e-02
-5.50e-03	2.09e-02	1.66e-02	1.72e-02	7.61e-03	1.33e-02	8.39e-02	5.03e-02	-7.78e-02
-1.22e-02	2.13e-02	2.56e-02	1.74e-02	1.05e-02	1.35e-02	-4.67e-02	5.15e-02	1.99e-02
-1.88e-02	2.12e-02	-2.90e-02	1.74e-02	-2.49e-02	1.34e-02	6.22e-03	5.11e-02	-7.51e-02
-2.01e-02	3.33e-02	-3.33e-02	2.74e-02	-2.80e-02	2.12e-02	2.16e-02	8.14e-02	-1.04e-01
-1.14e-03	2.22e-02	1.33e-02	1.81e-02	7.52e-03	1.40e-02	-6.36e-03	5.36e-02	-5.10e-02
-2.84e-02	2.73e-02	1.71e-02	2.26e-02	-1.11e-03	1.74e-02	6.22e-02	6.77e-02	-2.58e-02
-1.37e-02	2.75e-02	4.36e-02	2.21e-02	2.07e-02	1.72e-02	3.69e-02	6.53e-02	2.17e-02
-2.47e-02	2.11e-02	-1.61e-02	1.73e-02	-1.98e-02	1.34e-02	2.56e-02	5.10e-02	3.96e-02

-3.79e-03	2.12e-02	1.74e-02	1.72e-02	9.01e-03	1.34e-02	-7.78e-02	5.14e-02	5.08e-02
6.05e-03	2.13e-02	-1.34e-02	1.75e-02	-5.41e-03	1.36e-02	1.61e-02	5.14e-02	2.32e-02
1.46e-02	2.08e-02	-1.65e-02	1.70e-02	-3.86e-03	1.32e-02	-3.01e-02	5.01e-02	-1.54e-02
-2.12e-02	2.27e-02	3.86e-03	1.85e-02	-6.40e-03	1.43e-02	-6.36e-02	5.51e-02	-2.49e-02
2.94e-02	2.12e-02	-3.33e-02	1.72e-02	-8.28e-03	1.34e-02	-5.22e-03	5.10e-02	-3.61e-02
-2.51e-02	2.11e-02	2.05e-02	1.72e-02	2.16e-03	1.34e-02	7.11e-03	5.07e-02	3.71e-02
-1.19e-04	2.13e-02	-1.06e-02	1.75e-02	-6.02e-03	1.35e-02	-2.10e-02	5.15e-02	5.01e-02
4.84e-02	2.52e-02	2.86e-02	2.07e-02	3.68e-02	1.60e-02	3.44e-02	6.11e-02	-8.11e-02
4.99e-02	3.87e-02	3.10e-02	3.19e-02	3.81e-02	2.46e-02	-6.93e-02	9.77e-02	6.27e-02
-8.45e-03	3.29e-02	6.28e-02	2.65e-02	3.43e-02	2.06e-02	6.96e-02	7.71e-02	-1.03e-01
-2.52e-03	3.36e-02	-1.22e-02	2.76e-02	-8.28e-03	2.13e-02	6.08e-03	8.08e-02	7.84e-02
-4.58e-02	2.12e-02	2.12e-02	1.73e-02	-5.76e-03	1.34e-02	-3.42e-02	5.10e-02	2.03e-02
-4.55e-03	2.10e-02	1.89e-02	1.72e-02	9.51e-03	1.33e-02	-4.20e-02	5.08e-02	5.32e-02
-5.75e-02	2.14e-02	-3.13e-02	1.75e-02	-4.19e-02	1.35e-02	-4.03e-02	5.16e-02	-5.43e-02
2.71e-02	3.07e-02	4.30e-02	2.50e-02	3.67e-02	1.94e-02	-3.17e-02	7.56e-02	5.23e-02
2.39e-03	2.81e-02	-1.35e-02	2.32e-02	-6.88e-03	1.79e-02	-1.49e-01	7.14e-02	-6.99e-03
-1.75e-02	2.21e-02	1.27e-02	1.81e-02	5.57e-04	1.40e-02	-8.30e-02	5.40e-02	-6.93e-02
1.31e-02	2.90e-02	-6.33e-02	2.43e-02	-3.25e-02	1.86e-02	-1.27e-01	7.36e-02	-1.50e-01
-2.97e-02	3.05e-02	-1.05e-02	2.49e-02	-1.81e-02	1.93e-02	6.81e-02	7.11e-02	-1.22e-02
-2.62e-02	2.54e-02	3.21e-02	2.04e-02	8.90e-03	1.59e-02	5.14e-02	6.00e-02	-1.96e-02
-3.34e-02	2.87e-02	-3.77e-02	2.35e-02	-3.59e-02	1.82e-02	-7.82e-02	7.02e-02	4.22e-03
6.15e-02	3.20e-02	-5.79e-03	2.68e-02	2.18e-02	2.05e-02	2.44e-02	7.80e-02	-1.94e-03
1.13e-02	2.58e-02	2.20e-02	2.12e-02	1.78e-02	1.64e-02	5.19e-03	6.21e-02	2.57e-03
1.98e-02	2.08e-02	3.00e-02	1.71e-02	2.60e-02	1.32e-02	2.00e-02	5.02e-02	-8.54e-03
-7.26e-03	2.10e-02	2.72e-02	1.72e-02	1.34e-02	1.33e-02	3.38e-02	5.07e-02	-2.38e-02
5.05e-02	2.10e-02	2.34e-02	1.72e-02	3.42e-02	1.33e-02	1.97e-02	5.06e-02	-1.48e-02
-2.14e-02	4.02e-02	3.18e-02	3.24e-02	1.08e-02	2.52e-02	3.11e-02	9.49e-02	-4.26e-03
2.06e-02	2.26e-02	-4.42e-03	1.86e-02	5.67e-03	1.44e-02	3.85e-02	5.43e-02	7.44e-03
-1.91e-02	2.51e-02	-6.18e-03	2.05e-02	-1.12e-02	1.59e-02	1.13e-01	5.85e-02	-4.01e-02
-5.01e-02	5.25e-02	-9.42e-03	4.18e-02	-2.55e-02	3.27e-02	-1.39e-02	1.25e-01	-6.77e-02
-2.11e-02	2.15e-02	-2.66e-02	1.76e-02	-2.44e-02	1.36e-02	-1.02e-01	5.24e-02	-2.33e-02
-3.21e-02	2.19e-02	4.07e-03	1.78e-02	-1.03e-02	1.38e-02	2.34e-03	5.25e-02	-2.98e-02
7.27e-03	2.64e-02	2.07e-02	2.16e-02	1.54e-02	1.67e-02	4.12e-02	6.29e-02	-2.01e-02
-1.46e-02	2.22e-02	1.33e-03	1.82e-02	-5.11e-03	1.41e-02	-2.73e-02	5.34e-02	-5.21e-02
2.12e-02	2.52e-02	4.09e-02	2.04e-02	3.29e-02	1.59e-02	2.04e-02	6.07e-02	-4.83e-02
-2.00e-02	2.90e-02	-5.83e-02	2.40e-02	-4.26e-02	1.85e-02	-1.98e-02	6.99e-02	7.49e-03
7.08e-03	2.09e-02	-6.05e-03	1.71e-02	-6.60e-04	1.32e-02	3.37e-02	5.03e-02	-2.03e-02
2.89e-02	3.07e-02	1.62e-02	2.55e-02	2.14e-02	1.96e-02	-4.97e-02	7.63e-02	-4.90e-02
3.90e-03	2.08e-02	1.84e-02	1.71e-02	1.24e-02	1.32e-02	5.51e-02	5.03e-02	-7.05e-02
-1.25e-02	2.19e-02	-1.06e-02	1.79e-02	-1.12e-02	1.38e-02	-2.01e-01	5.42e-02	-5.77e-02
5.06e-04	2.40e-02	3.02e-02	1.96e-02	1.83e-02	1.52e-02	-9.05e-03	5.80e-02	-1.13e-02
2.55e-02	2.72e-02	-3.22e-02	2.27e-02	-8.80e-03	1.74e-02	4.42e-02	6.51e-02	4.55e-02
-1.73e-02	2.10e-02	-3.39e-02	1.72e-02	-2.72e-02	1.33e-02	-2.65e-02	5.06e-02	-6.77e-02
4.10e-02	2.55e-02	3.82e-03	2.06e-02	1.88e-02	1.60e-02	-6.36e-02	5.97e-02	-4.34e-02
-2.11e-02	2.27e-02	-7.68e-03	1.86e-02	-1.28e-02	1.44e-02	4.92e-02	5.40e-02	7.46e-02
1.68e-02	2.10e-02	2.26e-03	1.72e-02	8.03e-03	1.33e-02	-3.96e-02	5.09e-02	-2.26e-02
1.29e-02	2.12e-02	1.24e-03	1.74e-02	5.98e-03	1.35e-02	2.01e-02	5.12e-02	1.14e-02

-2.32e-02	2.32e-02	1.18e-02	1.91e-02	-1.99e-03	1.47e-02	1.59e-02	5.63e-02	4.56e-03
1.84e-02	2.17e-02	2.54e-02	1.77e-02	2.25e-02	1.37e-02	-5.51e-03	5.25e-02	6.37e-03
-2.14e-02	2.26e-02	-6.79e-03	1.87e-02	-1.29e-02	1.44e-02	-3.73e-02	5.43e-02	3.98e-03
-7.56e-03	2.23e-02	-1.45e-02	1.83e-02	-1.15e-02	1.41e-02	-4.89e-02	5.42e-02	-4.63e-02
2.16e-03	2.22e-02	2.77e-02	1.81e-02	1.76e-02	1.40e-02	-8.17e-02	5.44e-02	5.40e-03
2.70e-04	2.34e-02	-3.25e-02	1.94e-02	-1.94e-02	1.49e-02	2.76e-02	5.61e-02	-2.15e-02
-5.71e-03	2.39e-02	7.74e-03	1.97e-02	2.21e-03	1.52e-02	7.72e-02	5.87e-02	2.12e-02
-3.41e-02	2.10e-02	-6.18e-03	1.73e-02	-1.76e-02	1.34e-02	-8.42e-02	5.06e-02	-6.51e-02
-5.24e-03	2.42e-02	3.62e-02	1.96e-02	1.95e-02	1.52e-02	5.56e-02	5.75e-02	2.91e-02
3.81e-02	2.34e-02	-1.37e-02	1.90e-02	7.40e-03	1.47e-02	1.54e-02	5.62e-02	-7.16e-02
3.68e-02	2.12e-02	-1.86e-03	1.74e-02	1.38e-02	1.35e-02	-3.40e-04	5.13e-02	5.51e-02
1.17e-02	2.29e-02	-1.41e-02	1.89e-02	-3.57e-03	1.45e-02	-1.09e-01	5.66e-02	6.82e-03
-8.87e-03	2.10e-02	6.13e-03	1.72e-02	1.64e-04	1.33e-02	-9.89e-02	5.03e-02	1.42e-01
7.71e-03	2.09e-02	1.39e-03	1.71e-02	3.96e-03	1.32e-02	8.86e-03	5.04e-02	8.75e-02
2.43e-02	2.33e-02	1.56e-02	1.92e-02	1.91e-02	1.48e-02	2.13e-02	5.63e-02	5.57e-03
4.54e-02	2.26e-02	4.79e-03	1.84e-02	2.11e-02	1.43e-02	-2.54e-03	5.42e-02	1.80e-03
-2.64e-02	2.22e-02	-2.18e-02	1.82e-02	-2.38e-02	1.41e-02	-5.31e-02	5.34e-02	5.43e-02
9.51e-03	2.15e-02	1.29e-02	1.76e-02	1.13e-02	1.36e-02	4.24e-02	5.20e-02	-5.24e-02
-7.25e-03	2.09e-02	-9.80e-03	1.72e-02	-8.88e-03	1.33e-02	5.63e-02	5.04e-02	-8.38e-02
-1.70e-02	2.08e-02	1.72e-02	1.70e-02	3.76e-03	1.32e-02	-5.20e-02	5.01e-02	4.70e-02
-3.10e-03	2.27e-02	-3.08e-02	1.85e-02	-1.96e-02	1.44e-02	-1.99e-02	5.46e-02	-4.07e-02
8.03e-02	3.77e-02	-5.09e-02	3.25e-02	3.40e-03	2.47e-02	-1.00e-01	9.79e-02	-4.64e-02
-3.11e-02	2.33e-02	-1.15e-02	1.92e-02	-1.95e-02	1.48e-02	-9.73e-02	5.54e-02	3.88e-02
-1.58e-02	2.10e-02	-8.31e-03	1.72e-02	-1.13e-02	1.33e-02	2.74e-02	5.08e-02	-1.83e-02
1.76e-02	2.13e-02	1.54e-02	1.75e-02	1.64e-02	1.35e-02	-3.87e-02	5.17e-02	-6.95e-02
-2.00e-02	2.21e-02	2.23e-02	1.83e-02	5.31e-03	1.41e-02	1.00e-01	5.44e-02	2.27e-02
-4.48e-02	1.10e-01	2.80e-03	8.55e-02	-1.49e-02	6.74e-02	-2.07e-02	2.63e-01	5.19e-02
5.80e-03	2.08e-02	1.10e-02	1.70e-02	8.79e-03	1.32e-02	-4.77e-02	5.01e-02	-1.55e-02
3.75e-02	2.31e-02	2.68e-02	1.89e-02	3.09e-02	1.46e-02	1.06e-01	5.50e-02	6.91e-02
6.35e-03	2.12e-02	-1.38e-02	1.74e-02	-5.79e-03	1.34e-02	1.72e-03	5.11e-02	-2.07e-02
-3.81e-02	2.48e-02	1.38e-03	2.06e-02	-1.46e-02	1.58e-02	1.55e-02	6.05e-02	3.50e-02
4.02e-02	2.12e-02	-3.73e-02	1.72e-02	-6.29e-03	1.33e-02	-6.93e-03	5.08e-02	-1.91e-02

ICH male SE	H overall Be	CH overall S	H female B	AH female S	AH male Be	SAH male S	H overall B	AH overall S
4.65e-02	9.97e-03	3.43e-02	-7.97e-02	4.99e-02	-2.43e-03	6.21e-02	-4.98e-02	3.89e-02
4.52e-02	2.16e-02	3.37e-02	5.92e-02	4.86e-02	-7.33e-03	6.07e-02	3.28e-02	3.80e-02
4.62e-02	4.61e-02	3.46e-02	3.93e-02	4.98e-02	6.29e-02	6.27e-02	4.85e-02	3.90e-02
4.62e-02	-2.77e-02	3.45e-02	5.73e-03	4.95e-02	-1.04e-01	6.31e-02	-3.66e-02	3.90e-02
4.50e-02	4.09e-03	3.35e-02	1.24e-02	4.82e-02	-8.08e-02	6.05e-02	-2.37e-02	3.77e-02
4.51e-02	1.21e-02	3.36e-02	3.17e-02	4.83e-02	-1.10e-01	6.08e-02	-2.37e-02	3.78e-02
4.84e-02	2.71e-02	3.59e-02	-5.68e-02	5.09e-02	2.72e-02	6.49e-02	-2.49e-02	4.00e-02
4.50e-02	1.05e-02	3.35e-02	4.18e-02	4.82e-02	-3.49e-02	6.05e-02	1.23e-02	3.77e-02
4.89e-02	2.57e-02	3.58e-02	1.02e-02	5.16e-02	1.51e-02	6.50e-02	1.21e-02	4.04e-02
4.66e-02	4.64e-02	3.47e-02	-3.74e-02	4.95e-02	9.57e-02	6.32e-02	1.38e-02	3.90e-02
4.75e-02	7.08e-02	3.54e-02	7.02e-02	5.10e-02	-4.17e-02	6.51e-02	2.69e-02	4.01e-02
4.62e-02	-3.96e-02	3.43e-02	6.11e-03	4.92e-02	1.66e-02	6.17e-02	1.05e-02	3.85e-02
5.54e-02	-2.73e-02	4.15e-02	3.46e-02	5.87e-02	5.18e-02	7.32e-02	4.15e-02	4.58e-02
4.55e-02	-4.38e-03	3.39e-02	-2.91e-02	4.88e-02	-9.19e-02	6.10e-02	-5.35e-02	3.81e-02
9.79e-02	-7.41e-02	7.41e-02	-5.87e-02	1.06e-01	-2.12e-02	1.31e-01	-4.42e-02	8.23e-02
4.71e-02	1.71e-02	3.50e-02	-7.76e-03	5.02e-02	1.00e-01	6.42e-02	3.41e-02	3.96e-02
4.76e-02	-4.37e-03	3.55e-02	6.00e-02	5.06e-02	-5.80e-02	6.45e-02	1.47e-02	3.98e-02
5.80e-02	1.20e-02	4.32e-02	-2.98e-02	6.31e-02	1.67e-02	7.79e-02	-1.14e-02	4.90e-02
4.56e-02	4.98e-02	3.41e-02	1.03e-02	4.93e-02	-6.82e-02	6.23e-02	-2.02e-02	3.86e-02
5.25e-02	7.32e-02	3.87e-02	7.28e-02	5.57e-02	1.97e-02	7.11e-02	5.22e-02	4.38e-02
5.82e-02	-6.39e-02	4.39e-02	9.67e-02	6.03e-02	-1.18e-01	8.06e-02	1.63e-02	4.82e-02
4.62e-02	1.95e-02	3.42e-02	2.79e-02	4.94e-02	-9.64e-02	6.12e-02	-2.06e-02	3.84e-02
4.94e-02	8.15e-03	3.65e-02	9.44e-02	5.17e-02	2.24e-02	6.59e-02	6.65e-02	4.07e-02
4.63e-02	-2.00e-02	3.43e-02	-4.55e-04	4.95e-02	6.41e-02	6.25e-02	2.44e-02	3.88e-02
4.55e-02	3.20e-02	3.39e-02	-2.54e-04	4.89e-02	-4.14e-02	6.16e-02	-1.57e-02	3.83e-02
5.80e-02	-2.37e-02	4.30e-02	1.08e-01	5.98e-02	7.91e-02	7.53e-02	9.67e-02	4.68e-02
6.03e-02	8.98e-03	4.52e-02	-3.05e-02	6.60e-02	-3.56e-02	8.27e-02	-3.21e-02	5.16e-02
4.52e-02	5.32e-02	3.37e-02	-8.32e-03	4.84e-02	1.13e-01	6.11e-02	3.88e-02	3.79e-02
4.68e-02	4.53e-02	3.47e-02	-3.23e-02	5.05e-02	1.33e-02	6.28e-02	-1.47e-02	3.94e-02
4.92e-02	-4.93e-02	3.67e-02	-6.20e-02	5.32e-02	1.20e-02	6.55e-02	-3.26e-02	4.13e-02
7.29e-02	3.25e-03	5.50e-02	-1.29e-01	8.38e-02	7.06e-02	9.67e-02	-4.75e-02	6.33e-02
5.61e-02	3.97e-02	4.28e-02	6.46e-02	6.11e-02	5.20e-02	7.69e-02	6.04e-02	4.78e-02
4.58e-02	-3.81e-02	3.43e-02	-6.78e-02	4.92e-02	4.64e-02	6.26e-02	-2.38e-02	3.87e-02
6.25e-02	-1.50e-02	4.55e-02	2.12e-02	6.48e-02	-1.08e-02	8.22e-02	9.06e-03	5.09e-02
4.58e-02	-3.15e-02	3.42e-02	-8.43e-02	4.95e-02	-6.67e-02	6.18e-02	-7.70e-02	3.87e-02
4.51e-02	-3.63e-02	3.36e-02	-4.61e-02	4.84e-02	1.66e-03	6.07e-02	-2.81e-02	3.79e-02
7.11e-02	-6.84e-03	5.33e-02	1.82e-01	7.15e-02	1.04e-01	9.22e-02	1.52e-01	5.65e-02
8.92e-02	6.95e-02	6.82e-02	-2.92e-02	1.02e-01	1.61e-02	1.26e-01	-1.09e-02	7.94e-02
8.33e-02	1.06e-01	6.07e-02	6.15e-02	8.95e-02	1.01e-01	1.09e-01	7.66e-02	6.92e-02
4.73e-02	4.19e-02	3.49e-02	6.36e-02	5.02e-02	-1.14e-05	6.35e-02	3.92e-02	3.94e-02
5.96e-02	-1.98e-03	4.46e-02	3.61e-02	6.36e-02	1.06e-01	7.77e-02	6.35e-02	4.92e-02
4.49e-02	6.04e-03	3.35e-02	5.40e-02	4.83e-02	3.50e-02	6.05e-02	4.64e-02	3.77e-02
5.30e-02	2.73e-02	3.93e-02	2.89e-03	5.69e-02	-5.56e-02	7.25e-02	-1.90e-02	4.47e-02
4.53e-02	-3.76e-02	3.38e-02	1.16e-02	4.89e-02	3.64e-02	6.15e-02	2.13e-02	3.82e-02
7.23e-02	4.74e-02	5.19e-02	6.97e-02	7.37e-02	1.89e-01	8.90e-02	1.17e-01	5.68e-02
4.88e-02	-3.41e-02	3.60e-02	1.03e-01	5.07e-02	-1.38e-01	6.62e-02	1.13e-02	4.02e-02

4.50e-02	6.76e-02	3.35e-02	-1.03e-02	4.84e-02	4.98e-02	6.05e-02	1.31e-02	3.78e-02
7.64e-02	-7.38e-02	5.61e-02	-1.09e-01	8.18e-02	-1.12e-01	1.03e-01	-1.11e-01	6.40e-02
5.80e-02	-1.14e-02	4.35e-02	-7.39e-02	6.16e-02	-1.78e-03	7.87e-02	-4.60e-02	4.85e-02
5.24e-02	-3.65e-02	3.90e-02	2.93e-02	5.53e-02	2.56e-02	6.94e-02	2.74e-02	4.32e-02
4.49e-02	-7.50e-03	3.34e-02	9.76e-02	4.81e-02	-8.53e-02	6.05e-02	2.68e-02	3.76e-02
5.22e-02	1.28e-03	3.88e-02	2.40e-05	5.58e-02	-1.64e-02	7.04e-02	-5.95e-03	4.37e-02
6.96e-02	4.48e-02	5.19e-02	1.14e-01	7.28e-02	4.43e-02	9.37e-02	8.79e-02	5.75e-02
6.56e-02	-2.32e-02	5.02e-02	1.23e-01	6.86e-02	1.41e-01	8.53e-02	1.30e-01	5.34e-02
5.18e-02	-4.98e-02	3.87e-02	4.01e-02	5.46e-02	-1.29e-01	7.13e-02	-2.34e-02	4.33e-02
5.99e-02	1.25e-03	4.55e-02	-3.99e-02	6.66e-02	9.66e-02	7.96e-02	1.43e-02	5.11e-02
1.04e-01	-4.99e-02	7.61e-02	9.60e-02	1.02e-01	1.91e-01	1.24e-01	1.34e-01	7.89e-02
4.73e-02	-8.17e-03	3.51e-02	-2.28e-02	5.04e-02	-3.45e-02	6.33e-02	-2.73e-02	3.94e-02
4.87e-02	-1.22e-02	3.58e-02	-4.99e-02	5.13e-02	-1.60e-02	6.47e-02	-3.67e-02	4.02e-02
5.76e-02	4.48e-02	4.34e-02	-9.98e-02	6.56e-02	6.54e-02	7.79e-02	-3.39e-02	5.02e-02
6.71e-02	1.25e-02	5.00e-02	8.29e-02	7.04e-02	1.69e-01	8.54e-02	1.17e-01	5.43e-02
4.84e-02	5.59e-02	3.65e-02	3.66e-02	5.28e-02	4.61e-03	6.66e-02	2.45e-02	4.14e-02
4.51e-02	-3.74e-03	3.37e-02	-2.62e-02	4.86e-02	1.44e-03	6.08e-02	-1.56e-02	3.79e-02
5.05e-02	4.22e-02	3.77e-02	1.63e-02	5.47e-02	-2.17e-02	6.88e-02	1.38e-03	4.28e-02
4.73e-02	-1.29e-02	3.53e-02	1.01e-02	5.07e-02	-9.63e-03	6.37e-02	2.02e-03	3.97e-02
4.51e-02	1.52e-02	3.36e-02	-4.03e-02	4.83e-02	-8.92e-02	6.06e-02	-5.89e-02	3.78e-02
5.97e-02	-8.65e-02	4.38e-02	-1.30e-02	6.14e-02	-8.11e-02	7.93e-02	-3.84e-02	4.85e-02
4.94e-02	-2.05e-04	3.64e-02	-8.75e-03	5.23e-02	1.45e-01	6.77e-02	4.98e-02	4.14e-02
6.67e-02	5.69e-02	5.06e-02	-7.46e-02	7.67e-02	-3.02e-02	9.40e-02	-5.68e-02	5.94e-02
4.78e-02	-4.25e-02	3.58e-02	-8.02e-02	5.20e-02	2.80e-02	6.39e-02	-3.75e-02	4.03e-02
4.76e-02	1.25e-02	3.54e-02	7.82e-02	5.16e-02	-4.99e-02	6.32e-02	2.76e-02	3.99e-02
5.12e-02	-3.16e-02	3.78e-02	-1.26e-02	5.46e-02	7.03e-02	7.00e-02	1.92e-02	4.30e-02
9.88e-02	-3.54e-03	7.46e-02	3.60e-03	1.07e-01	2.17e-01	1.23e-01	9.16e-02	8.06e-02
6.20e-02	2.36e-02	4.69e-02	2.95e-02	6.74e-02	1.06e-01	8.24e-02	5.98e-02	5.22e-02
4.50e-02	2.41e-02	3.35e-02	8.17e-02	4.84e-02	3.53e-02	6.05e-02	6.38e-02	3.78e-02
4.76e-02	-4.15e-02	3.53e-02	6.33e-02	5.18e-02	-4.32e-02	6.38e-02	2.17e-02	4.02e-02
4.96e-02	3.79e-02	3.71e-02	-3.49e-02	5.44e-02	6.13e-03	6.73e-02	-1.85e-02	4.23e-02
4.52e-02	3.52e-02	3.36e-02	7.92e-02	4.85e-02	-1.53e-02	6.08e-02	4.29e-02	3.79e-02
5.44e-02	3.51e-02	4.00e-02	8.72e-02	5.68e-02	-7.65e-03	7.31e-02	5.10e-02	4.48e-02
4.74e-02	-1.78e-02	3.54e-02	7.82e-02	5.19e-02	3.35e-02	6.44e-02	6.11e-02	4.04e-02
4.82e-02	4.36e-02	3.59e-02	5.41e-04	5.13e-02	8.44e-03	6.46e-02	3.44e-03	4.02e-02
4.49e-02	9.81e-03	3.35e-02	-4.22e-02	4.83e-02	5.15e-02	6.04e-02	-6.00e-03	3.77e-02
4.61e-02	2.13e-02	3.44e-02	-2.37e-02	4.98e-02	6.93e-02	6.17e-02	1.28e-02	3.87e-02
4.74e-02	5.92e-02	3.53e-02	3.93e-02	5.09e-02	5.84e-02	6.38e-02	4.69e-02	3.98e-02
4.84e-02	-7.36e-02	3.60e-02	1.15e-02	5.10e-02	1.32e-01	6.29e-02	5.85e-02	3.96e-02
4.52e-02	1.43e-03	3.37e-02	5.00e-02	4.85e-02	1.13e-02	6.09e-02	3.54e-02	3.79e-02
5.55e-02	4.15e-02	4.05e-02	-1.43e-01	5.56e-02	-1.15e-01	7.02e-02	-1.33e-01	4.36e-02
4.77e-02	-1.58e-03	3.56e-02	6.99e-03	5.11e-02	-6.08e-02	6.37e-02	-1.94e-02	3.99e-02
5.17e-02	-2.42e-02	3.86e-02	2.67e-02	5.49e-02	-4.23e-02	7.00e-02	-2.34e-04	4.32e-02
4.65e-02	-8.76e-03	3.47e-02	-6.50e-02	5.04e-02	-1.73e-02	6.27e-02	-4.62e-02	3.93e-02
5.21e-02	4.71e-02	3.84e-02	4.81e-02	5.54e-02	9.95e-02	6.87e-02	6.78e-02	4.31e-02
4.51e-02	-4.44e-03	3.36e-02	-5.29e-02	4.84e-02	-5.11e-02	6.07e-02	-5.19e-02	3.78e-02
5.10e-02	6.16e-02	3.79e-02	-5.54e-02	5.62e-02	-4.23e-02	7.01e-02	-5.02e-02	4.38e-02

4.62e-02	1.06e-02	3.44e-02	7.45e-02	4.93e-02	4.29e-03	6.20e-02	4.77e-02	3.86e-02
4.54e-02	-6.95e-03	3.38e-02	-3.82e-02	4.86e-02	9.67e-02	6.09e-02	1.41e-02	3.80e-02
4.53e-02	5.03e-02	3.38e-02	9.07e-02	4.89e-02	-8.11e-03	6.09e-02	5.27e-02	3.81e-02
4.53e-02	-7.37e-04	3.38e-02	3.91e-02	4.87e-02	-2.99e-02	6.09e-02	1.22e-02	3.80e-02
7.59e-02	-3.61e-02	5.61e-02	-2.14e-02	8.01e-02	-1.81e-01	1.07e-01	-8.05e-02	6.42e-02
4.57e-02	-7.84e-02	3.39e-02	-1.71e-02	4.87e-02	-7.34e-02	6.12e-02	-3.91e-02	3.81e-02
6.61e-02	-2.08e-02	4.88e-02	-8.61e-02	7.15e-02	1.09e-01	8.41e-02	-6.92e-03	5.44e-02
4.49e-02	6.89e-02	3.35e-02	-4.25e-02	4.85e-02	-2.18e-02	6.06e-02	-3.46e-02	3.78e-02
4.63e-02	3.29e-02	3.44e-02	1.32e-02	4.97e-02	1.54e-02	6.23e-02	1.41e-02	3.88e-02
1.61e-01	1.32e-01	1.16e-01	-3.95e-01	2.15e-01	-8.62e-02	2.32e-01	-2.64e-01	1.58e-01
4.81e-02	-9.36e-02	3.59e-02	-3.53e-02	5.12e-02	2.86e-02	6.35e-02	-1.03e-02	3.98e-02
4.68e-02	-4.19e-02	3.50e-02	2.92e-02	4.99e-02	9.01e-02	6.22e-02	5.33e-02	3.89e-02
6.45e-02	3.71e-03	4.74e-02	-8.72e-02	6.63e-02	-9.92e-02	8.25e-02	-9.15e-02	5.17e-02
4.54e-02	-1.70e-02	3.37e-02	2.51e-02	4.84e-02	-1.22e-02	6.08e-02	1.06e-02	3.79e-02
5.67e-02	5.12e-03	4.19e-02	2.29e-02	6.01e-02	-1.41e-01	7.91e-02	-3.93e-02	4.78e-02
5.18e-02	8.01e-02	3.88e-02	-5.60e-02	5.41e-02	2.51e-03	6.89e-02	-3.30e-02	4.26e-02
5.55e-02	-6.95e-03	4.24e-02	-1.31e-02	6.10e-02	-6.00e-02	7.53e-02	-3.14e-02	4.74e-02
5.69e-02	3.21e-02	4.28e-02	-2.73e-02	6.27e-02	9.16e-03	7.83e-02	-1.38e-02	4.89e-02
8.33e-02	-3.42e-02	6.26e-02	-6.60e-02	9.15e-02	2.22e-02	1.11e-01	-3.12e-02	7.05e-02
8.11e-02	1.30e-02	6.05e-02	-5.85e-02	9.00e-02	-8.32e-02	1.14e-01	-6.81e-02	7.05e-02
4.53e-02	-2.21e-02	3.39e-02	4.11e-02	4.86e-02	-3.16e-02	6.14e-02	1.32e-02	3.81e-02
4.51e-02	-4.13e-03	3.35e-02	7.56e-02	4.83e-02	-9.17e-03	6.06e-02	4.25e-02	3.78e-02
4.65e-02	1.85e-02	3.45e-02	7.96e-02	4.93e-02	3.45e-02	6.23e-02	6.27e-02	3.87e-02
5.48e-02	-6.81e-02	4.04e-02	2.11e-02	5.69e-02	4.35e-02	7.06e-02	2.99e-02	4.43e-02
4.53e-02	2.99e-02	3.38e-02	5.88e-02	4.87e-02	-6.70e-03	6.11e-02	3.36e-02	3.81e-02
5.44e-02	1.61e-02	4.02e-02	-7.95e-02	5.65e-02	1.89e-02	7.27e-02	-4.15e-02	4.46e-02
4.69e-02	-1.13e-01	3.48e-02	4.86e-02	4.92e-02	5.15e-03	6.19e-02	3.20e-02	3.85e-02
4.53e-02	-6.84e-02	3.37e-02	8.82e-02	4.89e-02	-8.83e-02	6.09e-02	1.91e-02	3.81e-02
4.57e-02	4.16e-02	3.40e-02	2.79e-02	4.91e-02	5.97e-02	6.14e-02	4.07e-02	3.83e-02
4.55e-02	3.17e-02	3.39e-02	-3.02e-02	4.88e-02	-5.46e-03	6.12e-02	-2.06e-02	3.81e-02
5.88e-02	7.93e-02	4.48e-02	4.49e-02	6.52e-02	1.37e-01	7.93e-02	8.11e-02	5.03e-02
4.50e-02	-6.62e-04	3.35e-02	-1.14e-01	4.81e-02	3.02e-02	6.05e-02	-5.85e-02	3.76e-02
4.50e-02	2.38e-02	3.36e-02	6.17e-02	4.84e-02	-1.03e-02	6.07e-02	3.37e-02	3.78e-02
4.59e-02	-2.85e-02	3.41e-02	9.35e-03	4.93e-02	-4.45e-02	6.16e-02	-1.10e-02	3.85e-02
4.83e-02	4.52e-03	3.57e-02	-1.43e-01	5.28e-02	-1.09e-01	6.58e-02	-1.30e-01	4.12e-02
6.89e-02	-4.28e-02	5.02e-02	2.11e-02	7.06e-02	6.02e-02	8.72e-02	3.64e-02	5.49e-02
4.87e-02	-1.58e-02	3.64e-02	9.27e-02	5.35e-02	7.28e-03	6.61e-02	5.90e-02	4.16e-02
4.97e-02	6.32e-02	3.69e-02	-4.72e-02	5.44e-02	1.21e-02	6.76e-02	-2.36e-02	4.24e-02
4.58e-02	1.71e-02	3.40e-02	3.52e-02	4.90e-02	-6.57e-03	6.16e-02	1.89e-02	3.84e-02
7.47e-02	4.90e-02	5.46e-02	-6.90e-02	8.22e-02	1.23e-01	9.57e-02	8.69e-03	6.23e-02
4.64e-02	-2.96e-02	3.49e-02	4.42e-02	5.07e-02	-1.27e-02	6.30e-02	2.17e-02	3.95e-02
5.97e-02	-6.59e-02	4.43e-02	1.33e-02	6.21e-02	-1.89e-01	8.33e-02	-6.16e-02	4.97e-02
5.74e-02	4.17e-02	4.31e-02	4.93e-02	6.21e-02	-2.93e-02	7.95e-02	1.89e-02	4.89e-02
4.76e-02	-3.07e-04	3.52e-02	-1.07e-02	5.07e-02	3.84e-02	6.40e-02	8.01e-03	3.97e-02
6.32e-02	2.48e-02	4.73e-02	7.30e-02	6.70e-02	-3.86e-02	8.72e-02	3.07e-02	5.31e-02
5.23e-02	4.53e-02	3.86e-02	8.64e-02	5.51e-02	4.30e-02	6.99e-02	7.00e-02	4.32e-02
4.72e-02	-7.21e-03	3.50e-02	-1.49e-02	5.04e-02	-1.04e-01	6.25e-02	-5.03e-02	3.92e-02

5.15e-02	4.69e-02	3.84e-02	2.66e-02	5.54e-02	2.33e-02	6.96e-02	2.57e-02	4.33e-02
4.60e-02	3.58e-03	3.44e-02	-6.22e-03	4.95e-02	4.44e-02	6.25e-02	1.32e-02	3.88e-02
5.22e-02	2.56e-02	3.86e-02	-5.64e-02	5.46e-02	2.93e-02	6.98e-02	-2.37e-02	4.30e-02
4.58e-02	1.45e-02	3.41e-02	-2.44e-02	4.89e-02	3.38e-02	6.18e-02	-2.13e-03	3.84e-02
4.77e-02	6.15e-02	3.56e-02	5.97e-02	5.12e-02	-5.19e-02	6.31e-02	1.57e-02	3.98e-02
5.90e-02	1.66e-02	4.40e-02	-7.26e-02	6.51e-02	-4.32e-02	8.09e-02	-6.12e-02	5.07e-02
4.50e-02	-2.13e-03	3.35e-02	-5.77e-02	4.83e-02	-1.10e-02	6.06e-02	-3.92e-02	3.78e-02
4.59e-02	1.32e-02	3.42e-02	3.47e-02	4.92e-02	6.16e-02	6.14e-02	4.54e-02	3.84e-02
4.83e-02	-5.37e-02	3.60e-02	-8.26e-02	5.22e-02	-1.26e-01	6.58e-02	-9.91e-02	4.09e-02
4.51e-02	1.27e-02	3.36e-02	5.04e-02	4.84e-02	-4.47e-02	6.08e-02	1.37e-02	3.78e-02
5.90e-02	-4.41e-02	4.41e-02	8.21e-02	6.09e-02	-3.63e-02	7.96e-02	3.75e-02	4.84e-02
5.30e-02	-3.18e-02	3.90e-02	1.45e-01	5.39e-02	-9.12e-02	7.14e-02	5.66e-02	4.29e-02
4.73e-02	-3.05e-02	3.53e-02	2.26e-02	5.05e-02	-6.62e-02	6.41e-02	-1.14e-02	3.97e-02
4.51e-02	5.20e-02	3.36e-02	-1.71e-02	4.84e-02	2.09e-02	6.06e-02	-2.38e-03	3.78e-02
4.53e-02	-1.29e-02	3.37e-02	-2.11e-05	4.84e-02	5.65e-02	6.07e-02	2.21e-02	3.78e-02
5.16e-02	2.32e-02	3.87e-02	1.44e-02	5.57e-02	7.73e-02	6.89e-02	3.92e-02	4.33e-02
6.00e-02	2.89e-02	4.51e-02	-1.46e-02	6.60e-02	-8.95e-02	8.47e-02	-4.36e-02	5.20e-02
1.39e-01	-8.31e-02	1.08e-01	-3.53e-01	1.76e-01	-3.50e-01	2.21e-01	-3.54e-01	1.38e-01
5.62e-02	-3.20e-02	4.15e-02	-6.65e-02	6.02e-02	4.06e-04	7.43e-02	-3.98e-02	4.68e-02
4.72e-02	-2.11e-02	3.53e-02	-5.99e-02	5.11e-02	1.02e-01	6.27e-02	3.66e-03	3.96e-02
1.05e-01	-5.86e-02	8.26e-02	-1.14e-01	1.22e-01	1.70e-01	1.35e-01	4.69e-03	9.04e-02
8.84e-02	6.37e-03	6.46e-02	-2.34e-01	1.03e-01	2.44e-01	1.06e-01	-2.51e-02	7.36e-02
4.60e-02	5.47e-02	3.44e-02	1.84e-03	4.99e-02	2.91e-02	6.24e-02	1.25e-02	3.90e-02
4.80e-02	-7.18e-02	3.56e-02	-2.91e-02	5.16e-02	1.85e-03	6.53e-02	-1.68e-02	4.05e-02
4.75e-02	4.87e-02	3.52e-02	-1.57e-02	5.12e-02	3.88e-02	6.37e-02	5.25e-03	3.99e-02
4.79e-02	-1.84e-02	3.56e-02	3.69e-02	5.09e-02	-2.34e-03	6.41e-02	2.15e-02	3.98e-02
4.52e-02	-5.26e-02	3.36e-02	-3.95e-02	4.85e-02	-9.25e-02	6.06e-02	-5.99e-02	3.79e-02
4.55e-02	6.68e-02	3.40e-02	2.57e-03	4.88e-02	-7.86e-03	6.11e-02	-1.60e-03	3.81e-02
5.72e-02	1.45e-02	4.23e-02	9.72e-02	5.95e-02	2.19e-02	7.62e-02	6.80e-02	4.69e-02
5.13e-02	4.52e-02	3.81e-02	8.73e-02	5.55e-02	6.64e-02	6.91e-02	7.88e-02	4.33e-02
4.69e-02	6.81e-02	3.47e-02	-2.68e-02	4.95e-02	2.45e-02	6.23e-02	-6.53e-03	3.87e-02
4.69e-02	3.92e-02	3.50e-02	-1.03e-02	5.02e-02	-1.04e-01	6.21e-02	-4.69e-02	3.90e-02
5.20e-02	3.92e-02	3.89e-02	2.12e-02	5.57e-02	-1.67e-01	6.69e-02	-5.40e-02	4.28e-02
4.54e-02	2.50e-02	3.37e-02	-5.26e-03	4.84e-02	-9.99e-02	6.05e-02	-4.25e-02	3.78e-02
4.51e-02	2.35e-02	3.36e-02	9.13e-02	4.85e-02	7.36e-02	6.07e-02	8.43e-02	3.79e-02
4.49e-02	6.17e-02	3.35e-02	-3.37e-02	4.83e-02	6.62e-02	6.05e-02	5.25e-03	3.77e-02
4.51e-02	-3.10e-02	3.37e-02	-3.71e-02	4.85e-02	1.08e-01	6.05e-02	1.91e-02	3.78e-02
4.51e-02	-5.40e-02	3.36e-02	-2.45e-02	4.83e-02	4.63e-03	6.05e-02	-1.32e-02	3.78e-02
6.20e-02	3.30e-03	4.58e-02	-3.94e-02	6.49e-02	-8.57e-02	8.04e-02	-5.69e-02	5.05e-02
4.61e-02	7.22e-03	3.45e-02	-7.14e-02	4.94e-02	-3.36e-02	6.20e-02	-5.72e-02	3.86e-02
4.65e-02	-1.97e-02	3.47e-02	7.95e-03	5.02e-02	-9.46e-03	6.28e-02	1.28e-03	3.92e-02
5.36e-02	-1.50e-02	3.99e-02	-4.06e-02	5.78e-02	-1.61e-02	7.20e-02	-3.11e-02	4.51e-02
5.51e-02	-7.02e-02	4.11e-02	-2.67e-02	5.84e-02	-1.33e-01	7.55e-02	-6.73e-02	4.62e-02
4.96e-02	6.40e-03	3.69e-02	5.98e-03	5.31e-02	1.01e-01	6.79e-02	4.24e-02	4.18e-02
4.51e-02	1.69e-02	3.35e-02	-5.63e-03	4.83e-02	3.36e-02	6.05e-02	9.56e-03	3.77e-02
4.53e-02	-1.69e-02	3.37e-02	1.06e-01	4.82e-02	-1.10e-01	6.15e-02	2.27e-02	3.79e-02
1.03e-01	9.33e-04	7.93e-02	3.06e-02	1.13e-01	1.18e-01	1.36e-01	6.45e-02	8.69e-02

6.30e-02	7.19e-02	4.59e-02	-7.25e-02	6.34e-02	1.53e-01	8.54e-02	1.23e-02	5.08e-02
4.58e-02	4.55e-03	3.42e-02	2.21e-02	4.92e-02	4.47e-02	6.16e-02	3.10e-02	3.85e-02
4.49e-02	-3.07e-02	3.35e-02	2.27e-02	4.85e-02	-5.36e-02	6.04e-02	-6.72e-03	3.78e-02
4.90e-02	-3.37e-02	3.65e-02	-1.09e-02	5.29e-02	-2.82e-02	6.61e-02	-1.77e-02	4.13e-02
4.89e-02	6.36e-02	3.65e-02	9.49e-02	5.23e-02	-4.81e-02	6.72e-02	4.08e-02	4.12e-02
5.58e-02	1.48e-02	4.15e-02	4.36e-02	5.93e-02	3.46e-02	7.47e-02	3.99e-02	4.64e-02
7.63e-02	1.03e-01	5.64e-02	8.31e-02	8.18e-02	3.18e-01	9.38e-02	1.79e-01	6.16e-02
5.44e-02	9.84e-03	4.01e-02	-7.43e-03	5.80e-02	3.24e-02	7.21e-02	8.19e-03	4.52e-02
4.53e-02	-3.01e-02	3.37e-02	7.35e-03	4.88e-02	5.84e-02	6.13e-02	2.76e-02	3.82e-02
4.53e-02	5.17e-02	3.37e-02	-1.76e-02	4.86e-02	-1.14e-02	6.12e-02	-1.52e-02	3.81e-02
6.69e-02	-1.17e-02	4.94e-02	-4.09e-02	7.20e-02	-9.57e-02	9.22e-02	-6.19e-02	5.67e-02
6.48e-02	-3.94e-02	4.83e-02	-5.56e-02	7.00e-02	3.65e-02	8.47e-02	-1.85e-02	5.40e-02
4.63e-02	7.50e-03	3.47e-02	-3.10e-02	5.02e-02	3.97e-02	6.24e-02	-3.12e-03	3.91e-02
4.62e-02	-7.69e-03	3.45e-02	-9.79e-02	5.03e-02	6.31e-02	6.20e-02	-3.43e-02	3.90e-02
4.52e-02	4.73e-02	3.36e-02	-1.91e-02	4.84e-02	-5.46e-02	6.06e-02	-3.27e-02	3.78e-02
4.77e-02	-4.36e-02	3.57e-02	-1.28e-02	5.11e-02	-6.60e-02	6.49e-02	-3.32e-02	4.01e-02
5.16e-02	-1.26e-02	3.82e-02	3.77e-02	5.44e-02	1.04e-03	6.88e-02	2.29e-02	4.27e-02
4.62e-02	6.70e-02	3.43e-02	2.70e-02	4.93e-02	1.19e-02	6.16e-02	2.10e-02	3.85e-02
6.21e-02	-2.70e-02	4.60e-02	-1.84e-02	6.61e-02	5.80e-02	8.07e-02	1.20e-02	5.11e-02
8.19e-02	-1.26e-03	5.93e-02	1.62e-01	8.00e-02	2.19e-01	9.81e-02	1.85e-01	6.20e-02
4.55e-02	1.22e-02	3.38e-02	3.77e-02	4.87e-02	-2.92e-02	6.13e-02	1.18e-02	3.81e-02
4.58e-02	-5.58e-02	3.38e-02	2.87e-03	4.85e-02	4.34e-02	6.08e-02	1.87e-02	3.79e-02
5.26e-02	8.92e-03	3.92e-02	1.25e-01	5.49e-02	-2.44e-02	7.15e-02	6.80e-02	4.35e-02
4.58e-02	7.19e-02	3.42e-02	3.58e-02	4.94e-02	7.69e-02	6.16e-02	5.16e-02	3.85e-02
1.14e-01	-8.05e-02	8.11e-02	-2.22e-03	1.13e-01	1.82e-01	1.31e-01	7.16e-02	8.54e-02
1.01e-01	4.18e-02	7.54e-02	4.86e-02	1.09e-01	3.14e-02	1.37e-01	4.24e-02	8.51e-02
4.55e-02	2.03e-02	3.39e-02	-3.66e-02	4.86e-02	-1.25e-01	6.08e-02	-7.11e-02	3.79e-02
4.67e-02	-6.63e-04	3.48e-02	-3.56e-02	5.00e-02	-9.64e-02	6.20e-02	-5.96e-02	3.89e-02
1.67e-01	-2.55e-01	1.25e-01	3.44e-01	1.38e-01	-8.92e-02	2.10e-01	1.96e-01	1.15e-01
6.74e-02	-3.31e-02	4.93e-02	-1.04e-02	7.05e-02	2.91e-02	8.71e-02	4.79e-03	5.48e-02
7.62e-02	1.65e-02	5.82e-02	-1.42e-01	8.94e-02	-1.18e-02	1.06e-01	-8.99e-02	6.84e-02
6.65e-02	-4.33e-02	4.90e-02	8.75e-02	6.73e-02	-1.92e-02	8.79e-02	4.71e-02	5.34e-02
5.74e-02	-5.01e-02	4.33e-02	1.33e-02	6.12e-02	-4.22e-02	7.82e-02	-8.31e-03	4.82e-02
5.31e-02	5.94e-02	3.95e-02	-1.73e-02	5.80e-02	5.71e-03	7.22e-02	-7.85e-03	4.52e-02
4.57e-02	-5.87e-02	3.39e-02	-5.33e-02	4.89e-02	2.64e-02	6.08e-02	-2.18e-02	3.81e-02
4.71e-02	-4.47e-02	3.54e-02	2.29e-02	5.05e-02	2.19e-02	6.33e-02	2.25e-02	3.94e-02
4.93e-02	-4.12e-02	3.68e-02	-9.94e-02	5.23e-02	-4.18e-02	6.64e-02	-7.67e-02	4.11e-02
4.49e-02	-3.97e-02	3.34e-02	2.76e-02	4.82e-02	5.03e-02	6.05e-02	3.63e-02	3.77e-02
4.50e-02	1.03e-02	3.35e-02	5.57e-02	4.83e-02	7.84e-02	6.07e-02	6.44e-02	3.78e-02
4.54e-02	-4.96e-03	3.37e-02	2.90e-02	4.84e-02	-7.09e-02	6.10e-02	-9.35e-03	3.79e-02
4.57e-02	-1.03e-02	3.42e-02	-1.38e-01	5.00e-02	2.16e-03	6.16e-02	-8.30e-02	3.88e-02
4.59e-02	-3.87e-02	3.41e-02	-9.27e-02	4.95e-02	-1.43e-02	6.15e-02	-6.18e-02	3.85e-02
7.04e-02	-4.94e-02	5.32e-02	-6.19e-02	7.61e-02	-1.86e-01	9.15e-02	-1.12e-01	5.85e-02
4.82e-02	-3.11e-02	3.58e-02	3.48e-02	5.11e-02	-4.86e-02	6.48e-02	2.50e-03	4.01e-02
5.87e-02	1.34e-02	4.43e-02	-8.20e-03	6.36e-02	6.65e-02	8.16e-02	2.13e-02	5.02e-02
5.85e-02	2.87e-02	4.36e-02	1.03e-02	6.32e-02	-5.42e-02	8.06e-02	-1.45e-02	4.97e-02
4.58e-02	3.36e-02	3.41e-02	-9.35e-03	4.88e-02	-2.64e-03	6.14e-02	-6.87e-03	3.82e-02

4.53e-02	-6.29e-03	3.39e-02	-1.03e-03	4.90e-02	-5.69e-02	6.15e-02	-2.29e-02	3.83e-02
4.60e-02	2.06e-02	3.43e-02	3.94e-02	4.93e-02	-5.79e-02	6.24e-02	2.11e-03	3.87e-02
4.49e-02	-2.21e-02	3.34e-02	2.75e-02	4.81e-02	-7.55e-02	6.05e-02	-1.26e-02	3.76e-02
4.88e-02	-4.23e-02	3.65e-02	-6.72e-02	5.29e-02	-9.78e-02	6.67e-02	-7.95e-02	4.15e-02
4.53e-02	-2.26e-02	3.39e-02	-2.92e-02	4.89e-02	-9.78e-02	6.08e-02	-5.60e-02	3.81e-02
4.54e-02	2.34e-02	3.38e-02	-1.17e-02	4.88e-02	2.62e-02	6.11e-02	2.89e-03	3.81e-02
4.58e-02	1.89e-02	3.42e-02	1.11e-02	4.94e-02	-1.17e-01	6.30e-02	-3.78e-02	3.88e-02
5.62e-02	-2.95e-02	4.13e-02	8.16e-02	5.79e-02	7.08e-03	7.36e-02	5.30e-02	4.55e-02
8.27e-02	4.87e-03	6.31e-02	4.45e-02	8.96e-02	-1.69e-01	1.23e-01	-3.48e-02	7.24e-02
7.42e-02	-2.34e-02	5.35e-02	-2.31e-02	7.67e-02	7.23e-02	9.32e-02	1.45e-02	5.92e-02
7.03e-02	4.71e-02	5.30e-02	-2.67e-02	7.86e-02	-2.01e-03	9.74e-02	-1.71e-02	6.12e-02
4.57e-02	-4.21e-03	3.40e-02	3.72e-04	4.90e-02	-7.34e-02	6.16e-02	-2.84e-02	3.83e-02
4.52e-02	1.10e-02	3.37e-02	2.79e-02	4.86e-02	1.79e-02	6.10e-02	2.38e-02	3.80e-02
4.60e-02	-4.79e-02	3.43e-02	-4.84e-02	4.95e-02	-6.57e-02	6.18e-02	-5.49e-02	3.86e-02
6.54e-02	1.52e-02	4.95e-02	-3.79e-02	7.27e-02	1.20e-01	8.60e-02	2.58e-02	5.55e-02
6.09e-02	-6.82e-02	4.63e-02	8.12e-03	6.51e-02	6.28e-02	8.01e-02	2.96e-02	5.05e-02
4.83e-02	-7.59e-02	3.60e-02	4.18e-02	5.08e-02	1.18e-01	6.31e-02	7.13e-02	3.96e-02
6.62e-02	-1.40e-01	4.92e-02	-6.50e-02	6.90e-02	-1.03e-01	8.75e-02	-7.97e-02	5.42e-02
6.56e-02	2.39e-02	4.82e-02	-6.69e-02	7.15e-02	-7.94e-02	9.03e-02	-7.12e-02	5.61e-02
5.46e-02	1.15e-02	4.04e-02	-9.61e-02	6.01e-02	-4.09e-02	7.40e-02	-7.41e-02	4.66e-02
6.11e-02	-3.22e-02	4.61e-02	-4.09e-02	6.66e-02	-1.94e-01	8.80e-02	-9.84e-02	5.31e-02
7.04e-02	1.00e-02	5.23e-02	-4.71e-02	7.69e-02	-9.62e-02	9.82e-02	-6.63e-02	6.06e-02
5.54e-02	3.82e-03	4.14e-02	-2.23e-02	5.92e-02	2.63e-02	7.51e-02	-3.04e-03	4.65e-02
4.49e-02	4.17e-03	3.35e-02	8.33e-02	4.83e-02	-7.33e-02	6.04e-02	2.22e-02	3.77e-02
4.54e-02	1.17e-03	3.38e-02	2.78e-02	4.87e-02	2.49e-02	6.10e-02	2.65e-02	3.81e-02
4.53e-02	5.47e-04	3.38e-02	-5.36e-03	4.87e-02	2.15e-02	6.08e-02	5.05e-03	3.80e-02
8.66e-02	1.25e-02	6.40e-02	-1.13e-01	9.72e-02	8.50e-02	1.12e-01	-3.20e-02	7.34e-02
4.89e-02	2.11e-02	3.63e-02	8.33e-02	5.17e-02	9.30e-03	6.58e-02	5.50e-02	4.07e-02
5.46e-02	2.97e-02	3.99e-02	-2.24e-02	5.82e-02	3.81e-02	7.19e-02	1.38e-03	4.52e-02
1.13e-01	-4.34e-02	8.37e-02	-1.15e-01	1.25e-01	-1.69e-01	1.59e-01	-1.37e-01	9.82e-02
4.64e-02	-5.81e-02	3.47e-02	-1.19e-01	5.05e-02	1.72e-02	6.21e-02	-6.57e-02	3.91e-02
4.72e-02	-1.52e-02	3.51e-02	5.11e-02	5.00e-02	5.31e-03	6.32e-02	3.38e-02	3.92e-02
5.75e-02	7.61e-03	4.24e-02	6.83e-02	6.01e-02	-8.53e-03	7.72e-02	3.86e-02	4.74e-02
4.75e-02	-4.14e-02	3.55e-02	-5.54e-02	5.11e-02	2.87e-03	6.47e-02	-3.31e-02	4.01e-02
5.49e-02	-1.74e-02	4.07e-02	3.54e-02	5.81e-02	-1.06e-01	7.51e-02	-1.87e-02	4.59e-02
6.20e-02	-4.72e-03	4.64e-02	-3.18e-02	6.74e-02	1.38e-01	7.98e-02	3.67e-02	5.15e-02
4.52e-02	3.51e-03	3.36e-02	4.84e-02	4.83e-02	-9.30e-03	6.07e-02	2.64e-02	3.78e-02
6.87e-02	-4.89e-02	5.10e-02	-2.82e-02	7.25e-02	1.38e-01	8.60e-02	3.89e-02	5.54e-02
4.49e-02	-1.45e-02	3.35e-02	1.68e-03	4.83e-02	3.35e-02	6.05e-02	1.41e-02	3.77e-02
4.74e-02	-1.20e-01	3.57e-02	-3.52e-02	5.08e-02	-8.84e-03	6.34e-02	-2.48e-02	3.96e-02
5.22e-02	-1.05e-02	3.88e-02	-7.61e-02	5.67e-02	6.35e-02	6.89e-02	-2.13e-02	4.38e-02
5.86e-02	4.48e-02	4.35e-02	-4.22e-02	6.42e-02	-1.19e-01	8.29e-02	-7.11e-02	5.08e-02
4.54e-02	-4.89e-02	3.38e-02	-6.73e-02	4.88e-02	-1.25e-01	6.14e-02	-8.91e-02	3.82e-02
5.36e-02	-5.27e-02	3.99e-02	4.24e-02	5.91e-02	7.72e-03	7.33e-02	2.88e-02	4.60e-02
4.82e-02	6.34e-02	3.59e-02	-7.82e-02	5.31e-02	1.83e-02	6.55e-02	-4.00e-02	4.12e-02
4.55e-02	-3.09e-02	3.39e-02	2.36e-02	4.87e-02	3.69e-02	6.10e-02	2.89e-02	3.80e-02
4.58e-02	1.49e-02	3.41e-02	-6.03e-02	4.89e-02	-8.95e-02	6.12e-02	-7.18e-02	3.82e-02

5.01e-02	8.92e-03	3.74e-02	-6.71e-03	5.38e-02	-6.81e-02	6.64e-02	-3.11e-02	4.18e-02
4.67e-02	8.80e-04	3.49e-02	9.82e-03	5.03e-02	-1.20e-02	6.31e-02	1.09e-03	3.93e-02
4.93e-02	-1.49e-02	3.65e-02	-2.74e-02	5.23e-02	8.31e-02	6.73e-02	1.46e-02	4.13e-02
4.85e-02	-4.72e-02	3.61e-02	-1.21e-02	5.17e-02	-6.78e-02	6.55e-02	-3.29e-02	4.06e-02
4.79e-02	-3.28e-02	3.59e-02	-2.17e-02	5.16e-02	5.80e-03	6.44e-02	-1.09e-02	4.03e-02
5.10e-02	9.22e-04	3.77e-02	3.01e-02	5.39e-02	1.50e-03	6.82e-02	1.93e-02	4.23e-02
5.19e-02	4.65e-02	3.89e-02	2.64e-02	5.57e-02	7.11e-03	6.97e-02	1.89e-02	4.35e-02
4.54e-02	-7.34e-02	3.38e-02	-1.20e-02	4.88e-02	-3.39e-03	6.13e-02	-8.60e-03	3.82e-02
5.17e-02	4.02e-02	3.84e-02	4.11e-02	5.54e-02	2.84e-02	6.95e-02	3.55e-02	4.33e-02
4.93e-02	-3.29e-02	3.71e-02	-3.95e-02	5.34e-02	-4.67e-02	6.68e-02	-4.21e-02	4.17e-02
4.56e-02	3.04e-02	3.41e-02	-3.21e-02	4.94e-02	-1.74e-03	6.18e-02	-2.05e-02	3.86e-02
4.95e-02	-4.47e-02	3.72e-02	5.86e-02	5.25e-02	-8.66e-03	6.68e-02	3.26e-02	4.13e-02
4.59e-02	3.37e-02	3.38e-02	-6.50e-02	4.84e-02	1.39e-01	6.17e-02	1.35e-02	3.80e-02
4.53e-02	5.26e-02	3.37e-02	6.57e-03	4.85e-02	-7.59e-02	6.05e-02	-2.51e-02	3.78e-02
5.07e-02	1.30e-02	3.77e-02	7.14e-02	5.35e-02	3.52e-02	6.77e-02	5.73e-02	4.20e-02
4.85e-02	-2.51e-04	3.61e-02	2.92e-02	5.23e-02	-7.88e-02	6.43e-02	-1.38e-02	4.06e-02
4.86e-02	6.27e-03	3.59e-02	6.83e-02	5.23e-02	6.29e-02	6.55e-02	6.64e-02	4.08e-02
4.61e-02	-1.08e-02	3.45e-02	-2.16e-02	4.96e-02	-2.85e-02	6.23e-02	-2.47e-02	3.88e-02
4.52e-02	-2.17e-02	3.36e-02	3.23e-02	4.84e-02	-1.72e-02	6.08e-02	1.30e-02	3.79e-02
4.49e-02	3.48e-03	3.34e-02	9.35e-03	4.82e-02	-1.09e-01	6.03e-02	-3.58e-02	3.76e-02
4.86e-02	-3.14e-02	3.63e-02	-7.31e-02	5.19e-02	-2.43e-02	6.56e-02	-5.41e-02	4.07e-02
8.58e-02	-7.02e-02	6.45e-02	-1.72e-01	9.67e-02	8.64e-03	1.13e-01	-9.88e-02	7.34e-02
5.10e-02	-2.26e-02	3.75e-02	8.52e-02	5.54e-02	4.76e-02	6.87e-02	6.99e-02	4.31e-02
4.52e-02	2.19e-03	3.37e-02	-8.76e-03	4.87e-02	-1.22e-01	6.06e-02	-5.30e-02	3.79e-02
4.64e-02	-5.54e-02	3.45e-02	-8.54e-02	4.99e-02	-3.79e-02	6.22e-02	-6.66e-02	3.89e-02
4.82e-02	5.72e-02	3.60e-02	2.36e-02	5.16e-02	-8.38e-02	6.37e-02	-1.80e-02	4.01e-02
2.20e-01	2.41e-02	1.68e-01	2.64e-01	2.20e-01	5.26e-01	2.37e-01	3.77e-01	1.61e-01
4.49e-02	-2.92e-02	3.34e-02	-1.55e-03	4.82e-02	-1.04e-01	6.04e-02	-4.15e-02	3.76e-02
4.94e-02	8.54e-02	3.67e-02	4.07e-02	5.35e-02	7.17e-02	6.65e-02	5.30e-02	4.16e-02
4.58e-02	-1.06e-02	3.41e-02	9.39e-02	4.87e-02	1.08e-02	6.15e-02	6.19e-02	3.81e-02
5.46e-02	2.65e-02	4.05e-02	3.99e-02	5.84e-02	-3.13e-02	7.22e-02	1.17e-02	4.54e-02
4.54e-02	-1.40e-02	3.38e-02	-6.36e-02	4.86e-02	-8.84e-02	6.08e-02	-7.31e-02	3.80e-02

Table S4. Selection process and F-statistics of instrumental variables used in Mendelian randomisation analyses

Exposure	Number of SNPs identified by GWAS	Number of SNPs with p-value < 5×10^{-8}	Number of SNPs after clumping to r^2 0.001	Number of SNPs INFO score > 0.9	Number of SNPs in HWE	F statistic
Ever smoking	13,595,219	23,316	248	246	246	10.9 (females)<br>9.5 (males)<br>18.7 (overall)
Smoking continuation	13,642,427	2,267	22	21	21	7.1 (females)<br>10.2 (males)<br>16.5 (overall)
Cigarettes per day	13,763,313	4,687	51	48	48	15.7 (females)<br>15.6 (males)<br>27.4 (overall)

Abbreviations: GWAS, genome-wide association study; HWE, Hardy-Weinberg equilibrium; SNP, single nucleotide polymorphism. For more details on the SNPs see **Supplementary Table S3** and the GWAS by the GSCAN consortium(15). For both ever smoking and smoking continuation, the F-statistic is derived by comparing a full model that includes the predictors to a null model that contains only the intercept. In the case of cigarettes per day, the F-statistic is calculated from a linear regression model. For more details, see **Supplemental Methods**.

Table S5. Characteristics of study population in Mendelian randomisation analysis

Characteristics	No. of non-missing values in females	Females (n = 181,183)	No. of non-missing values in males	Males (n = 156,203)
Age, years	181,183	56.7 (7.9)	156,203	57.1 (8.1)
Ethnicity	181,183		156,203	
White		181,183 (100.0)		156,203 (100.0)
Other*		0 (0.0)		0 (0.0)
Smoking status	180,564		155,647	
Never		107,384 (59.3)		76,390 (48.9)
Former		57,607 (31.8)		60,864 (39.0)
Current		15,573 (8.6)		18,393 (11.8)
Number of cigarettes smoked daily in current smokers	11,921	15 [10.0, 20.0]	11,314	15 [10.0, 20.0]
Cigarettes smoked per day in current or former smokers	48,647		51,804	
1-5		4,325 (2.4)		2,309 (1.5)
6-15		22,736 (12.5)		18,422 (11.8)
16-25		17,704 (9.8)		22,825 (14.6)
26-35		2,581 (1.4)		5,496 (3.5)
≥36		1,313 (0.7)		5,071 (3.2)
Socioeconomic status	180,971		156,016	
Townsend deprivation index score		-2.36 [-3.7, 0.0]		-2.36 [-3.8, 0.1]
Townsend deprivation thirds				
Low (≥1.40)		29,454 (16.3)		27,015 (17.3)
Middle (≥–2.08 - <1.40)		53,559 (29.6)		44,810 (28.7)
High (<–2.08)		97,958 (54.1)		84,191 (53.9)
Outcomes (first occurrence either before or after study entry)				
Cardiovascular disease		19,927 (11.0)		34,610 (22.2)
Coronary heart disease		15,445 (8.5)		29,375 (18.8)
Myocardial infarction		4,295 (2.4)		12,653 (8.1)
Stroke		5,952 (3.3)		8,283 (5.3)
Ischaemic stroke		4,808 (2.7)		7,352 (4.7)
Intracerebral haemorrhage		802 (0.4)		1,003 (0.6)
Subarachnoid haemorrhage		868 (0.5)		551 (0.4)

Numbers are presented as mean (standard deviation), median [25th, 75th percentile], or number (percentage). *includes Asian or Asian British, Indian, Pakistani, Bangladeshi, any other Asian background, Chinese, black or black British, Caribbean, African, any other black background, other ethnic group, white and black Caribbean, white and black African, white and Asian, and any other mixed background.

Table S6. Results of the Q test for heterogeneity

Outcome / Exposure	Sex	Q test	
		Q	P value
**Ever smoking**			
Cardiovascular disease	Females	305.6	0.005
	Males	391.3	<0.001
	Overall	458.2	<0.001
Coronary heart disease	Females	290.9	0.023
	Males	395.3	<0.001
	Overall	458.6	<0.001
Myocardial infarction	Females	290.2	0.025
	Males	309.6	0.003
	Overall	347.7	<0.001
Stroke	Females	302.7	0.007
	Males	262.8	0.208
	Overall	315.3	0.002
Ischaemic stroke	Females	313.8	0.002
	Males	270.5	0.127
	Overall	314.6	0.002
Intracerebral haemorrhage	Females	264.7	0.185
	Males	255.2	0.313
	Overall	262.8	0.208
Subarachnoid haemorrhage	Females	254.4	0.326
	Males	260.9	0.232
	Overall	261.1	0.228
**Smoking continuation**			
Cardiovascular disease	Females	42.8	0.002
	Males	49.9	<0.001
	Overall	62.4	<0.001
Coronary heart disease	Females	36.9	0.012
	Males	45.8	<0.001
	Overall	56.6	<0.001
Myocardial infarction	Females	28.1	0.106
	Males	39.7	0.005
	Overall	46.1	<0.001
Stroke	Females	24.0	0.244
	Males	36.6	0.013
	Overall	31.2	0.052
Ischaemic stroke	Females	17.2	0.643
	Males	38.2	0.008
	Overall	27.1	0.133
Intracerebral haemorrhage	Females	23.3	0.272
	Males	24.4	0.224
	Overall	29.1	0.085
Subarachnoid haemorrhage	Females	26.4	0.153
	Males	19.3	0.499
	Overall	29.4	0.080
**Cigarettes per day**			
Cardiovascular disease	Females	92.3	<0.001
	Males	161.7	<0.001
	Overall	210.1	<0.001
Coronary heart disease	Females	87.7	<0.001

Outcome / Exposure	Sex	Q test	
		Q	P value
	Males	182.7	<0.001
	Overall	221.9	<0.001
Myocardial infarction	Females	78.4	0.003
	Males	118.9	<0.001
	Overall	141.9	<0.001
Stroke	Females	52.2	0.277
	Males	62.3	0.067
	Overall	71.5	0.012
Ischaemic stroke	Females	55.2	0.192
	Males	55.6	0.182
	Overall	67.7	0.025
Intracerebral haemorrhage	Females	34.0	0.923
	Males	61.6	0.075
	Overall	41.7	0.690
Subarachnoid haemorrhage	Females	46.9	0.477
	Males	51.0	0.319
	Overall	62.2	0.068

Table S7. MR-PRESSO

		Primary analysis (IVW)	MR-PRESSO			
**Outcome / Exposure**	**Sex**	**OR (95%CI)**	**Global p-value**	**Outliers detected**	**OR (95%CI)**	**p-value distortion test***
**Ever smoking**						
Cardiovascular disease	Females	1.81 (1.57, 2.07)	0.008	–	–	–
	Males	1.66 (1.46, 1.89)	<0.001	✓	1.62 (1.43, 1.84)	0.713
	Overall	1.72 (1.54, 1.91)	<0.001	✓	1.70 (1.53, 1.89)	0.851
Coronary heart disease	Females	1.86 (1.60, 2.17)	0.029	–	–	–
	Males	1.68 (1.46, 1.92)	<0.001	–	–	–
	Overall	1.75 (1.55, 1.96)	<0.001	✓	1.75 (1.57, 1.96)	0.938
Myocardial infarction	Females	2.08 (1.58, 2.73)	0.028	–	–	–
	Males	1.73 (1.46, 2.05)	0.004	–	–	–
	Overall	1.82 (1.56, 2.12)	<0.001	✓	1.79 (1.54, 2.09)	0.864
Stroke	Females	1.62 (1.27, 2.05)	0.008	✓	1.58 (1.25, 1.99)	0.839
	Males	1.41 (1.16, 1.70)	0.210	–	–	–
	Overall	1.49 (1.27, 1.75)	0.001	✓	1.47 (1.26, 1.72)	0.842
Ischaemic stroke	Females	1.51 (1.15, 1.97)	0.002	–	–	–
	Males	1.48 (1.20, 1.81)	0.125	–	–	–
	Overall	1.49 (1.26, 1.77)	0.002	✓	1.47 (1.24, 1.73)	0.843
Intracerebral haemorrhage	Females	1.24 (0.68, 2.24)	0.181	–	–	–
	Males	0.96 (0.57, 1.62)	0.315	–	–	–
	Overall	1.08 (0.73, 1.60)	0.204	–	–	–
Subarachnoid haemorrhage	Females	2.49 (1.43, 4.36)	0.329	–	–	–
	Males	0.96 (0.47, 1.94)	0.229	–	–	–
	Overall	1.71 (1.10, 2.66)	0.228	–	–	–
**Smoking continuation**						
Cardiovascular disease	Females	1.34 (0.91, 1.97)	0.002	–	–	–
	Males	1.27 (0.90, 1.79)	<0.001	✓	1.19 (0.87, 1.63)	0.511
	Overall	1.30 (0.96, 1.75)	<0.001	✓	1.24 (0.93, 1.65)	0.700
Coronary heart disease	Females	1.49 (1.00, 2.23)	0.014	–	–	–
	Males	1.26 (0.89, 1.78)	<0.001	✓	1.17 (0.86, 1.60)	0.475
	Overall	1.34 (0.99, 1.82)	<0.001	✓	1.44 (1.09, 1.92)	0.600
Myocardial infarction	Females	2.04 (1.09, 3.85)	0.121	–	–	–
	Males	1.17 (0.74, 1.85)	0.007	✓	1.07 (0.71, 1.63)	0.336
	Overall	1.36 (0.89, 2.07)	<0.001	✓	1.19 (0.80, 1.77)	0.188
Stroke	Females	1.00 (0.60, 1.64)	0.219	–	–	–
	Males	1.13 (0.66, 1.92)	0.017	–	–	–
	Overall	1.07 (0.74, 1.55)	0.049	–	–	–
Ischaemic stroke	Females	1.05 (0.63, 1.74)	0.640	–	–	–
	Males	1.17 (0.66, 2.08)	0.009	–	–	–
	Overall	1.12 (0.77, 1.63)	0.125	–	–	–
Intracerebral haemorrhage	Females	0.71 (0.19, 2.67)	0.285	–	–	–
	Males	0.80 (0.24, 2.69)	0.255	–	–	–
	Overall	0.76 (0.28, 2.04)	0.096	–	–	–
Subarachnoid haemorrhage	Females	1.50 (0.40, 5.70)	0.128	–	–	–
	Males	1.41 (0.33, 6.07)	0.485	–	–	–
	Overall	1.46 (0.49, 4.39)	0.062	–	–	–
**Cigarettes per day**						
Cardiovascular disease	Females	1.44 (1.19, 1.73)	0.000	✓	1.72 (1.39, 2.12)	0.102
	Males	1.29 (1.06, 1.59)	<0.001	✓	1.55 (1.28, 1.87)	0.124
	Overall	1.35 (1.13, 1.62)	<0.001	✓	1.61 (1.38, 1.88)	0.069
Coronary heart disease	Females	1.61 (1.31, 1.97)	<0.001	✓	1.89 (1.49, 2.40)	0.130

		Primary analysis (IVW)	MR-PRESSO			
**Outcome / Exposure**	**Sex**	**OR (95%CI)**	**Global p-value**	**Outliers detected**	**OR (95%CI)**	**p-value distortion test***
	Males	1.31 (1.04, 1.65)	<0.001	✓	1.58 (1.28, 1.96)	0.134
	Overall	1.42 (1.16, 1.73)	<0.001	✓	1.69 (1.41, 2.02)	0.073
Myocardial infarction	Females	1.66 (1.18, 2.35)	0.003	✓	1.58 (1.17, 2.13)	0.705
	Males	1.33 (1.03, 1.72)	<0.001	✓	1.64 (1.27, 2.11)	0.219
	Overall	1.42 (1.11, 1.80)	<0.001	✓	1.66 (1.32, 2.09)	0.244
Stroke	Females	1.12 (0.88, 1.43)	0.200	–	–	–
	Males	1.22 (0.97, 1.54)	0.063	–	–	–
	Overall	1.18 (0.98, 1.42)	0.008	✓	1.37 (1.10, 1.70)	0.314
Ischaemic stroke	Females	1.16 (0.88, 1.54)	0.170	–	–	–
	Males	1.29 (1.03, 1.62)	0.163	–	–	–
	Overall	1.24 (1.02, 1.51)	0.019	–	–	–
Intracerebral haemorrhage	Females	0.96 (0.51, 1.77)	0.900	–	–	–
	Males	0.78 (0.42, 1.47)	0.081	–	–	–
	Overall	0.85 (0.56, 1.29)	0.635	–	–	–
Subarachnoid haemorrhage	Females	1.11 (0.61, 2.00)	0.499	–	–	–
	Males	1.70 (0.79, 3.65)	0.301	–	–	–
	Overall	1.31 (0.77, 2.22)	0.076	–	–	–

*The distortion test tests the difference between the estimate before and after removal of detected outlier SNPs. Abbreviations: CI, confidence interval; IVW, inverse-variance weighting; MR-PRESSO, MR Pleiotropy Residual Sum and Outlier; OR, odds ratio; SNP, single nucleotide polymorphism.

Supplementary Figures

Figure S1. Multivariable adjusted Cox regression of the association between ever smoking and risk of cardiovascular disease outcomes in females, males, and the overall population.

Outcome	No. of events	HR (95% CI)	RHR (95% CI)
**Cardiovascular disease**			
Females	19,076	1.40 (1.36, 1.44)	1.14 (1.09, 1.18)
Males	28,768	1.23 (1.20, 1.26)	
Overall	47,844	1.29 (1.27, 1.32)	
**Coronary heart disease**			
Females	14,450	1.42 (1.37, 1.47)	1.15 (1.10, 1.20)
Males	23,458	1.24 (1.20, 1.27)	
Overall	37,908	1.30 (1.27, 1.33)	
**Myocardial infarction**			
Females	3,192	1.68 (1.56, 1.80)	1.31 (1.20, 1.42)
Males	6,820	1.29 (1.22, 1.35)	
Overall	10,012	1.39 (1.34, 1.45)	
**Stroke**			
Females	4,626	1.34 (1.26, 1.43)	1.10 (1.01, 1.20)
Males	5,310	1.22 (1.15, 1.29)	
Overall	9,936	1.27 (1.22, 1.33)	
**Ischaemic stroke**			
Females	3,392	1.34 (1.25, 1.44)	1.12 (1.02, 1.23)
Males	4,388	1.20 (1.13, 1.27)	
Overall	7,780	1.25 (1.20, 1.31)	
**Intracerebral haemorrhage**			
Females	709	1.25 (1.07, 1.47)	1.04 (0.83, 1.30)
Males	664	1.20 (1.03, 1.41)	
Overall	1,373	1.23 (1.10, 1.37)	
**Subarachnoid haemorrhage**			
Females	525	1.49 (1.24, 1.78)	0.90 (0.66, 1.22)
Males	258	1.66 (1.29, 2.13)	
Overall	783	1.54 (1.33, 1.79)	

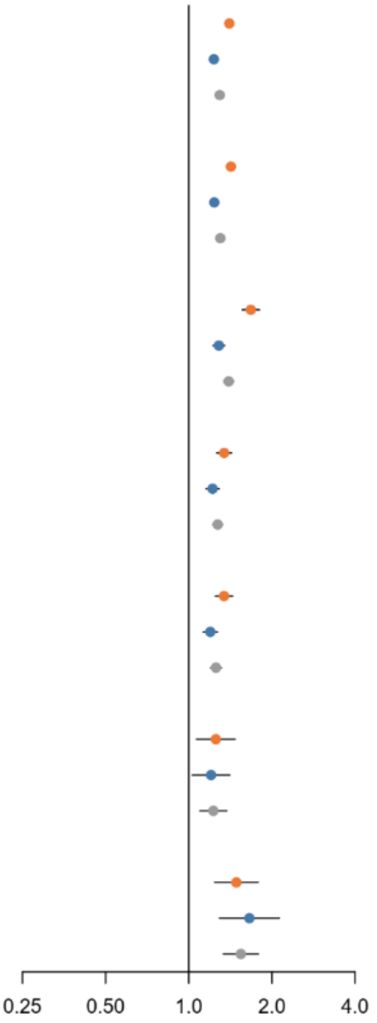

Cox regressions were performed in 468,838 UK Biobank participants and were adjusted for sex, Townsend deprivation index (an area-based measure of socioeconomic status), systolic blood pressure, antihypertensive medication, total cholesterol, high-density lipoprotein cholesterol, C-reactive protein (log-transformed), type 2 diabetes mellitus, and body mass index, as well as interaction terms between sex and these factors. Abbreviations: CI, confidence interval; HR, hazard ratio; RHR, ratio of hazard ratios.

Figure S2. Multivariable adjusted Cox regression of the association between smoking continuation and risk of cardiovascular disease outcomes in females, males, and the overall population.

Outcome	No. of events	HR (95% CI)	RHR (95% CI)
**Cardiovascular disease**			
Females	19,076	1.64 (1.55, 1.73)	1.13 (1.06, 1.21)
Males	28,768	1.45 (1.39, 1.51)	
Overall	47,844	1.51 (1.46, 1.56)	
**Coronary heart disease**			
Females	14,450	1.58 (1.49, 1.68)	1.14 (1.06, 1.23)
Males	23,458	1.39 (1.32, 1.45)	
Overall	37,908	1.45 (1.40, 1.51)	
**Myocardial infarction**			
Females	3,192	2.22 (1.97, 2.51)	1.31 (1.13, 1.52)
Males	6,820	1.69 (1.56, 1.84)	
Overall	10,012	1.84 (1.72, 1.97)	
**Stroke**			
Females	4,626	1.83 (1.64, 2.05)	1.06 (0.92, 1.23)
Males	5,310	1.72 (1.57, 1.89)	
Overall	9,936	1.77 (1.64, 1.90)	
**Ischaemic stroke**			
Females	3,392	1.69 (1.48, 1.93)	0.94 (0.80, 1.12)
Males	4,388	1.79 (1.61, 1.98)	
Overall	7,780	1.75 (1.61, 1.90)	
**Intracerebral haemorrhage**			
Females	709	2.04 (1.52, 2.74)	1.49 (0.99, 2.24)
Males	664	1.37 (1.03, 1.83)	
Overall	1,373	1.65 (1.34, 2.03)	
**Subarachnoid haemorrhage**			
Females	525	2.42 (1.77, 3.29)	1.43 (0.86, 2.37)
Males	258	1.69 (1.13, 2.54)	
Overall	783	2.11 (1.65, 2.70)	

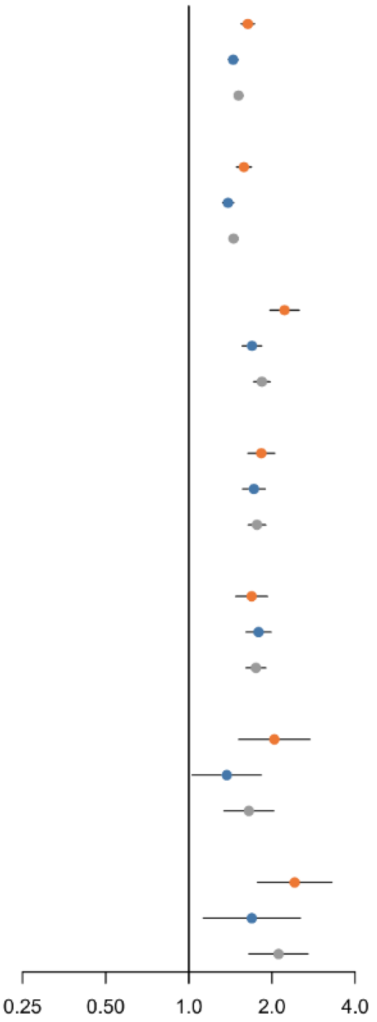

Cox regressions were performed in 468,838 UK Biobank participants and were adjusted for sex, Townsend deprivation index (an area-based measure of socioeconomic status), systolic blood pressure, antihypertensive medication, total cholesterol, high-density lipoprotein cholesterol, C-reactive protein (log-transformed), type 2 diabetes mellitus, and body mass index, as well as interaction terms between sex and these factors. Abbreviations: CI, confidence interval; HR, hazard ratio; RHR, ratio of hazard ratios.

Figure S3. Multivariable adjusted Cox regression of the association between the number of cigarettes smoked per day and risk of cardiovascular disease outcomes in females, males, and the overall population.

Outcome	No. of events	HR (95% CI)	RHR (95% CI)
**Cardiovascular disease**			
Females	19,076	1.11 (1.08, 1.14)	1.04 (1.01, 1.08)
Males	28,768	1.07 (1.05, 1.09)	
Overall	47,844	1.08 (1.06, 1.10)	
**Coronary heart disease**			
Females	14,450	1.12 (1.09, 1.16)	1.06 (1.02, 1.10)
Males	23,458	1.06 (1.04, 1.08)	
Overall	37,908	1.08 (1.06, 1.10)	
**Myocardial infarction**			
Females	3,192	1.11 (1.04, 1.18)	1.09 (1.01, 1.18)
Males	6,820	1.02 (0.98, 1.06)	
Overall	10,012	1.04 (1.00, 1.07)	
**Stroke**			
Females	4,626	1.07 (1.01, 1.14)	0.98 (0.91, 1.05)
Males	5,310	1.10 (1.05, 1.15)	
Overall	9,936	1.09 (1.05, 1.13)	
**Ischaemic stroke**			
Females	3,392	1.10 (1.02, 1.17)	0.99 (0.92, 1.08)
Males	4,388	1.10 (1.05, 1.15)	
Overall	7,780	1.10 (1.06, 1.14)	
**Intracerebral haemorrhage**			
Females	709	1.02 (0.87, 1.20)	0.94 (0.77, 1.15)
Males	664	1.08 (0.96, 1.22)	
Overall	1,373	1.06 (0.96, 1.17)	
**Subarachnoid haemorrhage**			
Females	525	0.99 (0.83, 1.19)	0.91 (0.70, 1.18)
Males	258	1.09 (0.90, 1.32)	
Overall	783	1.04 (0.91, 1.18)	

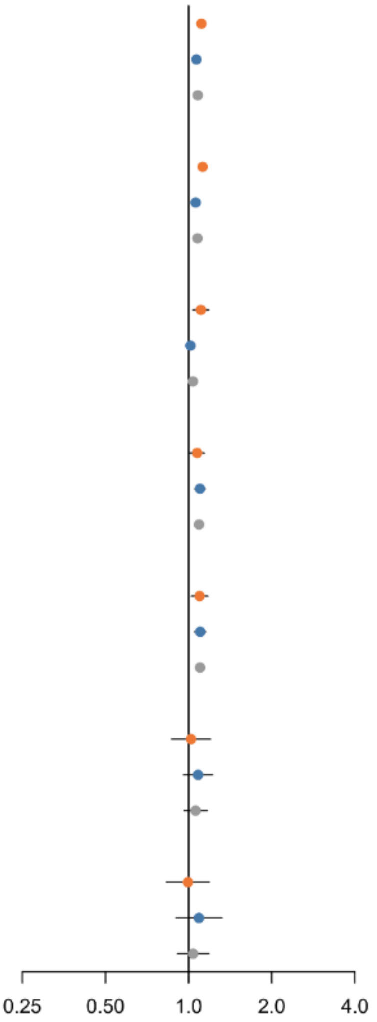

The variable representing number of cigarettes smoked per day was categorized into the following categories: 1=1-5, 2=6-15, 3=16-25, 4=26-35, 5=36+ and was analysed as a continuous variable. Cox regressions were performed in 468,838 UK Biobank participants and were adjusted for sex, Townsend deprivation index (an area-based measure of socioeconomic status), systolic blood pressure, antihypertensive medication, total cholesterol, high-density lipoprotein cholesterol, C-reactive protein (log-transformed), type 2 diabetes mellitus, and body mass index, as well as interaction terms between sex and these factors. Abbreviations: CI, confidence interval; HR, hazard ratio; RHR, ratio of hazard ratios.

Figure S4. Cox regression of the association between ever smoking and risk of cardiovascular disease outcomes in females, males, and the overall population, using the same UK Biobank participants as included in MR analyses.

Outcome	No. of events	HR (95% CI)	RHR (95% CI)
**Cardiovascular disease**			
Females	12,641	1.48 (1.42, 1.53)	1.10 (1.05, 1.15)
Males	19,579	1.34 (1.31, 1.38)	
Overall	32,220	1.39 (1.36, 1.42)	
**Coronary heart disease**			
Females	9,528	1.51 (1.45, 1.57)	1.12 (1.06, 1.18)
Males	15,889	1.35 (1.31, 1.40)	
Overall	25,417	1.41 (1.37, 1.44)	
**Myocardial infarction**			
Females	2,143	1.76 (1.62, 1.93)	1.30 (1.17, 1.44)
Males	4,645	1.36 (1.28, 1.44)	
Overall	6,788	1.47 (1.40, 1.54)	
**Stroke**			
Females	3,113	1.38 (1.28, 1.49)	1.05 (0.95, 1.16)
Males	3,690	1.31 (1.23, 1.40)	
Overall	6,803	1.34 (1.28, 1.41)	
**Ischaemic stroke**			
Females	2,282	1.40 (1.29, 1.53)	1.08 (0.97, 1.21)
Males	3,055	1.30 (1.21, 1.39)	
Overall	5,337	1.34 (1.27, 1.41)	
**Intracerebral haemorrhage**			
Females	479	1.21 (0.99, 1.47)	0.91 (0.70, 1.20)
Males	467	1.32 (1.10, 1.58)	
Overall	946	1.26 (1.11, 1.45)	
**Subarachnoid haemorrhage**			
Females	352	1.49 (1.19, 1.86)	0.93 (0.64, 1.35)
Males	168	1.60 (1.18, 2.17)	
Overall	520	1.53 (1.28, 1.82)	

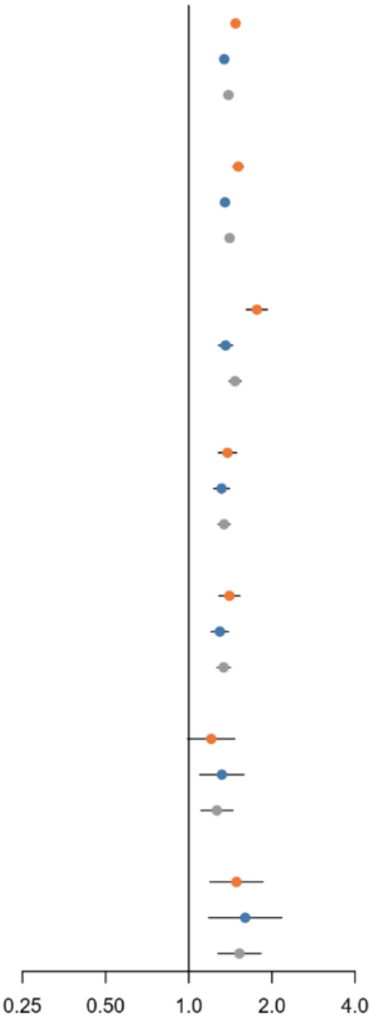

Cox regressions were performed in 315,073 UK Biobank participants and adjusted for sex and Townsend deprivation index (an area-based measure of socioeconomic status), including an interaction term between Townsend deprivation index and sex. Abbreviations: CI, confidence interval; HR, hazard ratio; RHR, ratio of hazard ratios.

Figure S5. Cox regression of the association between smoking continuation and risk of cardiovascular disease outcomes in females, males, and the overall population, using the same UK Biobank participants as included in MR analyses.

Outcome	No. of events	HR (95% CI)	RHR (95% CI)
**Cardiovascular disease**			
Females	12,641	1.67 (1.56, 1.78)	1.15 (1.06, 1.25)
Males	19,579	1.45 (1.38, 1.52)	
Overall	32,220	1.52 (1.47, 1.58)	
**Coronary heart disease**			
Females	9,528	1.63 (1.51, 1.75)	1.17 (1.07, 1.28)
Males	15,889	1.39 (1.31, 1.47)	
Overall	25,417	1.47 (1.40, 1.53)	
**Myocardial infarction**			
Females	2,143	2.27 (1.97, 2.61)	1.22 (1.03, 1.45)
Males	4,645	1.85 (1.68, 2.04)	
Overall	6,788	1.97 (1.82, 2.14)	
**Stroke**			
Females	3,113	1.82 (1.60, 2.08)	1.06 (0.89, 1.25)
Males	3,690	1.73 (1.55, 1.93)	
Overall	6,803	1.76 (1.62, 1.92)	
**Ischaemic stroke**			
Females	2,282	1.65 (1.41, 1.93)	0.93 (0.77, 1.14)
Males	3,055	1.77 (1.57, 2.00)	
Overall	5,337	1.72 (1.57, 1.90)	
**Intracerebral haemorrhage**			
Females	479	2.14 (1.51, 3.03)	1.53 (0.95, 2.46)
Males	467	1.40 (1.01, 1.94)	
Overall	946	1.69 (1.34, 2.15)	
**Subarachnoid haemorrhage**			
Females	352	2.55 (1.77, 3.66)	1.23 (0.68, 2.22)
Males	168	2.07 (1.29, 3.33)	
Overall	520	2.36 (1.77, 3.15)	

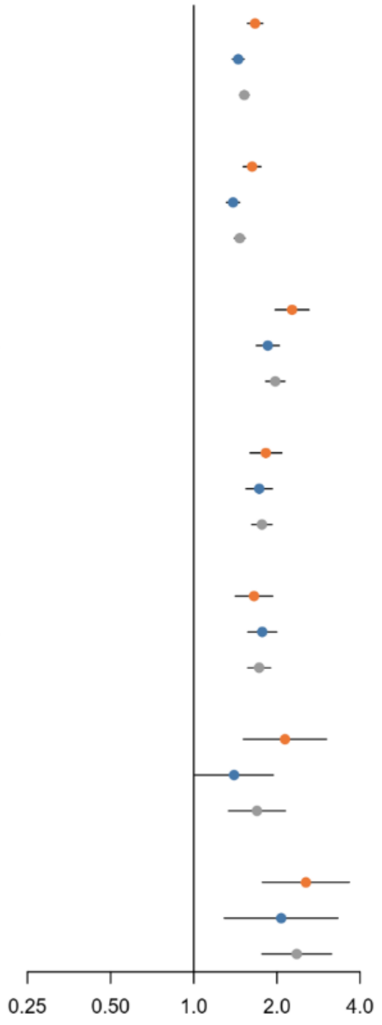

Cox regressions were performed in 315,073 UK Biobank participants and adjusted for sex and Townsend deprivation index (an area-based measure of socioeconomic status), including an interaction term between Townsend deprivation index and sex. Abbreviations: CI, confidence interval; HR, hazard ratio; RHR, ratio of hazard ratios.

Figure S6. Cox regression of the association between the number of cigarettes smoked per day and risk of cardiovascular disease outcomes in females, males, and the overall population, using the same UK Biobank participants as included in MR analyses.

Outcome	No. of events	HR (95% CI)	RHR (95% CI)
**Cardiovascular disease**			
Females	12,641	1.14 (1.10, 1.18)	1.23 (0.68, 2.22)
Males	19,579	1.11 (1.09, 1.14)	
Overall	32,220	1.12 (1.10, 1.14)	
**Coronary heart disease**			
Females	9,528	1.16 (1.11, 1.20)	1.23 (0.68, 2.22)
Males	15,889	1.11 (1.08, 1.14)	
Overall	25,417	1.12 (1.10, 1.14)	
**Myocardial infarction**			
Females	2,143	1.11 (1.03, 1.21)	1.23 (0.68, 2.22)
Males	4,645	1.06 (1.01, 1.11)	
Overall	6,788	1.07 (1.03, 1.11)	
**Stroke**			
Females	3,113	1.09 (1.01, 1.17)	1.23 (0.68, 2.22)
Males	3,690	1.12 (1.07, 1.18)	
Overall	6,803	1.11 (1.07, 1.16)	
**Ischaemic stroke**			
Females	2,282	1.13 (1.04, 1.23)	1.23 (0.68, 2.22)
Males	3,055	1.13 (1.07, 1.19)	
Overall	5,337	1.13 (1.08, 1.18)	
**Intracerebral haemorrhage**			
Females	479	0.94 (0.76, 1.15)	1.23 (0.68, 2.22)
Males	467	1.09 (0.95, 1.26)	
Overall	946	1.04 (0.93, 1.17)	
**Subarachnoid haemorrhage**			
Females	352	1.00 (0.81, 1.24)	1.23 (0.68, 2.22)
Males	168	1.05 (0.83, 1.33)	
Overall	520	1.02 (0.88, 1.20)	

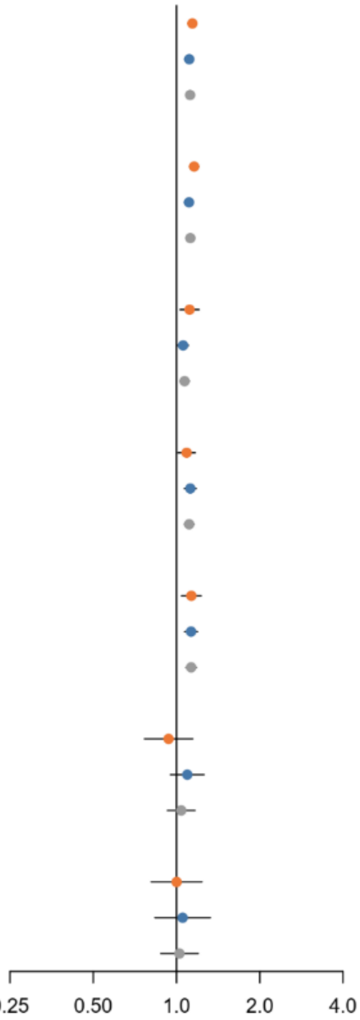

The variable representing number of cigarettes smoked per day was categorized into the following categories: 1=1-5, 2=6-15, 3=16-25, 4=26-35, 5=36+ and was analysed as a continuous variable. Cox regressions were performed in 315,073 UK Biobank participants and adjusted for sex and Townsend deprivation index (an area-based measure of socioeconomic status), including an interaction term between Townsend deprivation index and sex. Abbreviations: CI, confidence interval; HR, hazard ratio; RHR, ratio of hazard ratios.

Figure S7. Mendelian randomisation analysis of genetic liability to ever smoking and risk of cardiovascular disease outcomes in females based on different methods

Outcome/Method	OR (95% CI)	P−value
**Cardiovascular disease**		
IVW	1.81 (1.57, 2.07)	<0.001
Simple median	1.79 (1.49, 2.17)	<0.001
Weighted median	1.65 (1.36, 2.00)	<0.001
MR−Egger (PIntercept=0.161)	1.22 (0.70, 2.15)	0.483
**Coronary heart disease**		
IVW	1.86 (1.60, 2.17)	<0.001
Simple median	1.97 (1.60, 2.42)	<0.001
Weighted median	1.64 (1.32, 2.03)	<0.001
MR−Egger (PIntercept=0.120)	1.16 (0.63, 2.15)	0.626
**Myocardial infarction**		
IVW	2.08 (1.58, 2.73)	<0.001
Simple median	2.22 (1.53, 3.21)	<0.001
Weighted median	2.19 (1.49, 3.21)	<0.001
MR−Egger (PIntercept=0.386)	1.29 (0.43, 3.92)	0.652
**Stroke**		
IVW	1.62 (1.27, 2.05)	<0.001
Simple median	1.51 (1.10, 2.08)	0.012
Weighted median	1.44 (1.04, 2.00)	0.030
MR−Egger (PIntercept=0.727)	1.37 (0.52, 3.60)	0.528
**Ischaemic stroke**		
IVW	1.51 (1.15, 1.97)	0.003
Simple median	1.39 (0.97, 1.98)	0.072
Weighted median	1.39 (0.96, 2.00)	0.082
MR−Egger (PIntercept=0.939)	1.57 (0.53, 4.70)	0.417
**Intracerebral haemorrhage**		
IVW	1.24 (0.68, 2.24)	0.478
Simple median	1.05 (0.45, 2.45)	0.910
Weighted median	0.74 (0.31, 1.77)	0.502
MR−Egger (PIntercept=0.545)	0.60 (0.05, 6.76)	0.679
**Subarachnoid haemorrhage**		
IVW	2.49 (1.43, 4.36)	0.001
Simple median	3.77 (1.67, 8.49)	0.001
Weighted median	2.83 (1.24, 6.46)	0.013
MR−Egger (PIntercept=0.149)	0.49 (0.05, 4.78)	0.542

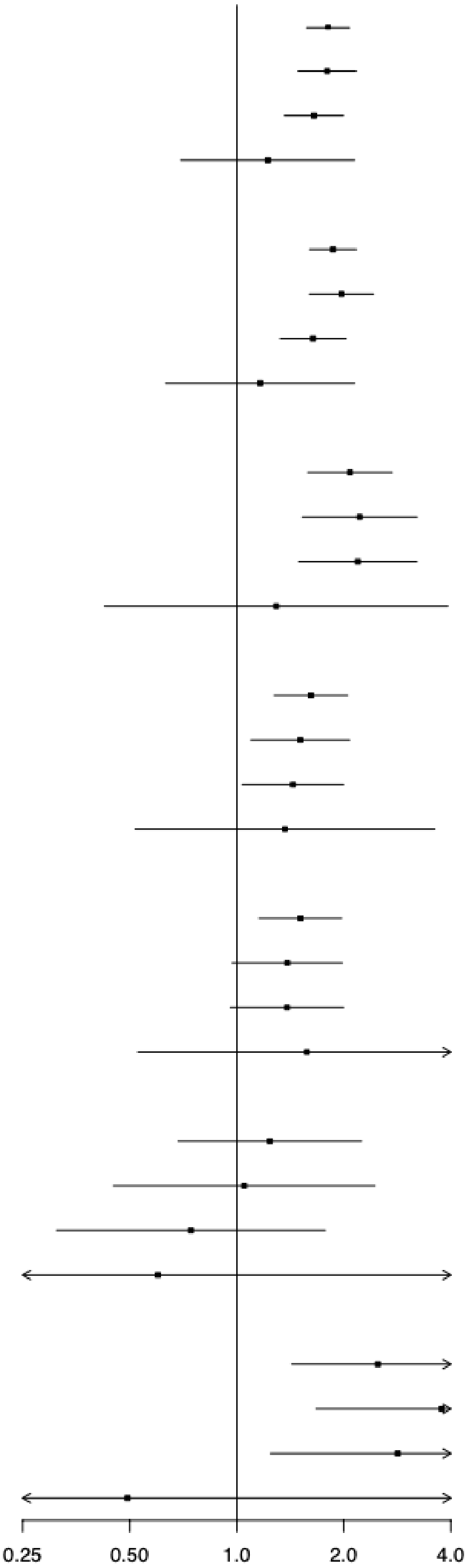

ORs can be interpreted as the effect per unit increase in log odds of genetic liability to ever smoking. Abbreviations: CI, confidence interval; IVW, inverse-variance weighting; MR, Mendelian randomisation; OR, odds ratio.

Figure S8. Mendelian randomisation analysis of genetic liability to ever smoking and risk of cardiovascular disease outcomes in males based on different methods

Outcome/Method	OR (95% CI)	P–value
**Cardiovascular disease**		
IVW	1.66 (1.46, 1.89)	<0.001
Simple median	1.67 (1.42, 1.96)	<0.001
Weighted median	1.70 (1.44, 2.01)	<0.001
MR–Egger (PIntercept=0.892)	1.60 (0.95, 2.71)	0.078
**Coronary heart disease**		
IVW	1.68 (1.46, 1.92)	<0.001
Simple median	1.61 (1.36, 1.91)	<0.001
Weighted median	1.67 (1.41, 2.00)	<0.001
MR–Egger (PIntercept=0.620)	1.46 (0.84, 2.55)	0.180
**Myocardial infarction**		
IVW	1.73 (1.46, 2.05)	<0.001
Simple median	1.77 (1.41, 2.21)	<0.001
Weighted median	1.86 (1.48, 2.34)	<0.001
MR–Egger (PIntercept=0.816)	1.60 (0.80, 3.20)	0.187
**Stroke**		
IVW	1.41 (1.16, 1.70)	<0.001
Simple median	1.36 (1.03, 1.78)	0.028
Weighted median	1.36 (1.03, 1.78)	0.029
MR–Egger (PIntercept=0.421)	1.92 (0.88, 4.16)	0.100
**Ischaemic stroke**		
IVW	1.48 (1.20, 1.81)	<0.001
Simple median	1.56 (1.17, 2.09)	0.003
Weighted median	1.51 (1.13, 2.03)	0.005
MR–Egger (PIntercept=0.575)	1.86 (0.81, 4.28)	0.144
**Intracerebral haemorrhage**		
IVW	0.96 (0.57, 1.62)	0.887
Simple median	0.65 (0.31, 1.39)	0.268
Weighted median	0.85 (0.40, 1.82)	0.671
MR–Egger (PIntercept=0.133)	4.68 (0.56, 39.18)	0.155
**Subarachnoid haemorrhage**		
IVW	0.96 (0.47, 1.94)	0.902
Simple median	0.76 (0.27, 2.15)	0.604
Weighted median	0.87 (0.31, 2.45)	0.800
MR–Egger (PIntercept=0.254)	4.86 (0.27, 87.11)	0.283

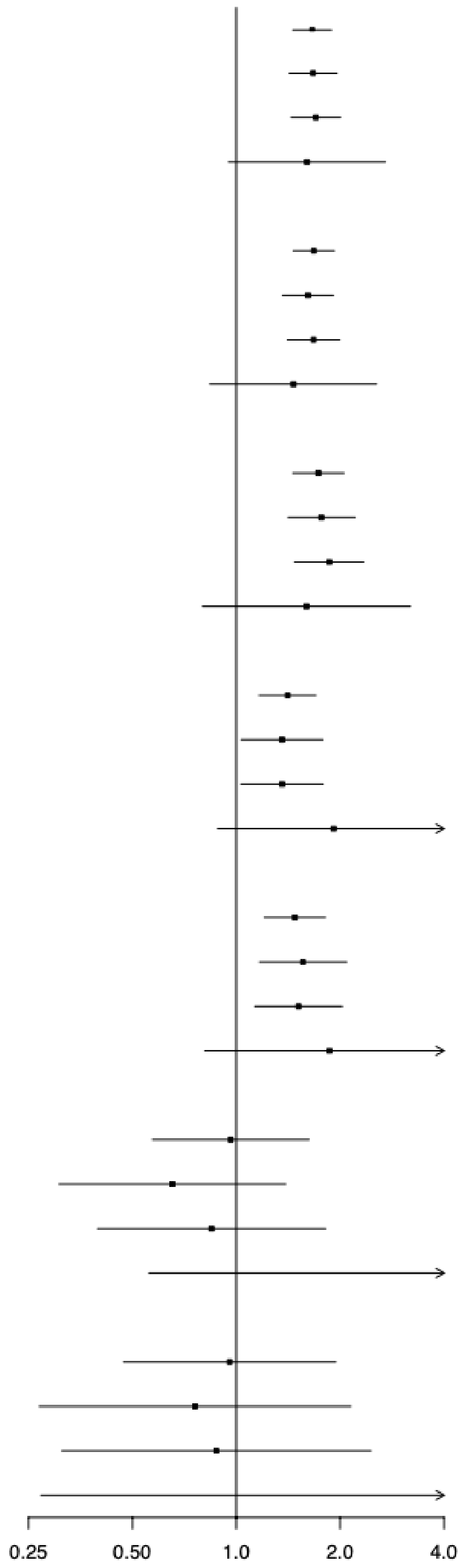

ORs can be interpreted as the effect per unit increase in log odds of genetic liability to ever smoking. Abbreviations: CI, confidence interval; IVW, inverse-variance weighting; MR, Mendelian randomisation; OR, odds ratio.

Figure S9. Mendelian randomisation analysis of genetic liability to ever smoking and risk of cardiovascular disease outcomes in females and males based on different methods

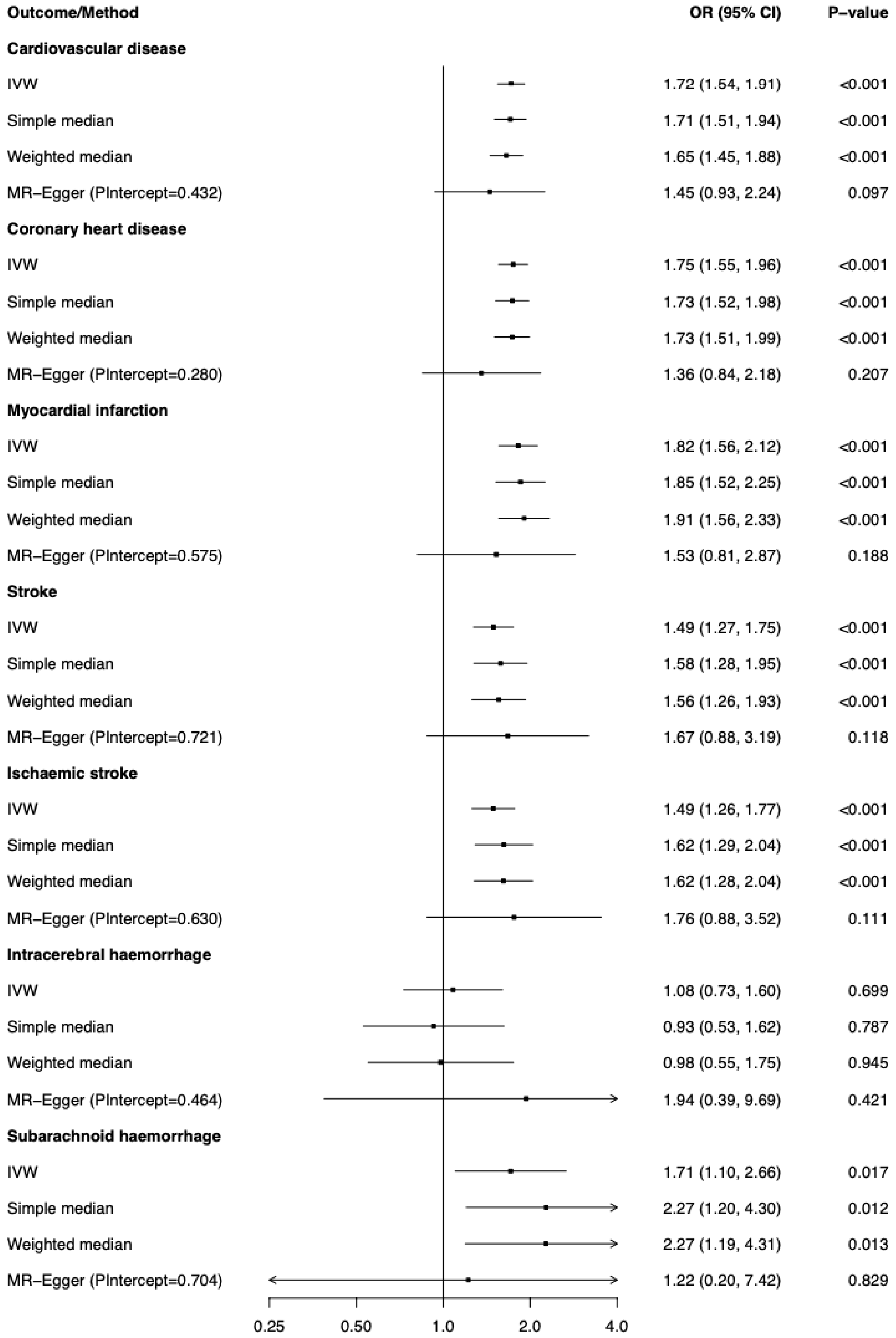

ORs can be interpreted as the effect per unit increase in log odds of genetic liability to ever smoking. Abbreviations: CI, confidence interval; IVW, inverse-variance weighting; MR, Mendelian randomisation; OR, odds ratio.

Figure S10. Mendelian randomisation analysis of genetic liability to smoking continuation and risk of cardiovascular disease outcomes in females based on different methods

Outcome/Method	OR (95% CI)	P−value
**Cardiovascular disease**		
IVW	1.34 (0.91, 1.97)	0.139
Simple median	1.04 (0.64, 1.69)	0.877
Weighted median	1.06 (0.67, 1.66)	0.803
MR−Egger (PIntercept=0.530)	1.81 (0.65, 5.01)	0.254
**Coronary heart disease**		
IVW	1.49 (1.00, 2.23)	0.050
Simple median	1.37 (0.82, 2.30)	0.230
Weighted median	1.49 (0.92, 2.41)	0.107
MR−Egger (PIntercept=0.877)	1.61 (0.55, 4.68)	0.380
**Myocardial infarction**		
IVW	2.04 (1.09, 3.85)	0.027
Simple median	2.88 (1.24, 6.69)	0.014
Weighted median	2.16 (0.97, 4.82)	0.060
MR−Egger (PIntercept=0.999)	2.04 (0.38, 11.03)	0.407
**Stroke**		
IVW	1.00 (0.60, 1.64)	0.988
Simple median	0.63 (0.30, 1.35)	0.237
Weighted median	1.02 (0.50, 2.06)	0.960
MR−Egger (PIntercept=0.153)	2.33 (0.66, 8.22)	0.190
**Ischaemic stroke**		
IVW	1.05 (0.63, 1.74)	0.849
Simple median	0.71 (0.33, 1.54)	0.386
Weighted median	0.96 (0.46, 2.01)	0.914
MR−Egger (PIntercept=0.288)	2.03 (0.54, 7.53)	0.292
**Intracerebral haemorrhage**		
IVW	0.71 (0.19, 2.67)	0.612
Simple median	0.60 (0.09, 4.11)	0.607
Weighted median	0.58 (0.09, 3.64)	0.564
MR−Egger (PIntercept=0.793)	1.10 (0.03, 38.24)	0.958
**Subarachnoid haemorrhage**		
IVW	1.50 (0.40, 5.70)	0.549
Simple median	0.63 (0.09, 4.48)	0.641
Weighted median	1.44 (0.24, 8.55)	0.689
MR−Egger (PIntercept=0.027)	38.61 (1.70, 875.36)	0.022

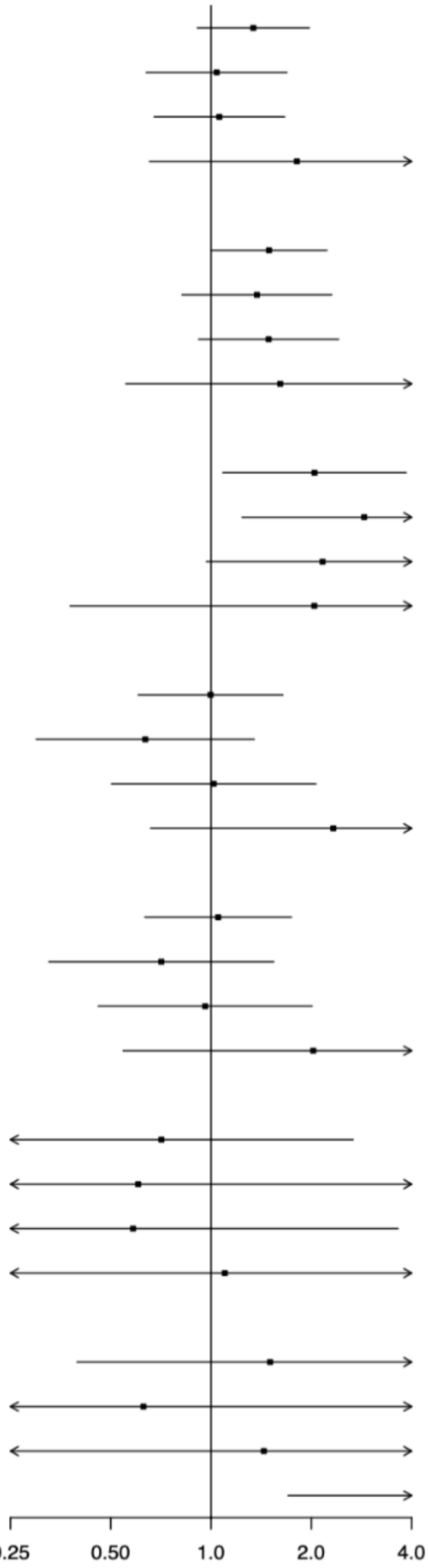

ORs can be interpreted as the effect per unit increase genetic liability to smoking continuation among ever smokers. Abbreviations: CI, confidence interval; IVW, inverse-variance weighting; MR, Mendelian randomisation; OR, odds ratio.

Figure S11. Mendelian randomisation analysis of genetic liability to smoking continuation and risk of cardiovascular disease outcomes in males based on different methods

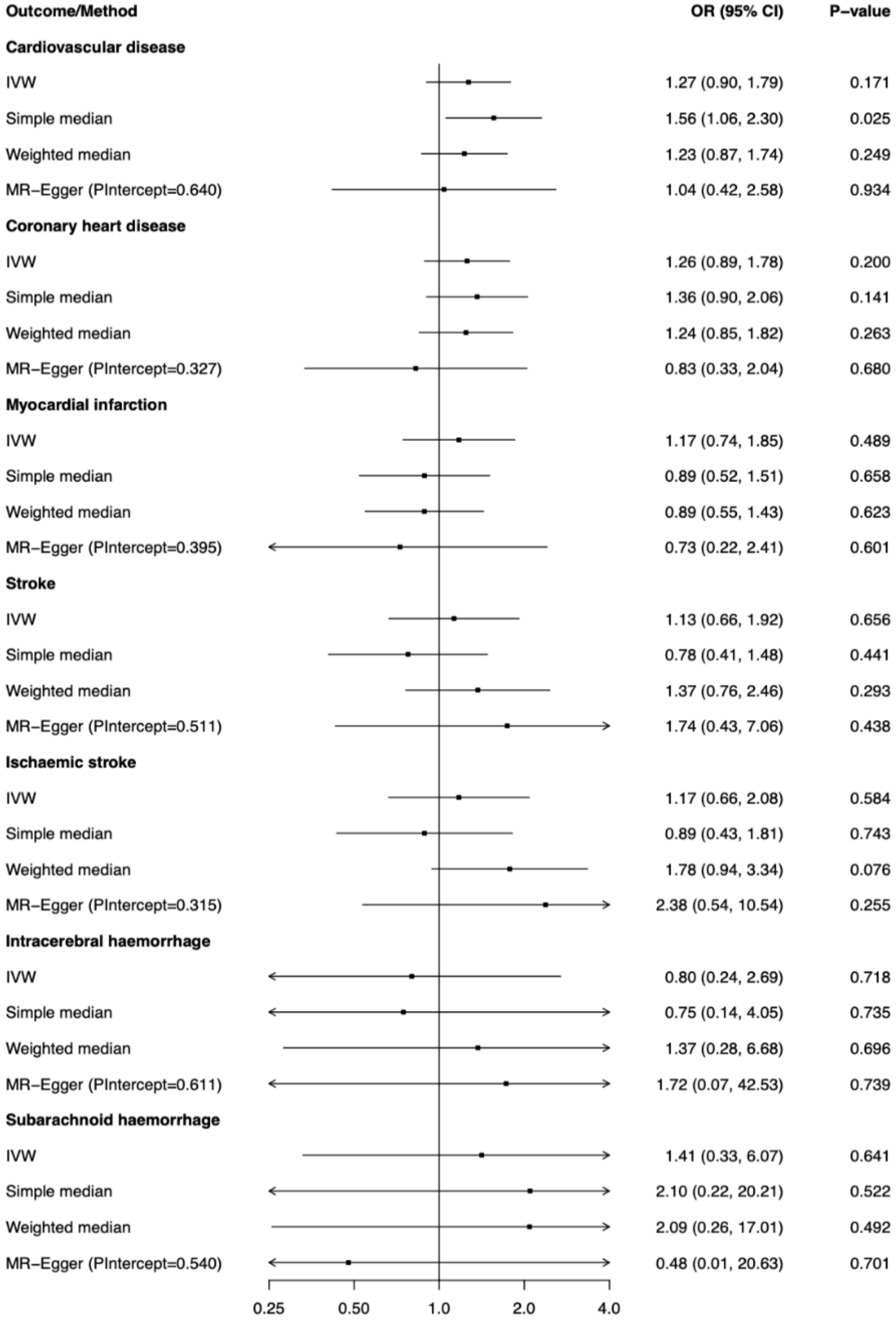

ORs can be interpreted as the effect per unit increase genetic liability to smoking continuation among ever smokers. Abbreviations: CI, confidence interval; IVW, inverse-variance weighting; MR, Mendelian randomisation; OR, odds ratio.

Figure S12. Mendelian randomisation analysis of genetic liability to smoking continuation and risk of cardiovascular disease outcomes in females and males based on different methods

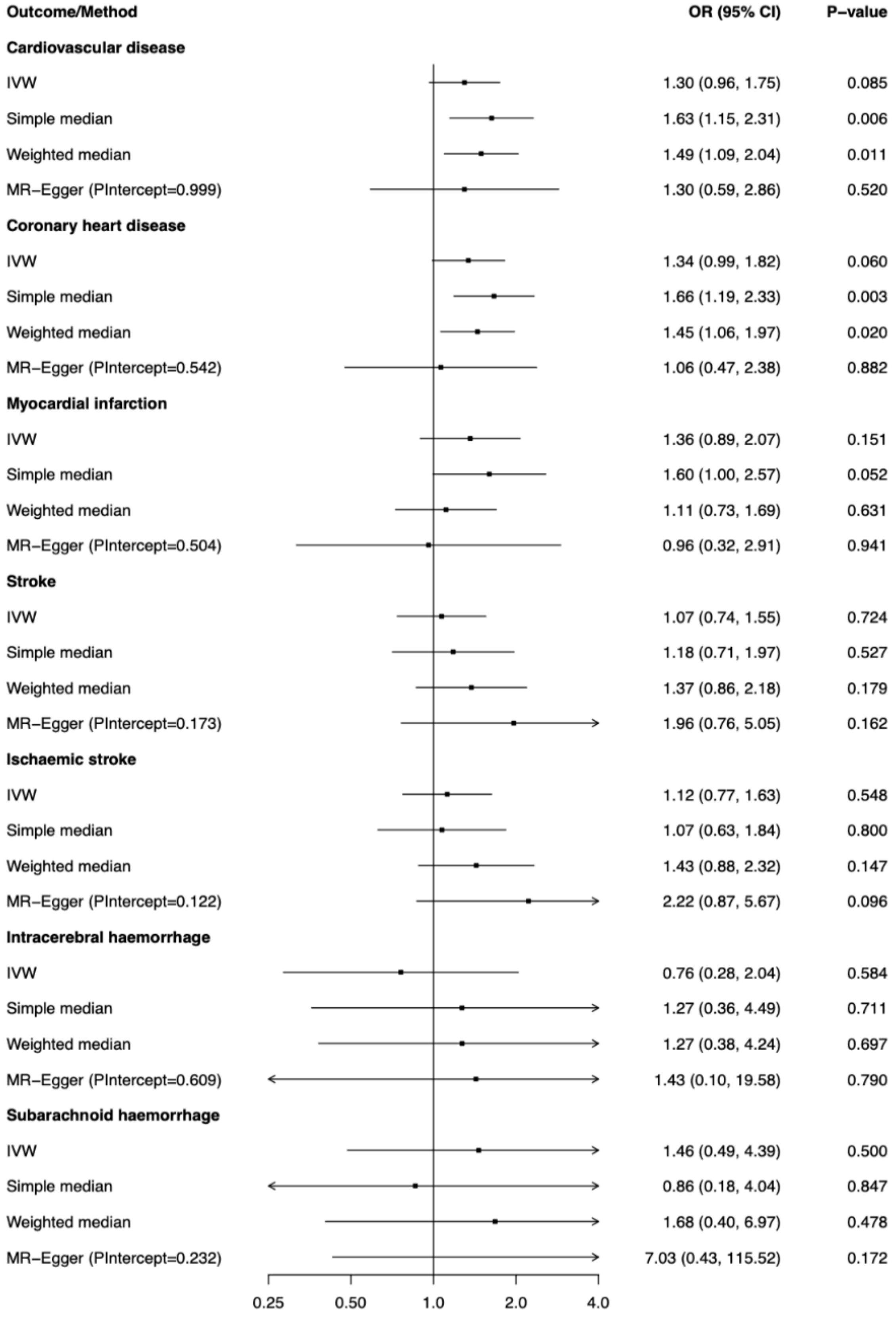

ORs can be interpreted as the effect per unit increase genetic liability to smoking continuation among ever smokers. Abbreviations: CI, confidence interval; IVW, inverse-variance weighting; MR, Mendelian randomisation; OR, odds ratio.

Figure S13. Mendelian randomisation analysis of genetically proxied <u>number of cigarettes per day</u> and risk of cardiovascular disease outcomes <u>in females</u> based on different methods

Outcome/Method	OR (95% CI)	P–value
**Cardiovascular disease**		
IVW	1.44 (1.19, 1.73)	<0.001
Simple median	2.00 (1.50, 2.66)	<0.001
Weighted median	1.08 (0.88, 1.33)	0.465
MR–Egger (PIntercept=<0.001)	0.85 (0.65, 1.12)	0.261
**Coronary heart disease**		
IVW	1.61 (1.31, 1.97)	<0.001
Simple median	2.43 (1.78, 3.31)	<0.001
Weighted median	1.18 (0.95, 1.48)	0.131
MR–Egger (PIntercept=<0.001)	1.00 (0.73, 1.37)	0.998
**Myocardial infarction**		
IVW	1.66 (1.18, 2.35)	0.004
Simple median	2.04 (1.22, 3.43)	0.007
Weighted median	1.41 (0.92, 2.15)	0.116
MR–Egger (PIntercept=0.029)	0.98 (0.55, 1.75)	0.936
**Stroke**		
IVW	1.12 (0.88, 1.43)	0.360
Simple median	1.89 (1.19, 3.01)	0.007
Weighted median	0.79 (0.57, 1.10)	0.160
MR–Egger (PIntercept=<0.001)	0.59 (0.40, 0.88)	0.011
**Ischaemic stroke**		
IVW	1.16 (0.88, 1.54)	0.279
Simple median	1.59 (0.96, 2.64)	0.070
Weighted median	0.84 (0.58, 1.22)	0.363
MR–Egger (PIntercept=0.002)	0.65 (0.42, 1.02)	0.061
**Intracerebral haemorrhage**		
IVW	0.96 (0.51, 1.77)	0.884
Simple median	1.33 (0.43, 4.16)	0.622
Weighted median	0.53 (0.21, 1.32)	0.172
MR–Egger (PIntercept=0.057)	0.40 (0.14, 1.19)	0.099
**Subarachnoid haemorrhage**		
IVW	1.11 (0.61, 2.00)	0.730
Simple median	1.13 (0.40, 3.18)	0.824
Weighted median	1.13 (0.45, 2.82)	0.801
MR–Egger (PIntercept=0.491)	0.82 (0.29, 2.32)	0.712

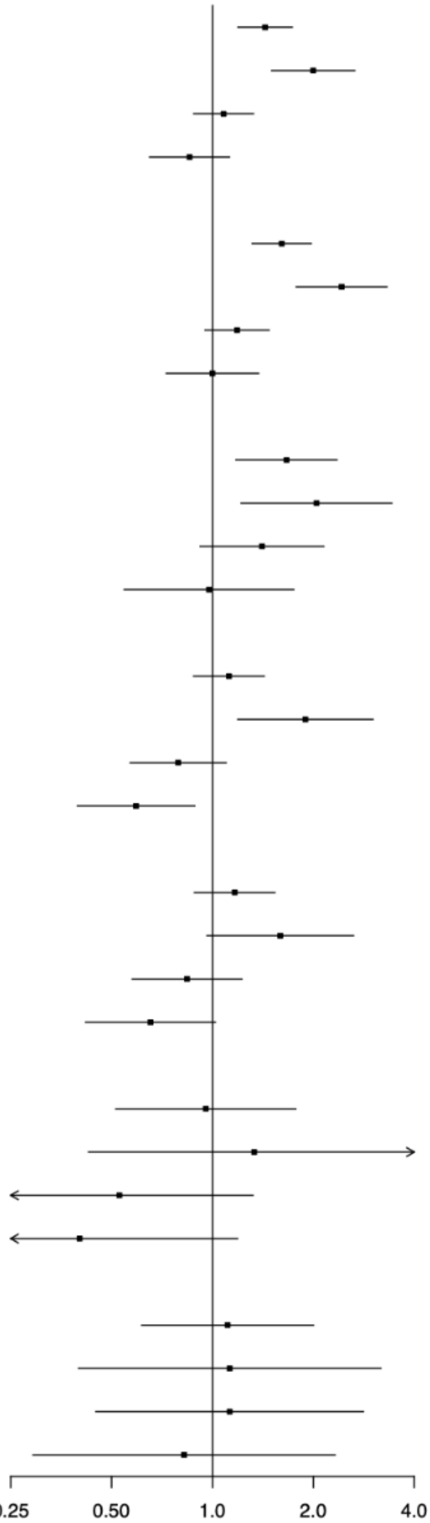

The variable representing number of cigarettes smoked per day was categorized into the following categories: 1=1-5, 2=6-15, 3=16-25, 4=26-35, 5=36+ and was analysed as a continuous variable. ORs can be interpreted as the effect per category increase of genetically proxied number of cigarettes smoked per day among ever smokers. Abbreviations: CI, confidence interval; IVW, inverse-variance weighting; MR, Mendelian randomisation; OR, odds ratio.

Figure S14. Mendelian randomisation analysis of genetically proxied number of cigarettes per day and risk of cardiovascular disease outcomes in males based on different methods

Outcome/Method	OR (95% CI)	P–value
**Cardiovascular disease**		
IVW	1.29 (1.06, 1.59)	0.013
Simple median	1.61 (1.27, 2.04)	<0.001
Weighted median	1.14 (0.93, 1.40)	0.198
MR–Egger (PIntercept=<0.001)	0.80 (0.58, 1.09)	0.154
**Coronary heart disease**		
IVW	1.31 (1.04, 1.65)	0.020
Simple median	1.60 (1.24, 2.06)	<0.001
Weighted median	1.25 (1.01, 1.54)	0.040
MR–Egger (PIntercept=<0.001)	0.78 (0.55, 1.12)	0.183
**Myocardial infarction**		
IVW	1.33 (1.03, 1.72)	0.031
Simple median	1.95 (1.38, 2.75)	<0.001
Weighted median	0.87 (0.67, 1.13)	0.308
MR–Egger (PIntercept=<0.001)	0.67 (0.45, 0.98)	0.039
**Stroke**		
IVW	1.22 (0.97, 1.54)	0.084
Simple median	1.74 (1.17, 2.60)	0.007
Weighted median	1.01 (0.75, 1.38)	0.933
MR–Egger (PIntercept=0.048)	0.89 (0.60, 1.31)	0.547
**Ischaemic stroke**		
IVW	1.29 (1.03, 1.62)	0.028
Simple median	2.13 (1.40, 3.24)	<0.001
Weighted median	1.08 (0.78, 1.51)	0.644
MR–Egger (PIntercept=0.030)	0.91 (0.62, 1.34)	0.636
**Intracerebral haemorrhage**		
IVW	0.78 (0.42, 1.47)	0.447
Simple median	0.66 (0.25, 1.76)	0.406
Weighted median	0.54 (0.23, 1.22)	0.139
MR–Egger (PIntercept=0.906)	0.74 (0.24, 2.27)	0.599
**Subarachnoid haemorrhage**		
IVW	1.70 (0.79, 3.65)	0.177
Simple median	1.44 (0.38, 5.56)	0.593
Weighted median	3.24 (1.09, 9.62)	0.034
MR–Egger (PIntercept=0.322)	2.94 (0.78, 11.13)	0.113

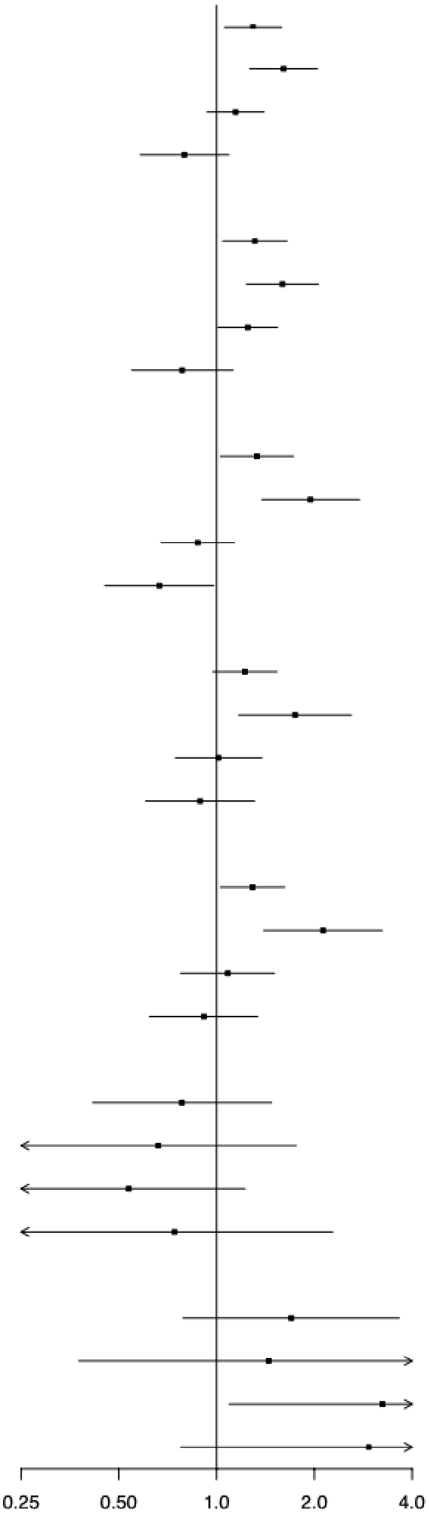

The variable representing number of cigarettes smoked per day was categorized into the following categories: 1=1-5, 2=6-15, 3=16-25, 4=26-35, 5=36+ and was analysed as a continuous variable. ORs can be interpreted as the effect per category increase of genetically proxied number of cigarettes smoked per day among ever smokers. Abbreviations: CI, confidence interval; IVW, inverse-variance weighting; MR, Mendelian randomisation; OR, odds ratio.

Figure S15. Mendelian randomisation analysis of genetically proxied <u>number of cigarettes per day</u> and risk of cardiovascular disease outcomes <u>in females and males</u> based on different methods

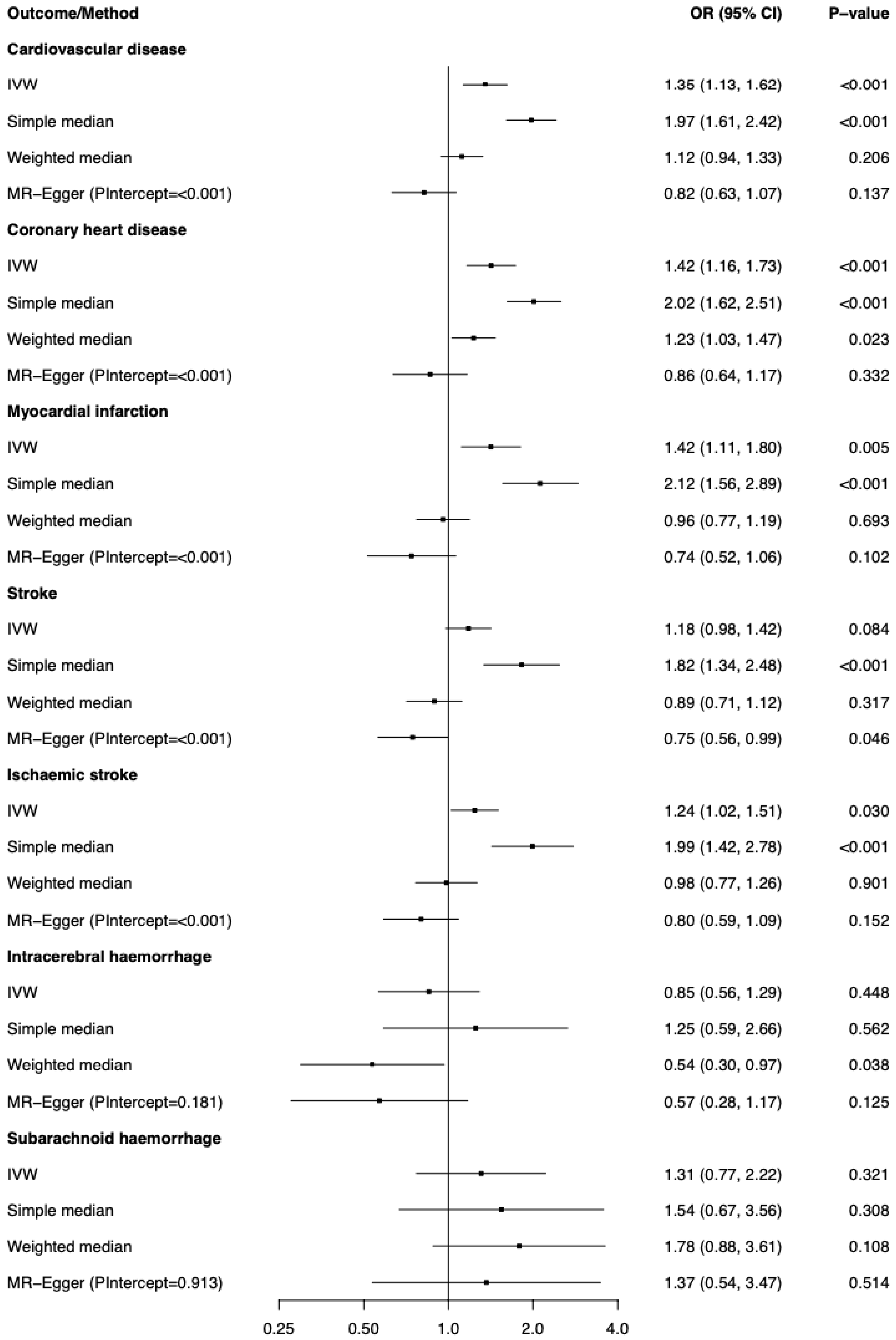

The variable representing number of cigarettes smoked per day was categorized into the following categories: 1=1-5, 2=6-15, 3=16-25, 4=26-35, 5=36+ and was analysed as a continuous variable. ORs can be interpreted as the effect per category increase of genetically proxied number of cigarettes smoked per day among ever smokers. Abbreviations: CI, confidence interval; IVW, inverse-variance weighting; MR, Mendelian randomisation; OR, odds ratio.
